# Stability of Multi-Parametric Prostate MRI Radiomic Features to Variations in Segmentation

**DOI:** 10.3390/jpm13071172

**Published:** 2023-07-22

**Authors:** Sithin Thulasi Seetha, Enrico Garanzini, Chiara Tenconi, Cristina Marenghi, Barbara Avuzzi, Mario Catanzaro, Silvia Stagni, Sergio Villa, Barbara Noris Chiorda, Fabio Badenchini, Elena Bertocchi, Sebastian Sanduleanu, Emanuele Pignoli, Giuseppe Procopio, Riccardo Valdagni, Tiziana Rancati, Nicola Nicolai, Antonella Messina

**Affiliations:** 1 Prostate Cancer Program, Fondazione IRCCS Istituto Nazionale dei Tumori, 20133 Milan, Italy; s.thulasiseetha@maastrichtuniversity.nl (S.T.S.); riccardo.valdagni@istitutotumori.mi.it (R.V.); 2 Department of Precision Medicine, GROW—School for Oncology and Developmental Biology, Maastricht University, 6211 LK Maastricht, The Netherlands; 3 Department of Radiology, Fondazione IRCCS Istituto Nazionale dei Tumori, 20133 Milan, Italy; enrico.garanzini@istitutotumori.mi.it (E.G.); antonella.messina@istitutotumori.mi.it (A.M.); 4 Department of Medical Physics, Fondazione IRCCS Istituto Nazionale dei Tumori, 20133 Milan, Italy; emanuele.pignoli@istitutotumori.mi.it; 5 Department of Oncology and Hematooncology, Università degli Studi di Milano, 20133 Milan, Italy; 6 Unit of Genito-Urinary Medical Oncology, Fondazione IRCCS Istituto Nazionale dei Tumori, 20133 Milan, Italy; cristina.marenghi@istitutotumori.mi.it (C.M.); fabio.badenchini@istitutotumori.mi.it (F.B.); elena.bertocchi@istitutotumori.mi.it (E.B.); giuseppe.procopio@istitutotumori.mi.it (G.P.); 7 Department of Radiation Oncology, Fondazione IRCCS Istituto Nazionale dei Tumori, 20133 Milan, Italy; barbara.avuzzi@istitutotumori.mi.it (B.A.); sergio.villa@istitutotumori.mi.it (S.V.); barbara.noris@istitutotumori.mi.it (B.N.C.); 8 Department of Urology, Fondazione IRCCS Istituto Nazionale dei Tumori, 20133 Milan, Italy; mario.catanzaro@istitutotumori.mi.it (M.C.); silvia.stagni@istitutotumori.mi.it (S.S.); nicola.nicolai@istitutotumori.mi.it (N.N.); 9 Data Science Unit, Fondazione IRCCS Istituto Nazionale dei Tumori, 20133 Milan, Italy

**Keywords:** radiomics, multi-parametric MRI, prostate

## Abstract

Stability analysis remains a fundamental step in developing a successful imaging biomarker to personalize oncological strategies. This study proposes an in silico contour generation method for simulating segmentation variations to identify stable radiomic features. Ground-truth annotation provided for the whole prostate gland on the multi-parametric MRI sequences (T2w, ADC, and SUB-DCE) were perturbed to mimic segmentation differences observed among human annotators. In total, we generated 15 synthetic contours for a given image-segmentation pair. One thousand two hundred twenty-four unfiltered/filtered radiomic features were extracted applying Pyradiomics, followed by stability assessment using ICC(1,1). Stable features identified in the internal population were then compared with an external population to discover and report robust features. Finally, we also investigated the impact of a wide range of filtering strategies on the stability of features. The percentage of unfiltered (filtered) features that remained robust subjected to segmentation variations were T2w—36% (81%), ADC—36% (94%), and SUB—43% (93%). Our findings suggest that segmentation variations can significantly impact radiomic feature stability but can be mitigated by including pre-filtering strategies as part of the feature extraction pipeline.

## 1. Introduction

Multi-parametric MRI (mpMRI), including T2-weighted (T2w), diffusion-weighted imaging (DWI), and dynamic contrast-enhanced (DCE) images, has become an essential tool for the detection and characterization of prostate cancer (PCa) [1,2,3]. Its role has extended beyond tumor staging to encompass cancer detection and monitoring of disease progression during active surveillance (AS) [4,5,6]. The use of mpMRI in AS seems particularly attractive. Combining morphological and functional images constitutes a non-invasive tool for longitudinal monitoring of patients, interrogating the entire prostate volume, and possibly giving information on the indolence or aggressiveness of the prostate tissue. Presently, mpMRI is widely used in AS to assess an image-based risk stratification score following the guidelines from the prostate imaging reporting and data system (PI-RADS) [7]. However, this semi-quantitative approach relies entirely on standardized acquisition and reporting guidelines.

The increasing use of mpMRI among patients in active surveillance makes radiomics highly attractive. Radiomics is a quantitative approach to medical image analysis that aims to capture information beyond what is visible to the naked eye [8,9]. Only a handful of studies have investigated the utility of mpMRI radiomics features in the premise of AS. Sushentsev, Nikita, et al. [10] examined the complementary value of radiomics features to improve baseline prediction of PCa progression. Another study by Sushentsev et al. [11] compared the performance of delta-radiomics [12] and MRI-derived PRECISE [13] scores in progression prediction. Algohary, Ahmad, et al. [14] evaluated the performance of radiomics in identifying the presence of clinically significant PCa in AS patients. A few other studies [15,16] focused on clinical features and/or a chosen set of shape and first-order features extracted from MRI sequences for progression prediction. Albeit, these studies only included patients with MRI visible lesions wherein lesions served as the ROI for extracting radiomics features. Since around half of the patients in AS population are likely to show MR-visible lesions [17,18,19], this excludes almost half of the patients enrolled in surveillance studies.

Furthermore, these studies only considered features extracted from bi-parametric (bp) MRI—T2w and ADC (derived from DWI) for predictive modeling. Notably, DCE is acquired as part of routine AS protocol following PIRADS specification. Excluding DCE sequences results in losing readily available diagnostic information [20].

Generally, these image-based signatures must be highly robust to develop reliable models for routine clinical practice. Developing a robust model means setting radiomics in the “big data” analysis framework. Such a model requires extensive training and validation sets from multicentric studies with image data derived from a large patient population for a specific pathology. This introduces the complication of radiomic feature variability due to differences in scanners [21,22], imaging acquisition parameters/protocols [23], reconstruction algorithms [24,25], processing pipelines [26,27], and the annotation of the region of interest (ROI) [28,29]. The variability due to these sources may hide any potential signal from tumor biology, making at least some of the radiomic signatures unreliable and thus hindering the generalization of results.

Delineating the ROI is an essential step before all image-based medical interventions. This is a tedious task, and even in the best scenario (segmentation carried out following standardized and quantitative guidelines), inter- and/or intra-observer variability among trained radiologists is observed. We may attribute these differences to the behavior of radiologists in a clinical setting, where some are more conservative or liberal regarding segmentation. Often, a slight difference in ROIs results in different radiomics feature values [30,31,32], commonly referred to as feature instability. Developing signatures using such unstable features ultimately leads to lower robustness of signatures. Although many studies have already been carried out to tackle this issue, there are only a few studies in the context of AS. For example, Xu, Lili, et al. [33] and Zhang, Gu-mu-yang, et al. [34] included feature stability assessment to variations in lesion segmentation as a feature filtering strategy earlier in their predictive pipeline. Two radiologists were involved in these studies, and ICC [35] was used as the statistical metric to measure stability. Conversely, Chen, Tong, et al. [36] merged manual lesion segmentations by two radiologists before feature extraction to reduce the impact of inter-observer variations. Fehr, Duc, et al. [37] employed a segmentation approach where three readers were involved in a consensual delineation of tumor and non-cancerous prostate regions as part of their study.

Generally, it is recommended to include at least three raters in stability assessments [35]. In studies measuring feature stability to segmentation variation, this number usually falls within the range of 2–5 [38,39,40]. However, obtaining multiple radiologists for segmentation is a challenging task and is often infeasible. One solution could be the application of morphological operations on the ROI to generate perturbed segmentations for feature stability assessment. Sushentsev, Nikita, et al. [10,11] followed this strategy where two versions of ROI were generated by subjecting the original ROI to morphological operations—opening and closing using a spherical structuring element of a 1-pixel radius. However, this method does not approximate clinical inter/intra-observer variations wherein the differences are non-deterministic. An alternative solution was proposed by Haarburger et al. using a probabilistic U-Net model, which was used to generate 25 plausible segmentations [41]. Using this approach, they discovered a set of features stable to variations in segmentation. However, the probabilistic U-Net model suffered from limited segmentation diversity, which can bias the results. A recent extension to this was proposed by the same authors who used PHiseg model to address some of the limitations of their previous work [29]; they even included four radiologists for clinical inter/intra-observer variations analysis. Although using such generative models can scale up such studies, there are a few caveats. The computation cost and resource requirement for training and tuning a generative deep learning (DL) model is quite high. Expertise in DL is also essential to customize and integrate such a model into a radiomics pipeline. 

In this work, we try to address these limitations by proposing a simple in silico contour generation method inspired by the data augmentation paradigm in DL. We have considered the whole prostate gland as the ROI to endorse the inclusion of AS patients with no MR-visible lesions for future predictive modeling studies. On this account, we also considered the acquired DCE sequence as part of our pipeline routine AS protocol. We intend to simulate various clinical segmentation scenarios using a combination of linear transformations such as rotation, scaling, and shifting that follows a set of predetermined constraints to simulate the behavior of manual annotators. The stable features identified in the internal population will then be compared with an external dataset to report a set of overlapping stable features (i.e., robust features) that could be utilized in future predictive modeling studies. 

## 2. Materials and Methods

### 2.1. Datasets

#### 2.1.1. Internal Dataset

We included one hundred patients diagnosed with very low-risk prostate cancer and enrolled in active surveillance at the Fondazione IRCCS Istituto Nazionale dei Tumori in Milan. The local Ethics Committee approved the study protocol (INT 113/16, INT 46/07, and INT 95/11), and all patients signed a written informed consent for the study.

MRI acquisitions were performed using an “Ingenia” 1.5 T (Philips Medical System, Best, The Netherlands) equipped with 32-channel phased-array and spine coils in combination with an endorectal receiver coil. Images were acquired using Turbo Spin Echo and Gradient Echo sequences, always including a sequence with axial slicing, according to the PI-RADS v2.1 [7] recommendations. The acquisition protocol was standardized: every set of images included T2w images (TR/TE = 4910/110 ms, slice thickness = 3 mm, pixel spacing = 0.297 mm) and two functional MRI sequences: DWI (b-values of 0, 1500, and 2000 s/mm^2^, TR/TE = 3320/106 ms, slice thickness = 3 mm, pixel spacing = 1.250 mm) and DCE (TR/TE = 4.03/1.88 ms, slice thickness = 3 mm, pixel spacing = 1.136 mm). DCE was acquired with a high temporal resolution (<10 s) during the administration of the contrast agent in the same position and phase encoding direction as T2w and DWI. 

An experienced radiologist (E.G.) segmented the entire prostate gland on the T2w sequence. Interpolation proved sufficient to align the segmentation on the T2w with the other sequences, owed to the restricted motion and sequential acquisition of all the multi-parametric sequences. 

**Figure 1:**
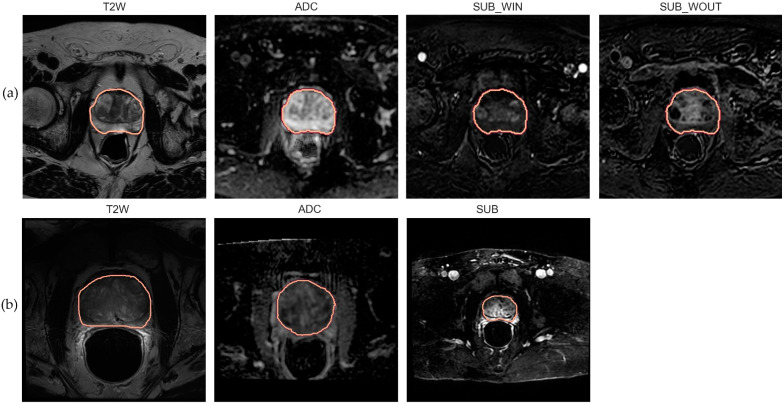
Mid-level axial slice of the prostate gland with ROI annotation. (**a**) T2w, ADC, SUBwin, and SUBwout images associated with a random patient sampled from the internal population; (**b**) T2w, ADC, and SUB images associated with a random patient sampled from the external population.

We processed DWI and DCE sequences to generate the Apparent Diffusion Coefficient (ADC) and subtraction (SUB) maps. We derived the ADC map by computing the negative gradient associated with a least-square fit (straight line) over the DWI acquisitions with three b-values—0, 1500, and 2000 mm/s^2^. We processed the DCE acquisitions to generate two subtraction (SUB) maps describing the wash-in (SUBwin) and wash-out (SUBwout) phases of the contrast agent. The maps were computed by splitting the DCE acquisitions at a time point close to 90s in the temporal domain, i.e., SUBwin = DCE_90+ε_-DCE_0_ and SUBwout = DCE_t_n_-DCE_90+ε_. This was performed to capture the contrast agent inflow (wash-in) and outflow (wash-out) phases which are known to guide radiologists in assessing the malignancy in PCa management [42]. Here, t_n indicates the last DCE acquisition in the temporal domain. ε represents the deviation from the referenced time point. Table 1a presents a simplified summary of the internal dataset properties, and Figure 1, panel (a) highlights mid-gland level axial mpMRI slices of a sample patient from the population.

#### 2.1.2. External Dataset

QIN Prostate Repeatability is an open-source [43,44,45] prostate mpMRI test-retest dataset of 15 men with confirmed or suspected prostate cancer. mpMRI acquisitions were PI-RADS v2 compliant and were performed using “GE Signa HDxt platform” and “GE Discovery MR750w” (General Electric Healthcare, Milwaukee, WI) machines. The images were acquired at a magnetic strength of 3.0 T in combination with an endorectal coil. Two scanners were used because of the hardware upgrade during the study. For each patient, the baseline and repeated examinations were taken on the same scanner at a two-week interval. Multi-parametric acquisitions included axial T2w images (TR/TE = 3350–5109/84–107 ms, slice thickness = 3 mm), DWI (b-values of 0 and 1400 s/mm^2^, TR/TE = 2500–8150/76.7–80.6 ms, slice thickness = 3–4 mm) and DCE (TR/TE = 3.68–4.1/1.3–1.42 ms, slice thickness = 5–6 mm) sequences. 

The in-built scanner software generated ADC and DCE SUB maps. The SUB map was computed as the difference between the phases involved in contrast bolus arrival to the baseline. Ultimately manual segmentation of the whole prostate gland (amongst other ROIs) was performed by an experienced radiologist for each sequence and was included in the dataset. Table 1b presents a simplified summary of the external dataset properties, and Figure 1, panel (b), highlights mid-gland level axial mpMRI slices of a sample patient from the population.

### 2.2. In Silico Contour Generation

To evaluate the impact of segmentation variations on radiomic feature stability, we synthetically generated 15 new prostate ROIs for each patient. We synthesized these contours by subjecting the manual ROI segmentation to bounded perturbations using affine transformations. The transformations include shifting, scaling, and rotation to simulate under-/over-segmentation variations. This approach was inspired by the data augmentation technique commonly used in deep learning [46,47]. TorchIO (v0.18.21) [48], a Python-based library for processing or augmenting 3D medical images, was used to generate synthetic contours dynamically. 

By considering bounded (i.e., constrained) combinations of affine transformations, we systematically analyzed three categories of contour augmentations: in-plane, out-plane, and in and out-plane on each mpMRI sequence. 

**Figure 2:**
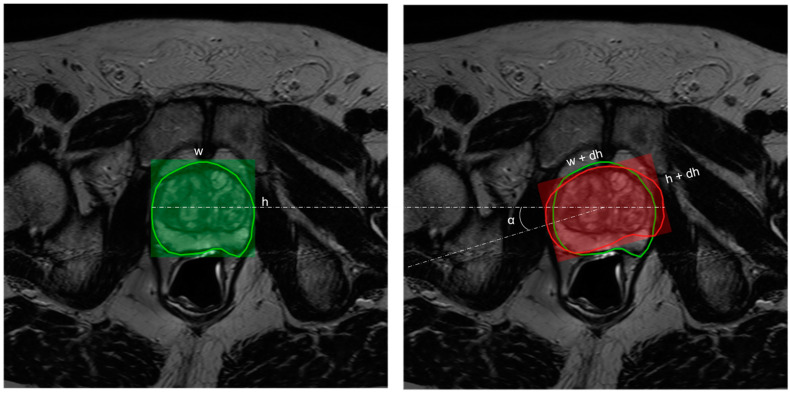
Illustration of in-plane augmentation. The width, w, and height, h, associated with the contour drawn by the radiologist (left in green) in the axial plane are allowed to undergo random perturbation by a delta value—dw, dh ~ U (−2.7 mm, 2.7 mm). This results in a transformed contour (right in red) with width, w’ = w + dw, and height, h’ = h + dh. In addition to this, the contour is also allowed to randomly rotate in the z-axis at an angle, α ~ U(−5°, +5°).

As the name suggests, in-plane augmentation essentially simulates the variability in contouring within the axial plane (i.e., variations within X and Y dimensions associated with a slice). Here the prostate contours are allowed to have variations in their latero-lateral or antero-posterior dimensions by a value randomly sampled from a uniform distribution within the interval [−2.7 mm, +2.7 mm]. In addition to this, the contour is randomly allowed to rotate around the z-axis at a small angle, α ~ U(−5°, +5°) (see Figure 2). The choice of intervals for contour variability [−2.7 mm, +2.7 mm] was established by following the results of studies on the inter-observer variability in prostate contouring using MRI [49,50]. These studies report an average standard deviation of 1.1 mm, corresponding to 2.7 mm at a 95% confidence interval.

**Figure 3:**
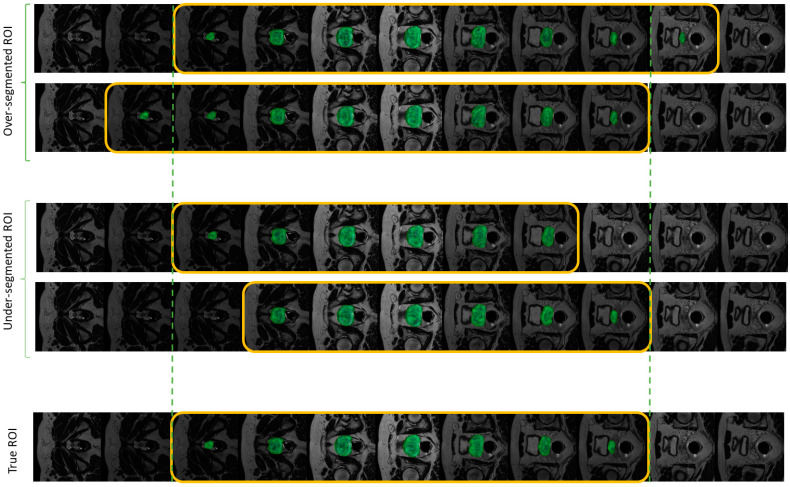
Illustration of various out-plane augmentation scenarios with respect to the true ROI. This augmentation type simulates the variability in the choice of the ROI boundary slice in the craniocaudal direction. The yellow box highlights the slices encompassing the ROI; The vertical dotted green lines indicate the original prostate boundary slices.

Out-plane augmentation essentially represents a scenario where the difference in the segmented ROIs happens due to the difference in the choices of the first and/or last slice in the cranio-caudal direction. In this case, we allowed a maximum shift of one slice on either side related to the choice of the prostate ROI boundary (See Figure 3).

In and out-plane augmentation combines the in- and out-plane augmentations to generate custom contour variations representing real-world scenarios. 

Furthermore, for in-plane augmentation, we considered two possible biases to model intra- and inter-observer variability in contouring: (a) random bias, where the contour associated with each slice can undergo random transformations independently across the axial dimensions, i.e., for each patient, the height and/or width associated with a contour can independently increase/decrease per slice; (b) systematic bias essentially behaves at random but restricts the direction of the variability to remain the same for all the slices associated with a patient. (i.e., the height and width associated with a contour can either increase or decrease for all the slices). Systematic bias mimics the behavior of radiologists in a clinical scenario where some are systematically more “abundant” in their segmentation while others are more “restrictive”.

In summary, we considered five simulated scenarios for each MRI sequence: (1) in-plane augmentation with random bias; (2) in-plane augmentation with systematic bias; (3) out-plane augmentation; (4) in and out-plane augmentation with random bias; (5) in and out-plane augmentation with systematic bias.

### 2.3. Image Processing Pipeline

In this section, we will summarize some of the preprocessing tasks we have adopted before feeding the image-segmentation pair to the feature extraction pipeline of Pyradiomics [51]. 

To standardize the voxel size across all the image acquisitions, we resampled the image dimensions to have a common isotropic voxel size of 2 × 2 × 2 mm^3^. It is important to emphasize that we resampled the in-plane dimensions using third-order B-Spline (cubic) while we resampled the out-plane dimension using nearest neighbor interpolation. We adopted such a strategy to avoid noisy artifacts when upsampling low-resolution images. Subsequently, we used nearest neighbor interpolation to resample all the binary segmentations. 

The intensity values in T2w and SUB sequences are relative and are not directly comparable across patients. To this account, we normalized the intensity values using the mean and standard deviation computed on each patient’s three-dimensional ROI (i.e., whole prostate). We adopted a similar strategy for ADC. However, since ADC intensities have a global meaning, we computed the mean and standard deviation (σ) across all the patients in the internal dataset rather than normalizing them individually. We then clipped the normalized images at 3σ to further reduce the impact of noises. Finally, we shifted the image mean to a value of 300 with a standard deviation of 100. Assuming a normal distribution, such scaling and shifting ensure that most values lie within the range of 0 to 600, minimizing the influence of negative values on the calculation of radiomic features, which is preferred [26,52,53].

### 2.4. Radiomics Feature Extraction Pipeline

We used the default settings of Pyradiomics configuration parameters for feature extraction. A notable difference is in the normalization strategy described in the image processing pipeline. The bin-width parameter was also set to 5, such that the number of bins after discretization lies within the range of 30 to 130 (i.e., for the range 0–600, bin-count = 600/5 = 120), which is shown as having good performance and reproducibility in the literature [54]. Moreover, we believe that a smaller bin width will capture fine-grained information within the whole prostate volume, especially since evidence suggests that only 50% of the patients enrolled in active surveillance have MR visible lesions [17,18,19]. 

One thousand two hundred twenty-four features were extracted from the 3D prostate ROI, pertaining to two main feature families and 17 unfiltered/filtered strategies. Table S1 of the Supplementary Materials reports the complete list of all the features considered. For details on their definition, refer to Pyradiomics documentation [51].

The main feature families constitute:

1. First-order statistics (FO, n = 18) providing information about the histogram of the grey values inside the prostate ROI; and
2. Texture features, providing information about the spatial distribution of grey values. We used the following textural matrices to compute the textural features: Gray Level Co-occurrence Matrix (GLCM, n = 22 features); Gray Level Run Length Matrix (GLRLM, n = 16 features); Gray Level Size Zone Matrix (GLSZM, n = 16 features).

We utilized all the standard filtering techniques offered by the Pyradiomics package, including LoG (Laplacian of Gaussian filter with kernel sizes, σ = 2, 3, 4, 5 mm), wavelet (eight decompositions per level based on either applying high (H) or low (L) pass filter along each of the three dimensions—HHH, HHL, HLH, HLL, LHH, LHL, LLH, LLL), squared, square root, logarithm, and exponential filters as part of feature extraction pipeline.

### 2.5. Stability Analysis

We assessed the stability of radiomic features using the intraclass correlation coefficient form—ICC(1,1) [35] (i.e., model = one-way random effects, type = single rater, and definition = absolute agreement). The model was chosen as one-way random effects since each patient is subjected to random segmentations generated by the augmentation model representing a randomly chosen sample of possible annotators (or raters). The measurement from each rater (i.e., each simulated segmentation) will be the basis of the actual measurement (i.e., the extracted feature); hence the ICC type = single rater. The definition = absolute agreement since we expect the computed feature to remain the same for the same subject across the different annotators. We calculated the ICC(1,1) using the Python library Pingouin (v 0.3.12) [55]. 

ICC estimate ranges between 0 and 1, with values closer to 1 showing the highest stability. Conventionally to identify stable radiomic features, the ICC estimate is thresholded [35]. In this study, we followed a similar strategy where we categorized a radiomics feature as stable if the lower bound on the 95% confidence interval of the ICC estimate was above 0.90. 

**Figure 4:**
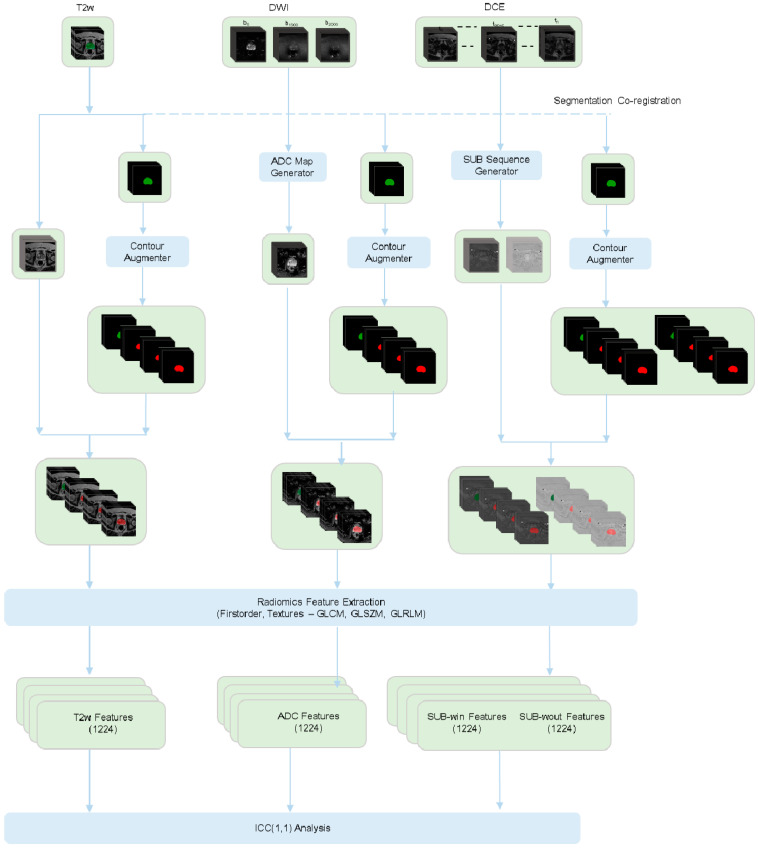
Schematic representation of the workflow involved in the stability study on prostate radiomic features extracted from T2w, DWI, and DCE sequences to variations in segmentation. Manual prostate annotation provided for the T2w sequence was co-registered with the other sequences. The segmentations were then augmented to generate 15 synthetic contours (in the figure as an example, 3 synthetic contours are generated + the original segmentation). A total of 1224 radiomic features were extracted from each of the image-mask pairs. The stability of the features was analyzed using ICC (1,1).

We used an external dataset to assess the robustness of stable features identified in the internal population by considering the overlap between stable features across the two datasets. We labeled an overlapped feature as robust if the threshold criterium is satisfied in both datasets, i.e., if the minimum of the lower bound of the ICC estimate in the internal and external datasets is above 0.90. Figure 4 illustrates the overall workflow followed in this study. The Python-based implementation is provided as open-source and is available at https://github.com/sithin-int/stability_study.git (accessed on 19 July 2023) to promote further investigation and reproducibility.

## 3. Results

In this study, we investigated the impact of variations in segmentation on the stability of radiomic features using an in silico contour variability simulator covering three augmentations scenarios—in-plane vs. out-plane vs. in and out-plane; and two segmentation biases—random vs. systematic. Table 2 summarizes the distribution of pairwise dice scores between the ground truth (manual segmentation by the experienced radiologist) and generated contour across all the augmentation-bias configurations. Table 3 presents a simplified summary of the percentage of stable and robust features across all these configurations. Among them, in and out-plane systematic variations significantly impacted radiomic features’ stability, while out-plane variations seem to affect the least. We observed that the variability margin also depends on the sequence and the filtering strategy.

The sheer amount of output data generated by our analysis makes it challenging to discuss each configuration in detail. To simplify, the remainder of the paper will focus exclusively on the most clinically relevant configuration, i.e., in and out plane augmentation with systematic bias. For all the other scenarios, we recommend referring to the supplementary materials. Another overhead may be attributed to the 16 distinct filtering strategies investigated in our study. To streamline our analysis, we only considered filter(s) that showed stability for a feature in the internal population, referred to as best-filter(s), to be compared with the external population for robustness evaluation. The terms “stability” and “robustness” used in this section need to be carefully interpreted. Stable features refer to radiomic features that are stable to variations in segmentation exclusively based on their behavior on the internal population. Robust features, on the other hand, are the overlapped stable features in both the internal and external populations.

Among unfiltered/original radiomic features subjected to in and out-plane systematic variations, T2w (stability = 69%) and SUBwin (65%) sequences showed high stability, followed by SUBwout (53%) and ADC (47%). On the contrary, during robustness evaluation, the fraction of stable features dropped by a significant margin for T2w (drop margin~30%) and SUBwin (~20) while it remained within the range of 10% for SUBwout, and ADC. Consequently, the robustness of features proceeds in the order of SUBwin (robust = 46%), SUBwout (43%), ADC (36%), and T2w (36%). Although T2w sequence features exhibited high stability, they showed the least robustness among all the sequences. 

Filtering, on the other hand, improved radiomic features’ stability considerably compared to the unfiltered counterpart. All the filtered sequences had a mean improvement margin of 38%. ADC and T2w features showed a stability of 97%, followed by SUBwin (96%) and SUBwout (94%). The robustness assessment also indicated a high overlap among stable features between the internal and external datasets. The T2w sequence showed the least robustness yet had almost 81% of robust filtered features. This is an improvement of nearly 50% compared to its behavior in the unfiltered configuration. ADC (robustness = 94%) exhibited a similar trend with almost 60% of improvement margin. For both SUBwin and SUBwout sequences, 93% of all the stable features were robust.

**Figure 5:**
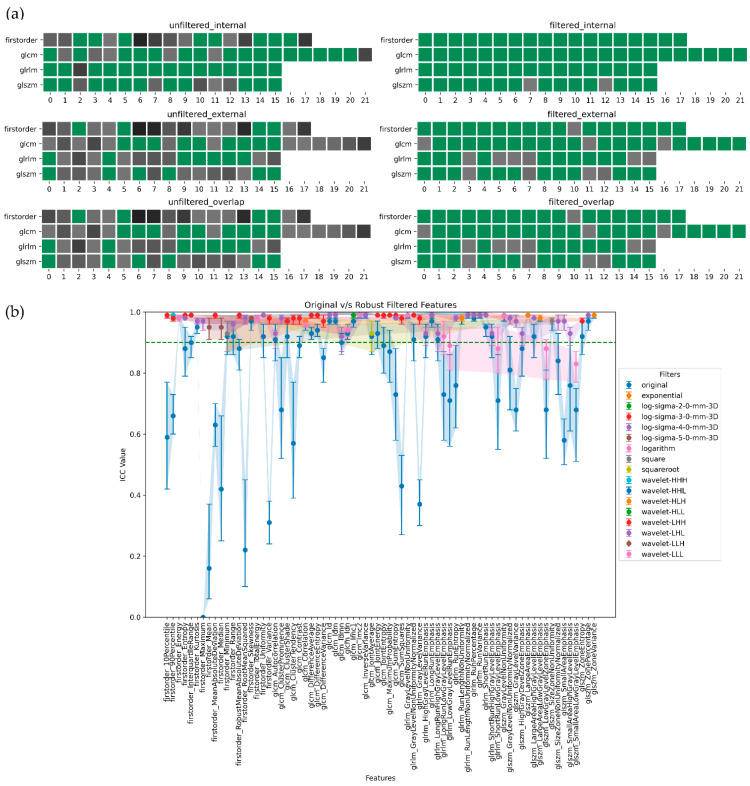
Stability and robustness of filtered/unfiltered T2w radiomics features subjected to in and out-plane-systematic segmentation variations. A feature is considered stable if the lower bound of the 95% CI of the ICC estimate > 0.90. (**a**) Stable v/s unstable feature heatmap—grey cells indicate unstable features, with darker shades of grey indicating lower ICC bounds. All the green cells represent stable features. (**b**) ICC plot portrays the overlap computed as the minimum stability value of a feature in the internal and external dataset grouped by both unfiltered and best-filtered configurations. For simplicity, we are only displaying the best filter(s) that yielded the highest ICC lower bound after overlap. The dotted green line indicates the stability threshold.

**Figure 6:**
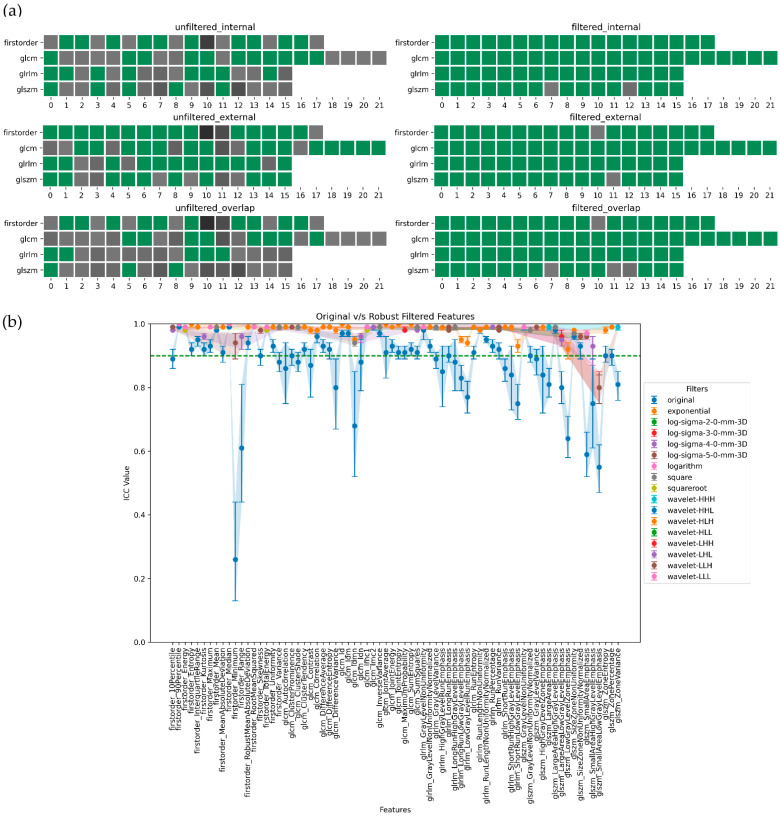
Stability and robustness of filtered/unfiltered ADC radiomics features subjected to in and out-plane-systematic segmentation variations. A feature is considered stable if the lower bound of the 95% CI of the ICC estimate > 0.90. (**a**) Stable v/s unstable feature heatmap—grey cells indicate unstable features, with darker shades of grey indicating lower ICC bounds. All the green cells represent stable features. (**b**) ICC plot portrays the overlap computed as the minimum stability value of a feature in the internal and external dataset grouped by both unfiltered and best-filtered configurations. For simplicity, we are only displaying the best filter(s) that yielded the highest ICC lower bound after overlap. The dotted green line indicates the stability threshold.

**Figure 7:**
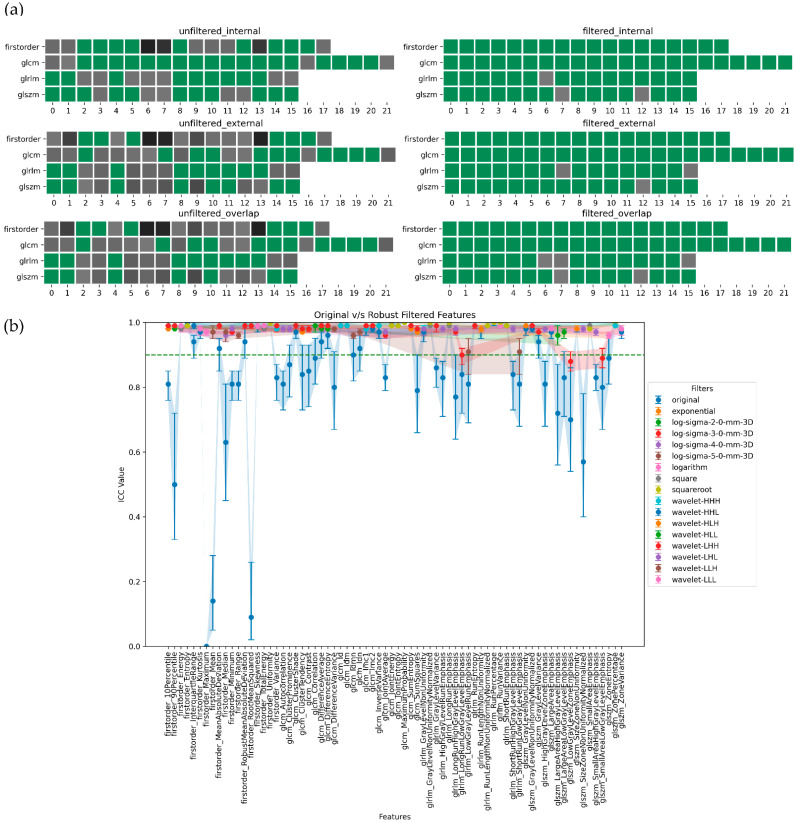
Stability and robustness of filtered/unfiltered SUBwin radiomics features subjected to in and out-plane-systematic segmentation variations. A feature is considered stable if the lower bound of the 95% CI of the ICC estimate > 0.90. (**a**) Stable v/s unstable feature heatmap—grey cells indicate unstable features, with darker shades of grey indicating lower ICC bounds. All the green cells represent stable features. (**b**) ICC plot portrays the overlap computed as the minimum stability value of a feature in the internal and external dataset grouped by both unfiltered and best-filtered configurations. For simplicity, we are only displaying the best filter(s) that yielded the highest ICC lower bound after overlap. The dotted green line indicates the stability threshold.

**Figure 8:**
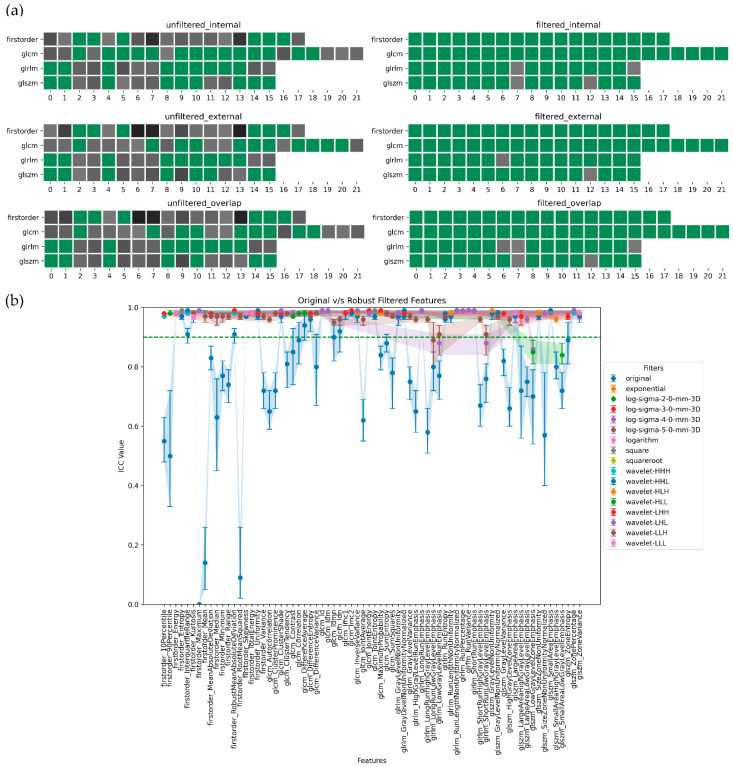
Stability and robustness of filtered/unfiltered SUBwout radiomics features subjected to in and out-plane-systematic segmentation variations. A feature is considered stable if the lower bound of the 95% CI of the ICC estimate > 0.90. (**a**) Stable v/s unstable feature heatmap—grey cells indicate unstable features, with darker shades of grey indicating lower ICC bounds. All the green cells represent stable features. (**b**) ICC plot portrays the overlap computed as the minimum stability value of a feature in the internal and external dataset grouped by both unfiltered and best-filtered configurations. For simplicity, we are only displaying the best filter(s) that yielded the highest ICC lower bound after overlap. The dotted green line indicates the stability threshold.

In summary, ADC-filtered features demonstrated the highest degree of robustness, followed by SUB and T2w. Figure 5, Figure 6, Figure 7 and Figure 8, in their panels (a), highlight the impact of the unfiltered v/s filtering strategy on the stability and, consequently, on the robustness of radiomic features as a heatmap. Figure 5, Figure 6, Figure 7 and Figure 8, in their panels (b), portray the overlap among the ICC estimates between the internal and external population for unfiltered and best-filtered feature configurations.

## 4. Discussion

Reliability (or stability) is essential to use quantitative image-based features as potential biomarkers for clinical applications. While numerous factors could influence radiomics feature repeatability, this study focused exclusively on the stability of features to variations in segmentation. This was accomplished by designing an in silico contour generator that simulates variations commonly observed among manual annotators. This study investigated five distinct configurations covering three augmentation scenarios—in-plane, out-plane, and in and out-plane—and two segmentation biases—random and systematic. The generator’s design was inspired by the data augmentation paradigm in DL and utilized bounded affine transformations. 

In the premise of prostate mpMRI analysis, only a few studies have investigated the stability of features to variations in segmentation. For example, Xu, Lili, et al. [33] obtained lesion annotations from two radiologists to assess the feature stability to develop a robust radiomics model for predicting extraprostatic extension. Another study undertaken by Sushentsev, Nikita, et al. [10] used morphological perturbations such as opening and/or closing on the lesion ROI to simulate contour variations without involving manual annotators. The robust features they identified were then used to predict PCa progression. It is important to note that, in both studies, the lesions were used as the ROIs. This may not be ideal in active surveillance, especially since only half the patients will likely show MR-visible lesions [17,18,19]. To this account, we recommend using the whole prostate gland to analyze MRI images from very low-risk PCa patients on active surveillance.

**Figure 9:**
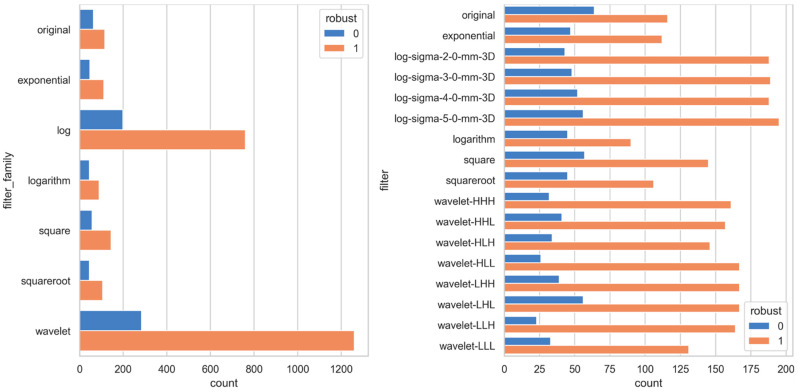
The histogram plot presents a summary of the frequency at which a robust feature is associated with a particular filter/filter-family subjected to systematic-in and out-plane augmentation across all image sequences.

Segmentation of the prostate gland is challenging due to the lack of a clear visual boundary and significant variations in its size and shape among patients. These differences lead to intra-/inter-observer segmentation variations among human annotators. Our results highlight that these variations can notably impact stability, particularly among unfiltered radiomic features. However, it can be mitigated by incorporating filtering strategies. While the Image Biomarker Standardisation Initiative (ISBI) does not address pre-filtering strategies, filters such as Wavelet and LoG have been shown to yield highly predictive signatures [9,56]. Our results suggest that the use of Wavelet and LoG filters could also lead to considerable improvements in terms of stability (see Figure 9).

Radiomics mpMRI studies rarely include DCE sequences but rely primarily on bi-parametric (bp) or uni-parametric MR sequences. This may be attributed to two major reasons: (i) despite the loss in diagnostic information [20], bpMRI is recommended for biopsy naïve PCa patients and is more suitable for large studies [57] as it eliminates the risk of adverse effects due to contrast agent and speeds up image acquisition; (ii) the processing pipeline associated with DCE is complicated as we need to consider the temporal domain. Conventionally pharmacokinetic maps are extracted from DCE images and are used for radiomics analysis [58]. 

PCa patients on active surveillance do not fall into the category of biopsy naïve patients; hence, we chose to include DCE sequence in our analysis. Instead of computing pharmacokinetic maps, we derived subtraction maps to encapsulate the contrast agent’s wash-in and wash-out phases. Schwier, Michael, et al. [26] conducted a test-retest feature repeatability study on prostate mpMRI sequences, examining SUB maps. They reported that none of the radiomic features extracted from the whole-prostate gland was stable. Our findings suggest the contrary, where SUB sequence features showed high robustness among unfiltered and filtered configurations. However, it is essential to note that these results are not directly comparable. Their study focused on the stability of SUB features in a test-retest configuration, while we investigated feature stability subjected to variations in segmentation. Nonetheless, we would like to emphasize this point to promote further investigation of SUB maps.

A conventional approach to evaluating the stability of radiomic features involves using a test-retest paradigm [53,59,60]. It is often the case that test-retest acquisitions may be difficult to obtain or not readily available. Zwanenburg et al. [61] proposed a solution to this approach by leveraging the data augmentation paradigm in DL. They synthesized different image acquisitions by considering linear/non-linear perturbations of the original image. They used a combination of transformations such as translation, rotation, volume growth/shrinkage, super-voxel-based contour randomization, and noise addition. The in silico contour segmentation tool proposed in this study also follows a similar pipeline. Yet, instead of augmenting the images, we aim to induce variations in the segmentation mask. Moreover, we restricted augmentations to replicate real-world variations commonly seen among manual annotators. The potential application of this tool lies in promoting future stability studies to segmentation as it can be easily integrated into any radiomics pipeline. 

The limitations of this study highlight possible future research directions. We assumed we could simulate the segmentation variations resulting from inter/intra-observer variability by subjecting ground truth annotation to bounded affine transformations. Nevertheless, it is uncertain how much this approximation reflects the variations observed in the real world. Therefore, further investigation is warranted to validate the proposed method by comparing it with the conventional clinical inter/intra-observer variations study involving manual annotators. This increases the overhead of involving multiple radiologists, which was not feasible in this study. Although we considered the overlap in terms of stability between the internal and external datasets, it is essential to emphasize that these populations may not be directly comparable. This is particularly true for SUB maps. For the external population, SUB maps were scanner derived considering the early post-contrast and pre-contrast. On the other hand, SUB maps were derived manually for the internal population by considering the contrast agent’s wash-in and wash-out phases. Yet another limitation may be attributed to the scope of this study, where the analysis is limited to the stability of prostate radiomic features subjected to variations in segmentation. In reality, numerous other sources of variations affect feature stability, such as the heterogeneity of study protocols, scan acquisition parameters, reconstruction settings, and feature extraction pipeline, which need to be considered to improve the overall generalizability of radiomic signatures.

## 5. Conclusions

This study presents a method to evaluate the stability of radiomic features to variations in segmentation. The technique was then employed to examine the stability of mpMRI radiomic features extracted from the whole prostate gland among PCa patients in active surveillance. Our findings highlight that unfiltered radiomic features are susceptible to variations in segmentation. However, by incorporating pre-filtering strategies, the feature stability improved. We also recommend using external datasets to validate the robustness of stable features identified on the internal dataset.

The contour augmentation method proposed in this study also has the potential to enhance the robustness of PI-RADS [7] determination, i.e., by evaluating PI-RADS using multiple augmented contours, a distribution of scores can be obtained mirroring the uncertainties associated with the single definition of the regions of interest.

## Figures and Tables

**Table 1:** A simplified summary of the specifications for both the internal and external datasets used in this study.

Specifications	(a) Internal Dataset	(b) External Dataset
No. of Patients	100	15
Manufacturer	Ingenia (Philips Medical System, Best, The Netherlands)	GE Signa HDxt platform and GE Discovery MR750w (General Electric Healthcare, Milwaukee, WI) machines.
Magnetic Field Strength	1.5 T	3.0 T
Endorectal Coil	Yes	Yes
PIRADSv2 Compliant	Yes	Yes
Acquisition Protocol	T2w (TR/TE = 4910/110 ms, slice thickness = 3 mm, pixel spacing = 0.297 mm); DWI (b-values = 0, 1500 and 2000 s/mm^2^, TR/TE = 3320/106 ms, slice thickness = 3 mm, pixel spacing = 1.250 mm); DCE (TR/TE = 4.03/1.88 ms, slice thickness = 3 mm, pixel spacing = 1.136 mm, acquired with high temporal resolution < 10 s).	T2w (TR/TE = 3350–5109/84–107 ms, slice thickness = 3 mm, pixel spacing = 0.273–0.312 mm); DWI (b-values of 0 and 1400 s/mm^2^, TR/TE = 2500–8150/76.7–80.6 ms, slice thickness = 3–4 mm, pixel spacing = 0.625–0.703 mm); DCE (TR/TE = 3.68–4.1/1.3–1.42 ms, slice thickness = 5–6 mm, pixel spacing = 0.547–1.015 mm).
GT Segmentation	Whole prostate gland segmentation on T2w	Whole prostate gland segmentation on T2w, ADC, and SUB

**Table 2:** The mean and standard deviation of the dice distribution for each sequence subjected to five different augmentation scenarios.

**(a) Internal**								
**aug config**	**T2w**		**ADC**		**SUB_win_**		**SUB_wout_**	
	**mean**	**std**	**mean**	**std**	**mean**	**std**	**mean**	**std**
InP-R	0.95	0.01	0.95	0.01	0.95	0.01	0.95	0.01
InP-S	0.95	0.02	0.95	0.02	0.95	0.02	0.95	0.02
OutP	0.99	0.01	0.99	0.01	0.99	0.01	0.99	0.01
In&OutP-R	0.95	0.01	0.95	0.01	0.95	0.02	0.95	0.01
In&OutP-S	0.94	0.03	0.95	0.03	0.94	0.02	0.94	0.03
**(b) External**								
**aug config**	**T2w**		**ADC**		**SUB**			
	**mean**	**std**	**mean**	**std**	**mean**	**std**		
InP-R	0.95	0.01	0.96	0.01	0.95	0.02		
InP-S	0.95	0.03	0.95	0.02	0.95	0.03		
OutP	0.99	0.01	0.99	0.01	0.99	0.01		
In&OutP-R	0.94	0.02	0.95	0.01	0.94	0.02		
In&OutP-S	0.94	0.03	0.95	0.03	0.94	0.03		

aug config—augmentation configuration; InP-R/S—InPlane Random/Systematic; OutP—OutPlane; In&OutP-R/S—In and Out Plane Random/Systematic.

**Table 3:** A summary of the fraction of stable and robust features associated with the mpMRI sequences considered in the study. A feature is considered as stable if the lower bound of the 95% CI of the ICC estimate > 0.90. A stable feature will be categorized as robust if the feature remains stable in both the internal and external dataset.

**(a) T2w**																				
**aug config**	**firstorder**				**glcm**				**glrlm**				**glszm**				**Overall**			
	**O**		**BF**		**O**		**BF**		**O**		**BF**		**O**		**BF**		**O**		**BF**	
	**S**	**R**	**S**	**R**	**S**	**R**	**S**	**R**	**S**	**R**	**S**	**R**	**S**	**R**	**S**	**R**	**S**	**R**	**S**	**R**
InP-R	0.72	0.72	1	1	0.95	0.86	1	1	0.94	0.75	1	1	0.81	0.62	1	0.88	0.86	0.75	1	0.97
InP-S	0.44	0.22	1	0.94	0.73	0.41	1	0.86	0.94	0.44	1	0.62	0.75	0.44	1	0.81	0.71	0.38	1	0.82
OutP	0.83	0.83	1	1	1	1	1	1	1	1	1	1	0.94	0.81	1	1	0.94	0.92	1	1
In&OutP-R	0.72	0.61	1	1	0.95	0.82	1	1	0.94	0.56	1	1	0.81	0.56	1	0.88	0.86	0.65	1	0.97
In&OutP-S	0.44	0.22	1	0.94	0.73	0.41	1	0.86	0.94	0.44	1	0.62	0.69	0.38	0.88	0.75	0.69	0.36	0.97	0.81
**(b) ADC**																				
**aug config**	**firstorder**				**glcm**				**glrlm**				**glszm**				**Overall**			
	**O**		**BF**		**O**		**BF**		**O**		**BF**		**O**		**BF**		**O**		**BF**	
	**S**	**R**	**S**	**R**	**S**	**R**	**S**	**R**	**S**	**R**	**S**	**R**	**S**	**R**	**S**	**R**	**S**	**R**	**S**	**R**
InP-R	0.94	0.89	1	1	1	0.91	1	1	1	0.94	1	1	0.81	0.69	0.94	0.94	0.94	0.86	0.99	0.99
InP-S	0.5	0.5	1	1	0.55	0.41	1	1	0.69	0.5	1	1	0.44	0.31	0.88	0.88	0.54	0.43	0.97	0.97
OutP	1	0.89	1	1	1	0.95	1	1	0.94	0.94	1	1	0.81	0.81	1	1	0.94	0.9	1	1
In&OutP-R	0.94	0.89	1	0.94	1	0.91	1	1	0.94	0.88	1	1	0.75	0.56	0.94	0.94	0.92	0.82	0.99	0.97
In&OutP-S	0.56	0.56	1	0.94	0.5	0.36	1	1	0.5	0.31	1	1	0.31	0.19	0.88	0.81	0.47	0.36	0.97	0.94
**(c) SUB_win_**																				
**aug config**	**firstorder**				**glcm**				**glrlm**				**glszm**				**Overall**			
	**O**		**BF**		**O**		**BF**		**O**		**BF**		**O**		**BF**		**O**		**BF**	
	**S**	**R**	**S**	**R**	**S**	**R**	**S**	**R**	**S**	**R**	**S**	**R**	**S**	**R**	**S**	**R**	**S**	**R**	**S**	**R**
InP-R	0.72	0.67	1	1	0.91	0.91	1	1	0.81	0.62	1	1	0.81	0.56	1	0.88	0.82	0.71	1	0.97
InP-S	0.5	0.33	1	1	0.82	0.5	1	1	0.56	0.56	0.94	0.81	0.75	0.56	0.88	0.88	0.67	0.49	0.96	0.93
OutP	0.83	0.78	1	1	1	1	1	1	1	1	1	1	1	0.88	1	1	0.96	0.92	1	1
In&OutP-R	0.72	0.61	1	1	0.91	0.82	1	1	0.81	0.62	1	0.88	0.81	0.5	1	0.88	0.82	0.65	1	0.94
In&OutP-S	0.5	0.33	1	1	0.82	0.5	1	1	0.56	0.56	0.94	0.81	0.69	0.44	0.88	0.88	0.65	0.46	0.96	0.93
**(d) SUB_wout_**																				
**aug config**	**firstorder**				**glcm**				**glrlm**				**glszm**				**Overall**			
	**O**		**BF**		**O**		**BF**		**O**		**BF**		**O**		**BF**		**O**		**BF**	
	**S**	**R**	**S**	**R**	**S**	**R**	**S**	**R**	**S**	**R**	**S**	**R**	**S**	**R**	**S**	**R**	**S**	**R**	**S**	**R**
InP-R	0.78	0.67	1	1	0.95	0.91	1	1	0.94	0.62	1	1	0.88	0.62	0.94	0.88	0.89	0.72	0.99	0.97
InP-S	0.33	0.33	1	1	0.68	0.41	1	1	0.56	0.56	1	0.81	0.56	0.5	0.94	0.88	0.54	0.44	0.99	0.93
OutP	0.89	0.78	1	1	1	1	1	1	0.94	0.94	1	1	1	0.88	1	1	0.96	0.9	1	1
In&OutP-R	0.72	0.61	1	1	0.91	0.82	1	1	0.81	0.62	1	0.88	0.81	0.56	0.88	0.88	0.82	0.67	0.97	0.94
In&OutP-S	0.33	0.33	1	1	0.64	0.41	1	1	0.56	0.56	0.88	0.81	0.56	0.44	0.88	0.88	0.53	0.43	0.94	0.93

aug config—augmentation configuration; InP-R/S—InPlane Random/Systematic; OutP—OutPlane; In&OutP-R/S—In and Out Plane Random/Systematic; O—Original/Raw/Unfiltered; BF—Best Filtered; S—Stable; R—Robust.

## Data Availability

Raw data were generated at Fondazione IRCCS Istituto Nazionale dei Tumori. Derived data supporting the findings of this study are available from the corresponding authors [T.R. and C.T.] on request.

## Supplementary Materials

The following supporting information can be downloaded at: https://www.mdpi.com/article/10.3390/jpm13071172/s1. Table S1: The complete list of radiomics features and filtering strategies considered in the study. Folder B.1 ICC Plots: The folder named “plots” contains all the figures associated with each sequence and augmentation scenario for both internal and external population. Eg. To visualize the ICC plot associated with internal T2w sequence subjected to in-plane-random augmentation; Open plots->t2w->in_plane_random_internal.png. Folder B.2 Overlap Plots: The folder named “overlap_plots” contains all the figures associated with each sequence and augmentation scenario. The overlap here indicates the overlap (or minimum) of ICC estimates between both the internal and external population. Eg. To visualize the overlap plot associated with T2w sequence subjected to in-plane-random augmentation; Open overlap_plots->t2w->in_plane_random.png Folder B.3 Heatmaps: The folder named “heatmaps” contains all the heatmaps associated with each sequence and augmentation scenario. This is another representation of overlapping plots highlighting robust features. Robust features are highlighted green while unstable features are highlighted gray. Eg. To visualize the heatmap associated with T2w sequence subjected to in-plane-random augmentation; Open heatmaps->t2w->in_plan_random.png. Folder B.4 Robust Filter Histograms: The folder named “histplots” contains all the figures associated with each augmentation scenario. This plot counts the number of times a filter/filter family was found among robust (1) non-robust (0) features. Eg: To visualize the histogram associated with in-plane-random augmentation scenario; Open histplots->in_plane_random.png. Folder Robust Features: All the robust features associated with each augmentation configuration are contained in folder “robust_features”. The filtering technique available in MS excel can guide the user to extract information necessary for a specific sequence.
