# Supplementary material for: Stability of Multi-Parametric Prostate MRI Radiomic Features to Variations in Segmentation
Source: J Pers Med. 2023 Jul 22;13(7):1172. doi: 10.3390/jpm13071172 (PMC10381192; doi:10.3390/jpm13071172)
Supplement: Supplementary file 1 [file jpm-13-01172-s001.zip › Supplementary Material.pdf]

Supplementary Materials

Table S1. The complete list of features and filtering strategies considered in the study.

Feature Family	Feature	Filter
firstorder	10Percentile, 90Percentile, Energy, Entropy, InterquartileRange, Kurtosis, Maximum, Mean, MeanAbsoluteDeviation, Median, Minimum, Range, RobustMeanAbsoluteDeviation, RootMeanSquared, Skewness, TotalEnergy, Uniformity, Variance	Unfiltered (original), Exponential, LoG-sigma-2-0-mm-3D, LoG-sigma-3-0-mm-3D, LoG-sigma-4-0-mm-3D, LoG-sigma-5-0-mm-3D, Logarithm, Square, Squareroot, Wavelet-HHH, Wavelet-HHL, Wavelet-HLH, Wavelet-HLL, Wavelet-LHH, Wavelet-LHL, Wavelet-LLH, Wavelet-LLL
Texture (glcm)	Autocorrelation, ClusterProminence, ClusterShade, ClusterTendency, Contrast, Correlation, DifferenceAverage, DifferenceEntropy, DifferenceVariance, Id, Idm, Idmn, Idn, Imc1, Imc2, InverseVariance, JointAverage, JointEnergy, JointEntropy, MaximumProbability, SumEntropy, SumSquares	
Texture (glrlm)	GrayLevelNonUniformity, GrayLevelNonUniformityNormalized, GrayLevelVariance, HighGrayLevelRunEmphasis, LongRunEmphasis, LongRunHighGrayLevelEmphasis, LongRunLowGrayLevelEmphasis, LowGrayLevelRunEmphasis, RunEntropy, RunLengthNonUniformity, RunLengthNonUniformityNormalized, RunPercentage, RunVariance, ShortRunEmphasis, ShortRunHighGrayLevelEmphasis, ShortRunLowGrayLevelEmphasis	
Texture (glszm)	GrayLevelNonUniformity, GrayLevelNonUniformityNormalized, GrayLevelVariance, HighGrayLevelZoneEmphasis, LargeAreaEmphasis, LargeAreaHighGrayLevelEmphasis, LargeAreaLowGrayLevelEmphasis, LowGrayLevelZoneEmphasis, SizeZoneNonUniformity, SizeZoneNonUniformityNormalized, SmallAreaEmphasis, SmallAreaHighGrayLevelEmphasis,	

	SmallAreaLowGrayLevelEmphasis, ZoneEntropy, ZonePercentage, ZoneVariance	

Navigating Supplementary Figures

B.1 ICC Plots: The folder named "plots" contains all the figures associated with each sequence and augmentation scenario for both internal and external population. Eg. To visualize the ICC plot associated with internal T2w sequence subjected to in-plane-random augmentation; Open plots->t2w->in_plane_random_internal.png

B.2 Overlap Plots: The folder named "overlap_plots" contains all the figures associated with each sequence and augmentation scenario. The overlap here indicates the overlap (or minimum) of ICC estimates between both the internal and external population. Eg. To visualize the overlap plot associated with T2w sequence subjected to in-plane-random augmentation; Open overlap_plots -> t2w -> in_plane_random.png

B.3 Heatmaps: The folder named "heatmaps" contains all the heatmaps associated with each sequence and augmentation scenario. This is another representation of overlapping plots highlighting robust features. Robust features are highlighted green while unstable features are highlighted gray. Eg. To visualize the heatmap associated with T2w sequence subjected to in-plane-random augmentation; Open heatmaps->t2w->in_plan_random.png

B.4 Robust Filter Histograms: The folder named "histplots" contains all the figures associated with each augmentation scenario. This plot counts the number of times a filter/filter family was found among robust (1) / non-robust (0) features. Eg: To visualize the histogram associated with in-plane-random augmentation scenario; Open histplots->in_plane_random.png

Navigating Supplementary Excel Files: All the robust features associated with each augmentation configuration are contained in folder "robust_features". The filtering technique available in MS excel can guide the user to extract information necessary for a specific sequence.
