# Supplementary figures and images for: Stability of Multi-Parametric Prostate MRI Radiomic Features to Variations in Segmentation
Source: J Pers Med. 2023 Jul 22;13(7):1172. doi: 10.3390/jpm13071172 (PMC10381192; doi:10.3390/jpm13071172)

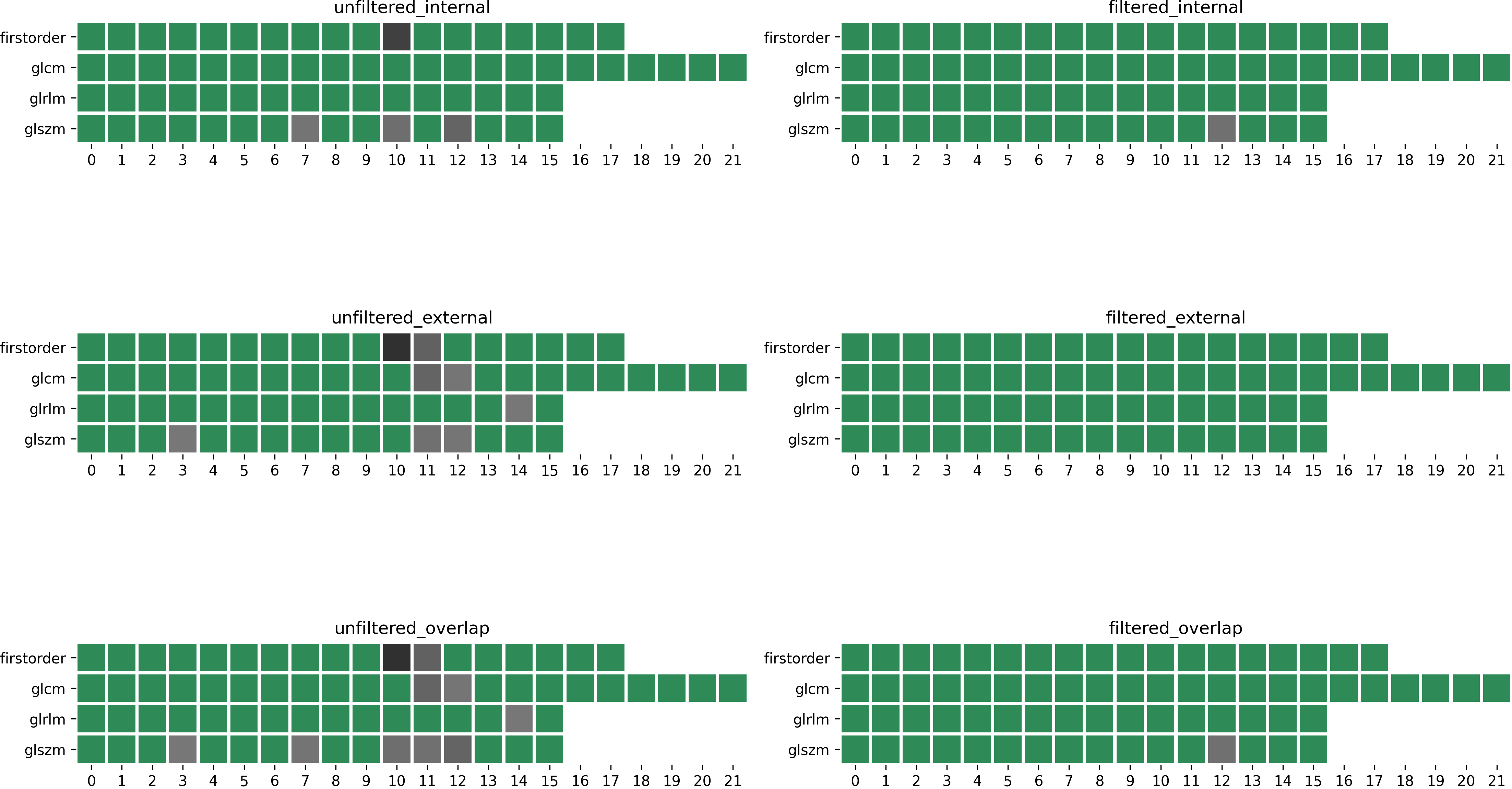

Supplement: Supplementary file 1 [file jpm-13-01172-s001.zip › heatmaps/adc/in_plane_random.png]

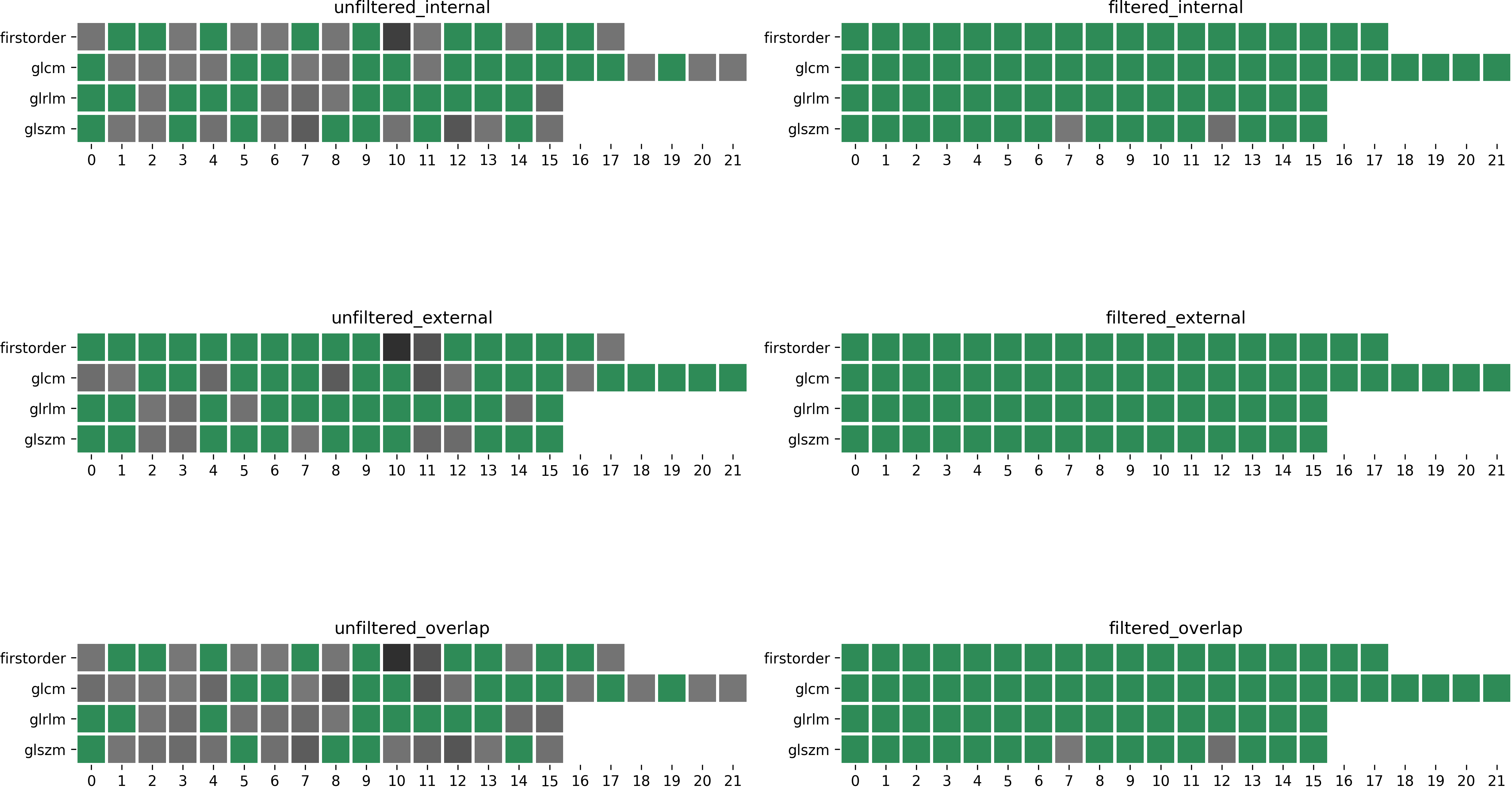

Supplement: Supplementary file 1 [file jpm-13-01172-s001.zip › heatmaps/adc/in_plane_systematic.png]

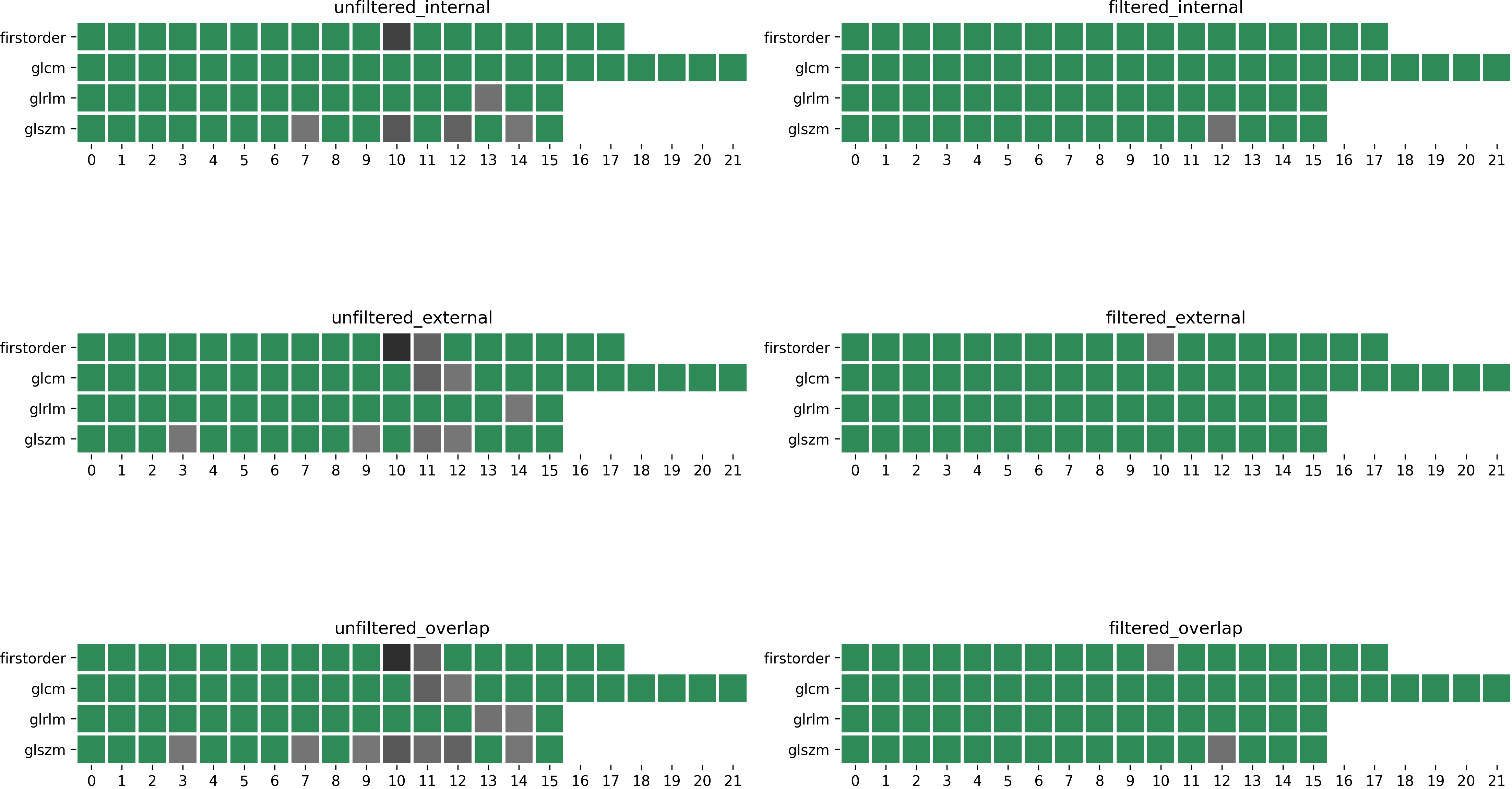

Supplement: Supplementary file 1 [file jpm-13-01172-s001.zip › heatmaps/adc/inout_plane_random.png]

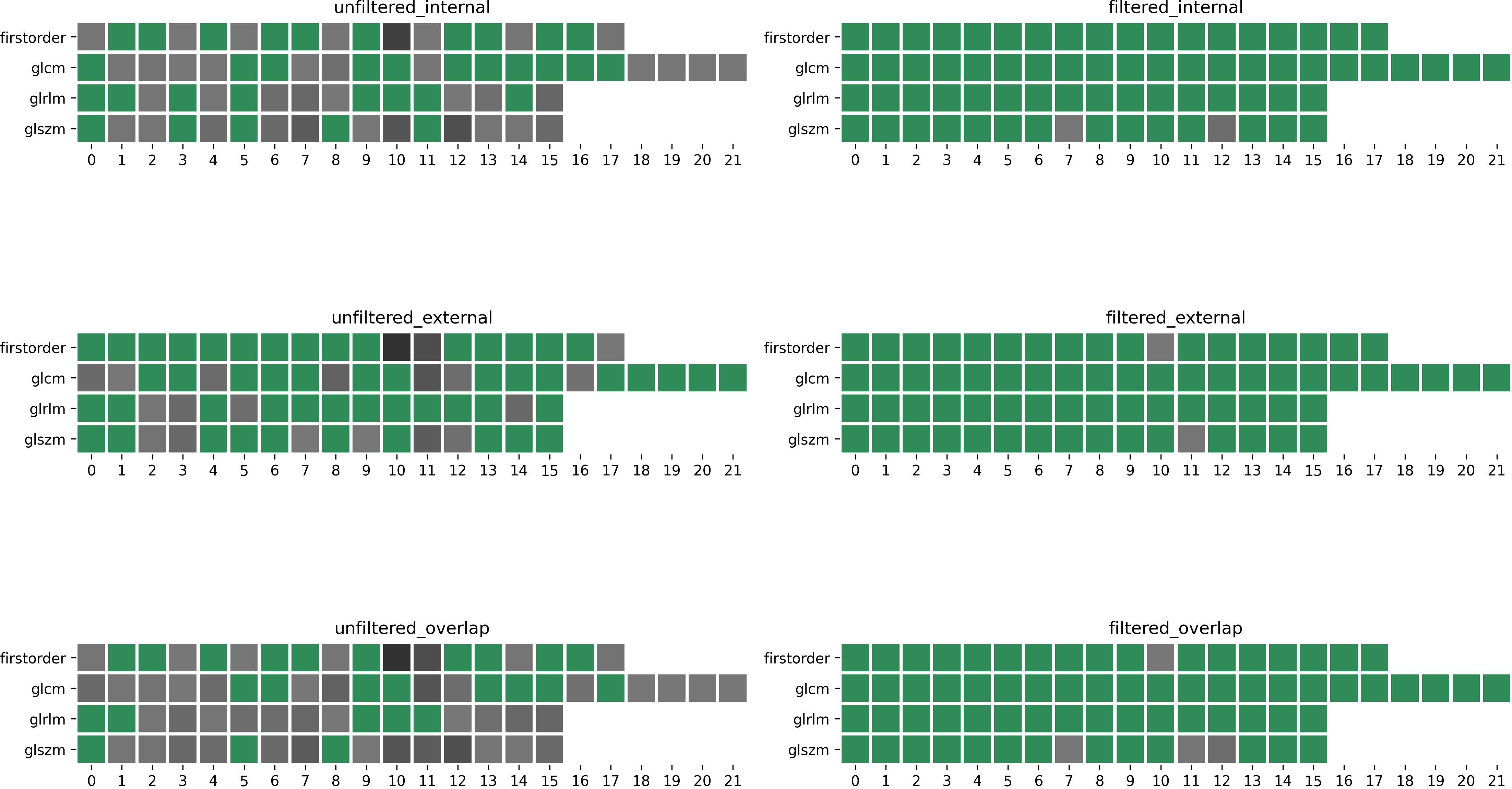

Supplement: Supplementary file 1 [file jpm-13-01172-s001.zip › heatmaps/adc/inout_plane_systematic.png]

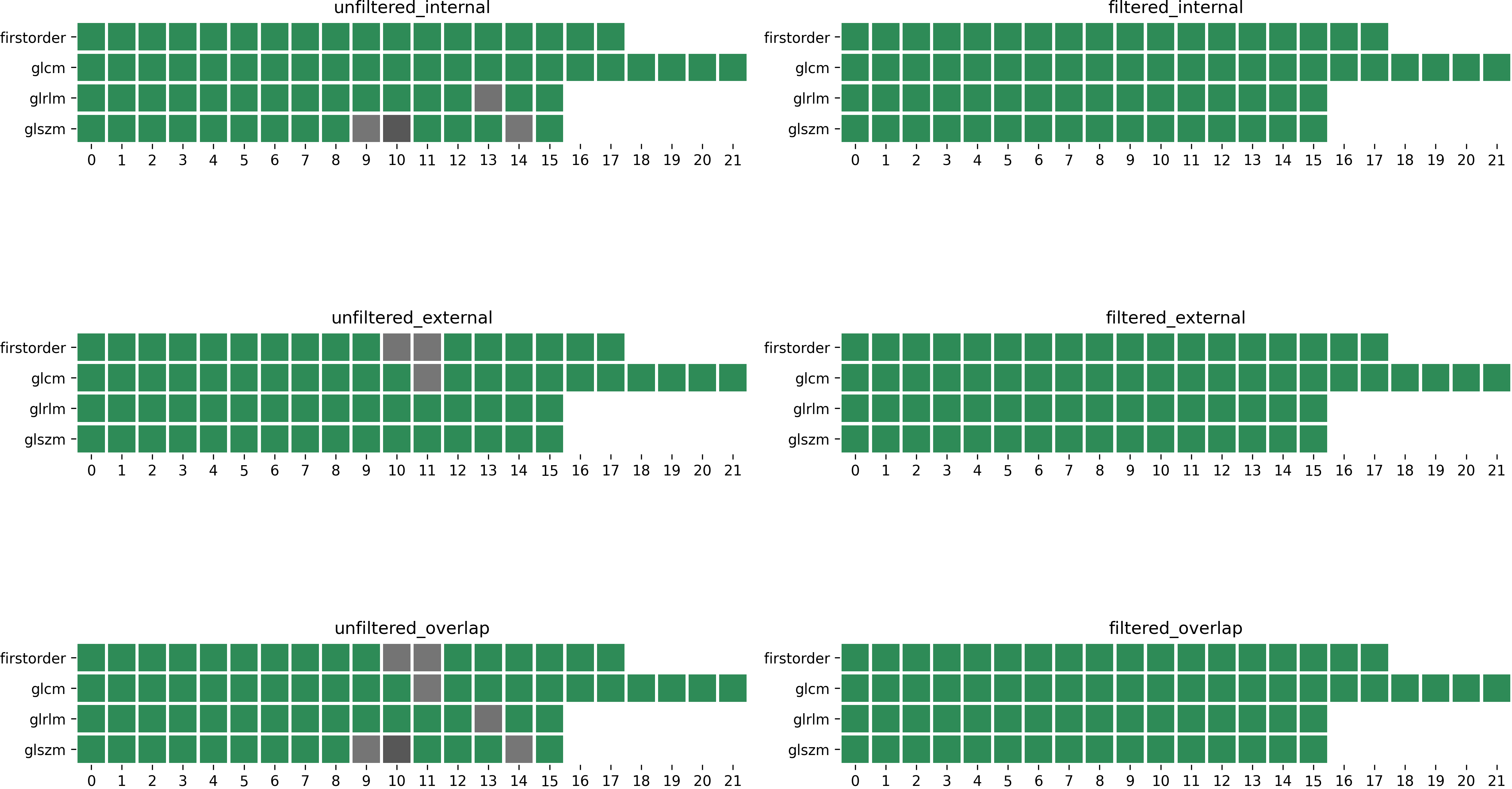

Supplement: Supplementary file 1 [file jpm-13-01172-s001.zip › heatmaps/adc/out_plane.png]

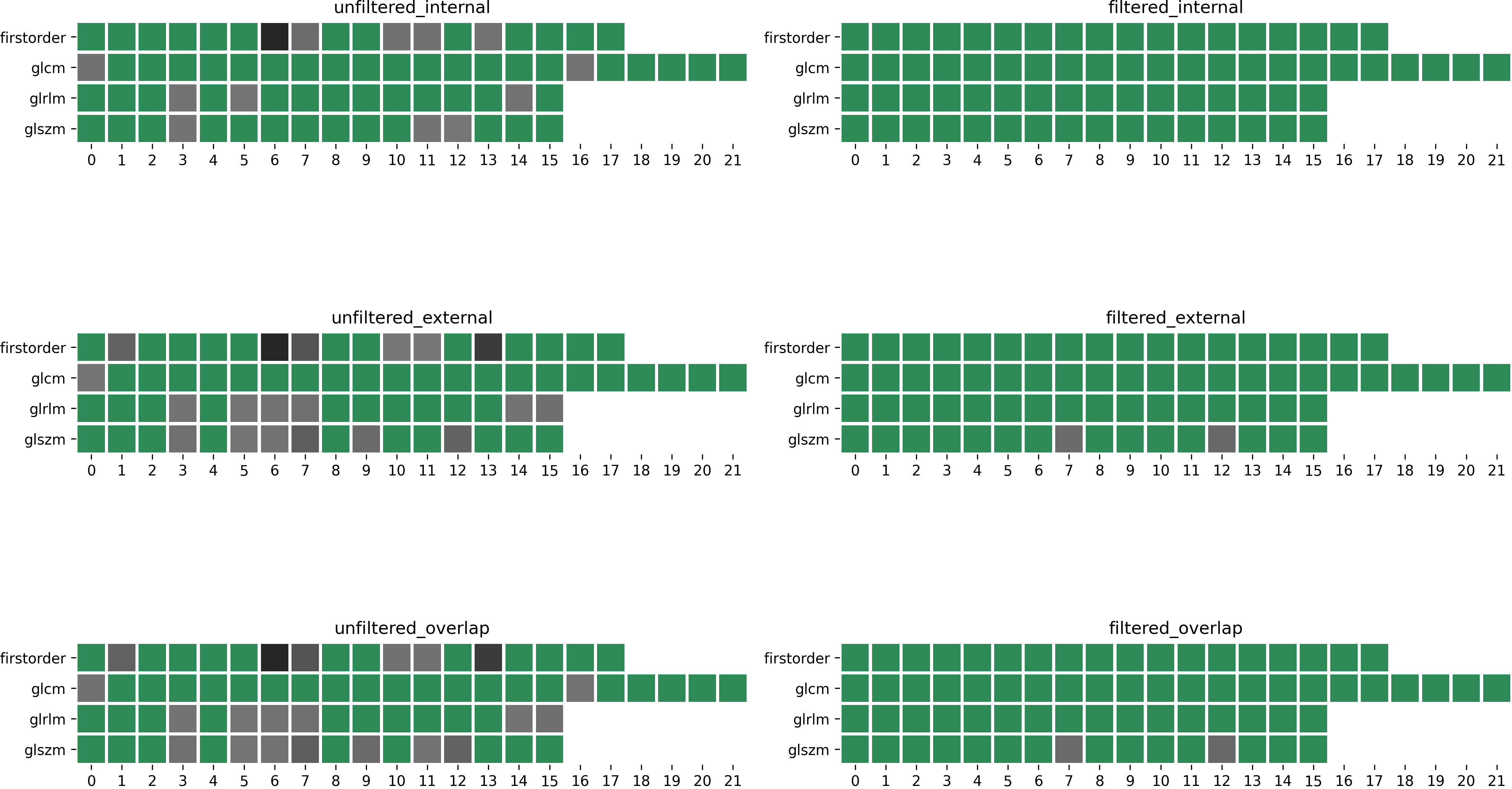

Supplement: Supplementary file 1 [file jpm-13-01172-s001.zip › heatmaps/sub_win/in_plane_random.png]

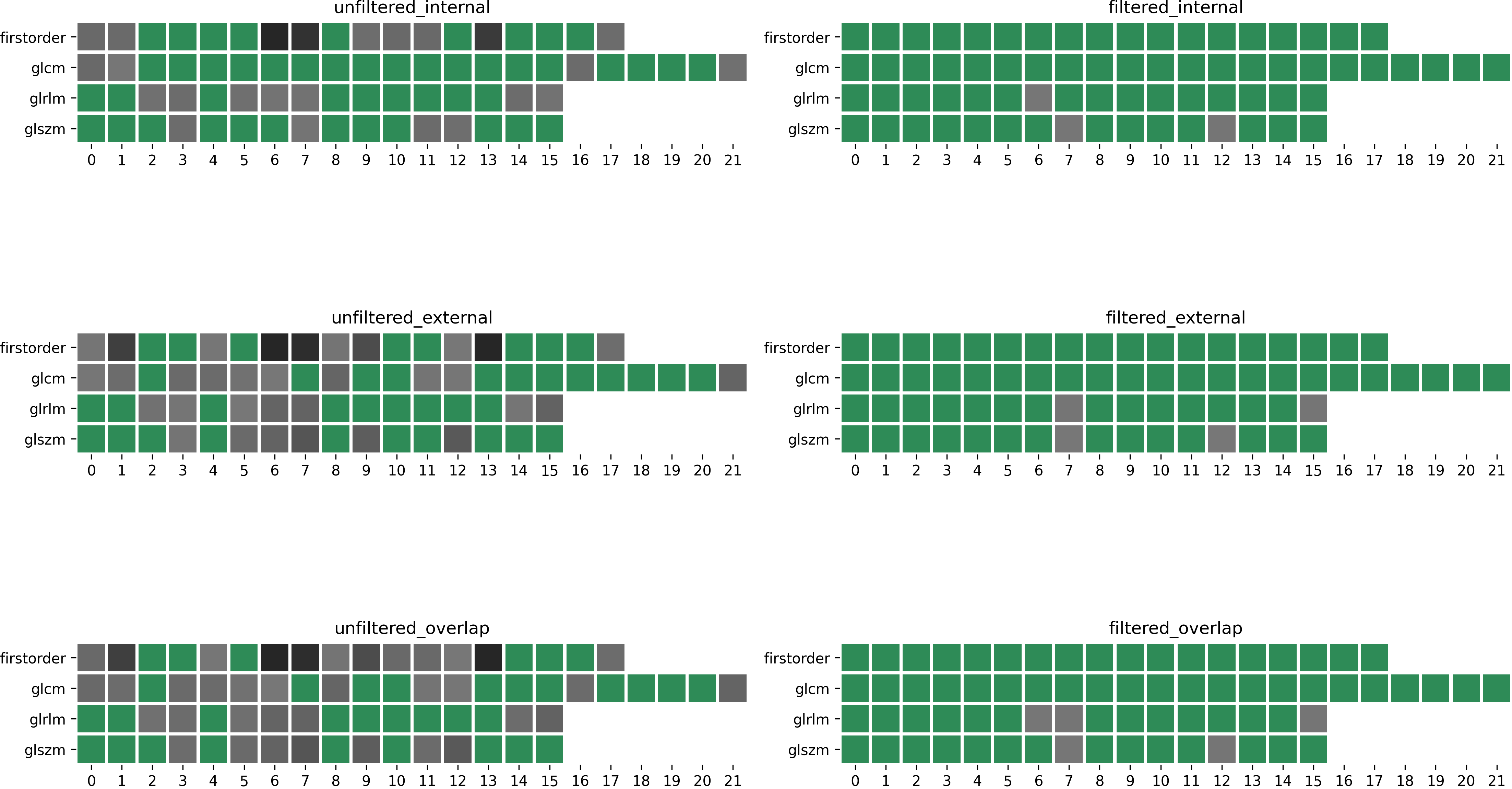

Supplement: Supplementary file 1 [file jpm-13-01172-s001.zip › heatmaps/sub_win/in_plane_systematic.png]

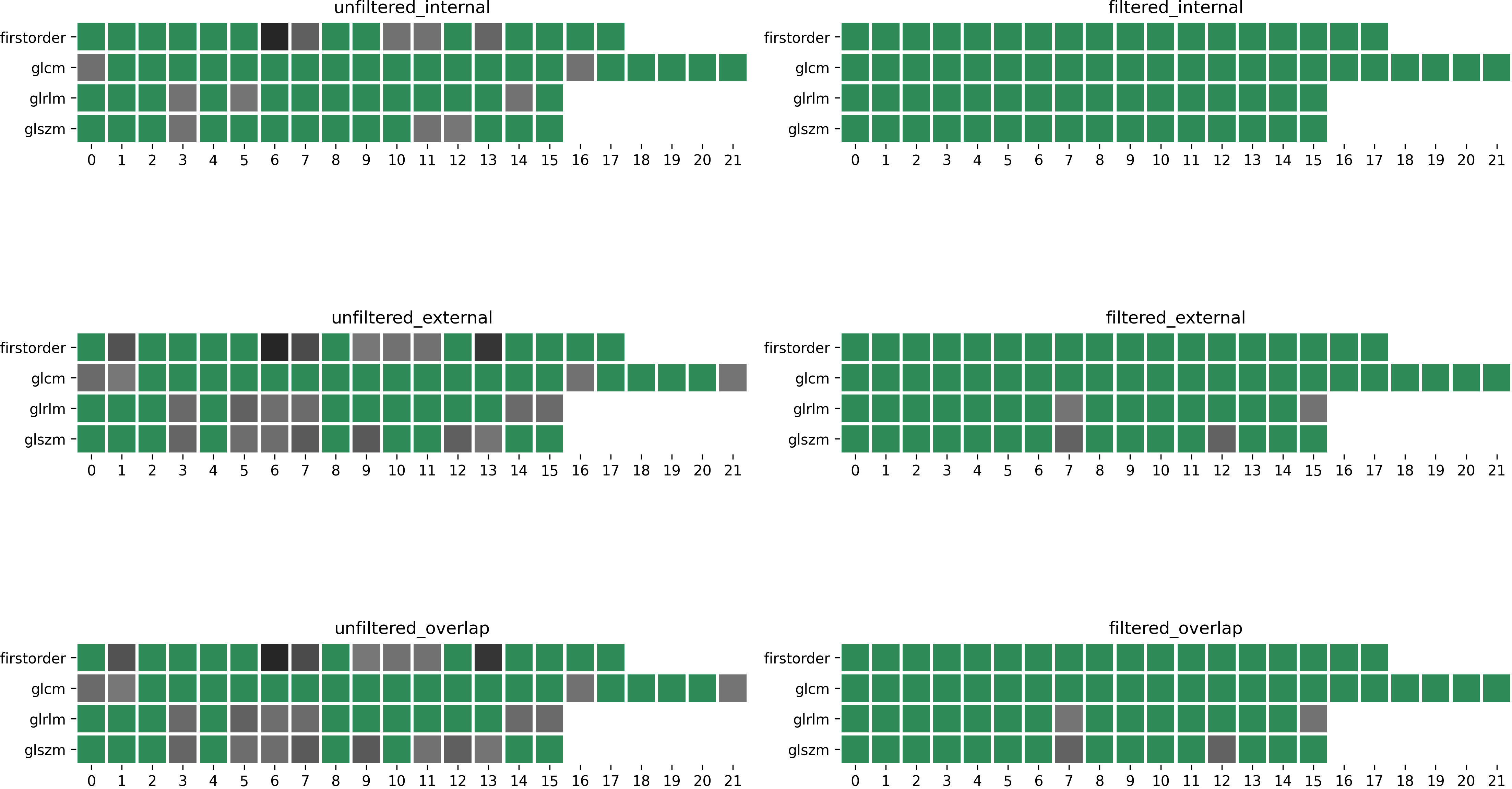

Supplement: Supplementary file 1 [file jpm-13-01172-s001.zip › heatmaps/sub_win/inout_plane_random.png]

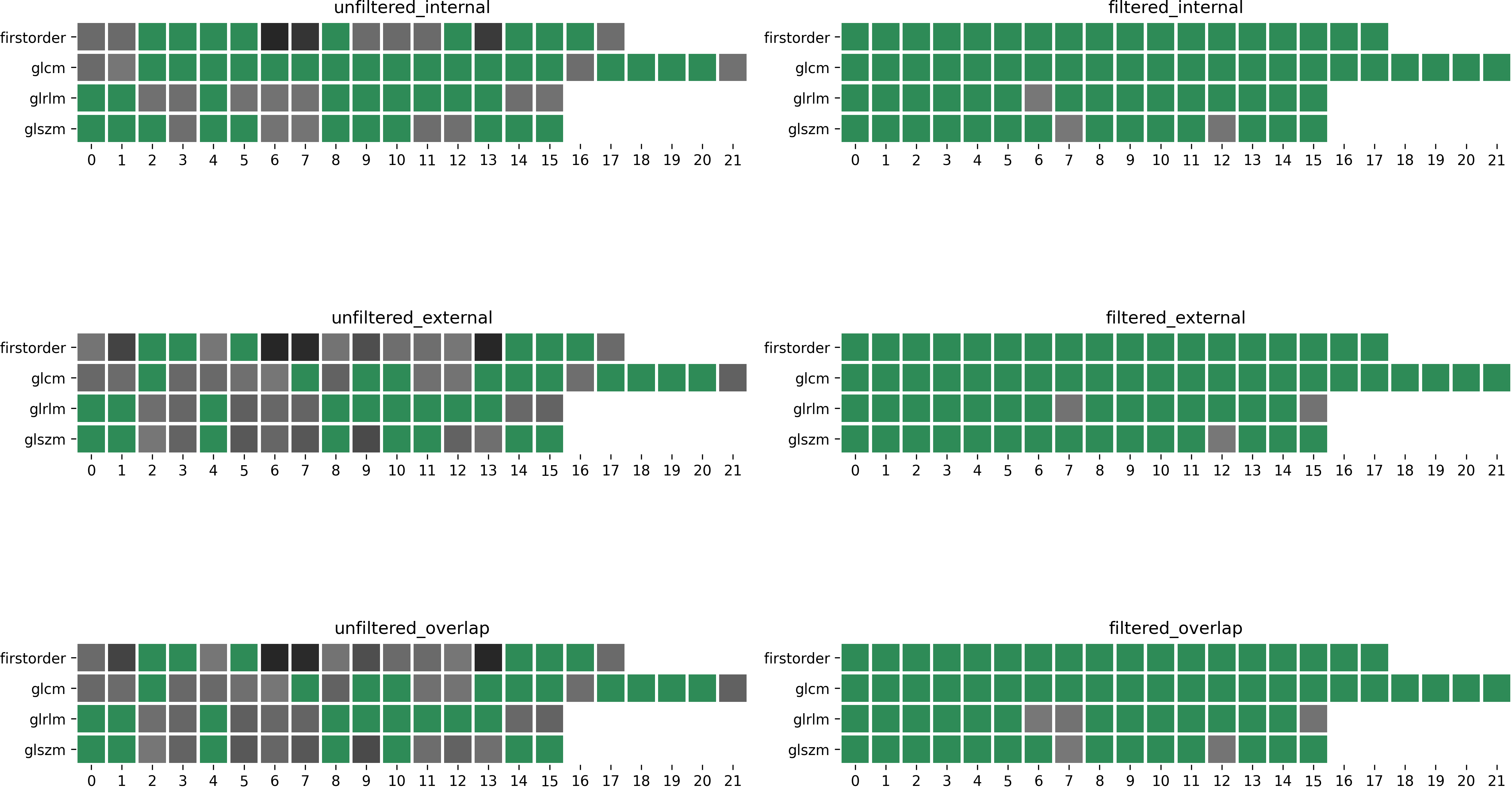

Supplement: Supplementary file 1 [file jpm-13-01172-s001.zip › heatmaps/sub_win/inout_plane_systematic.png]

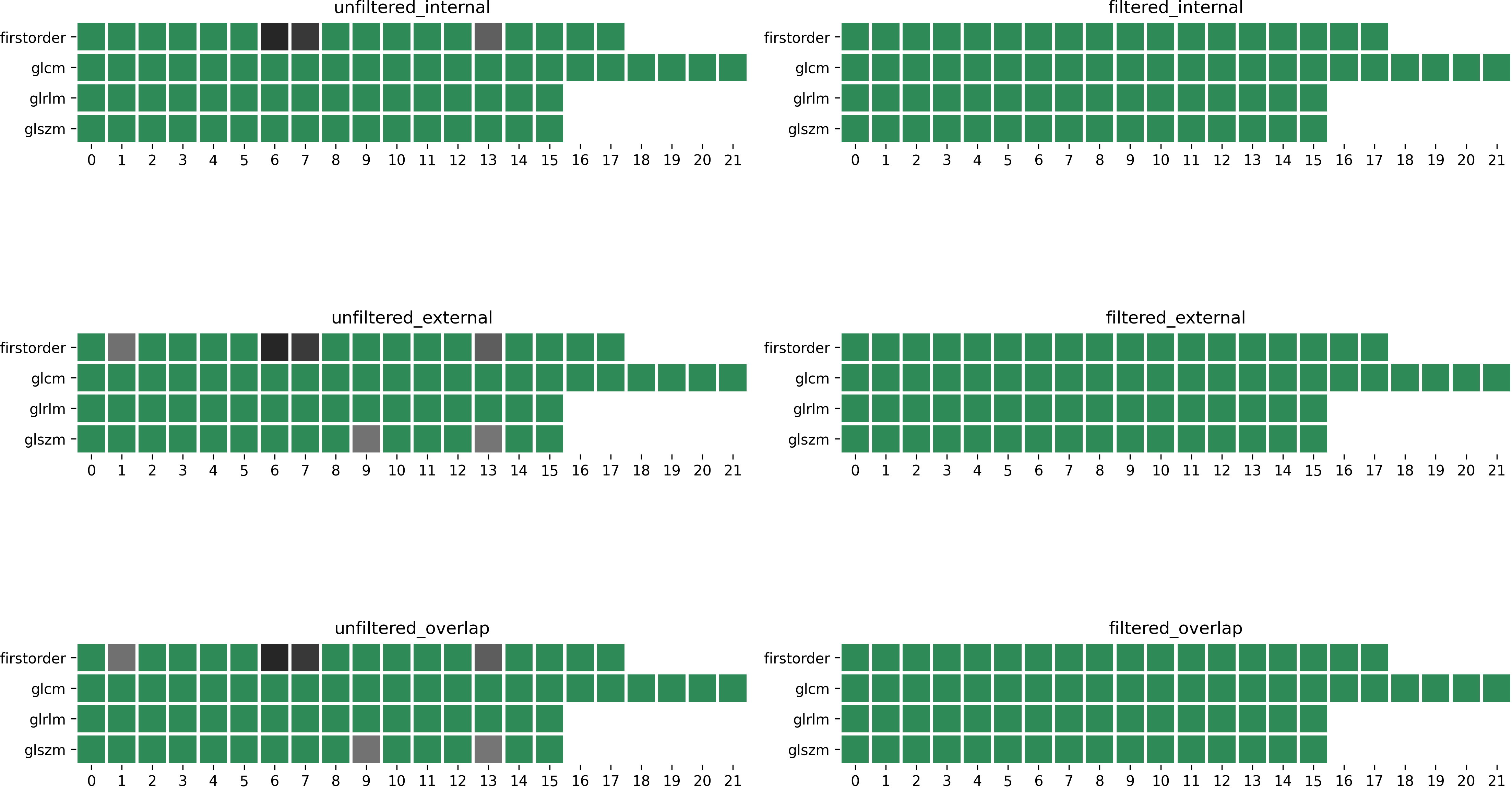

Supplement: Supplementary file 1 [file jpm-13-01172-s001.zip › heatmaps/sub_win/out_plane.png]

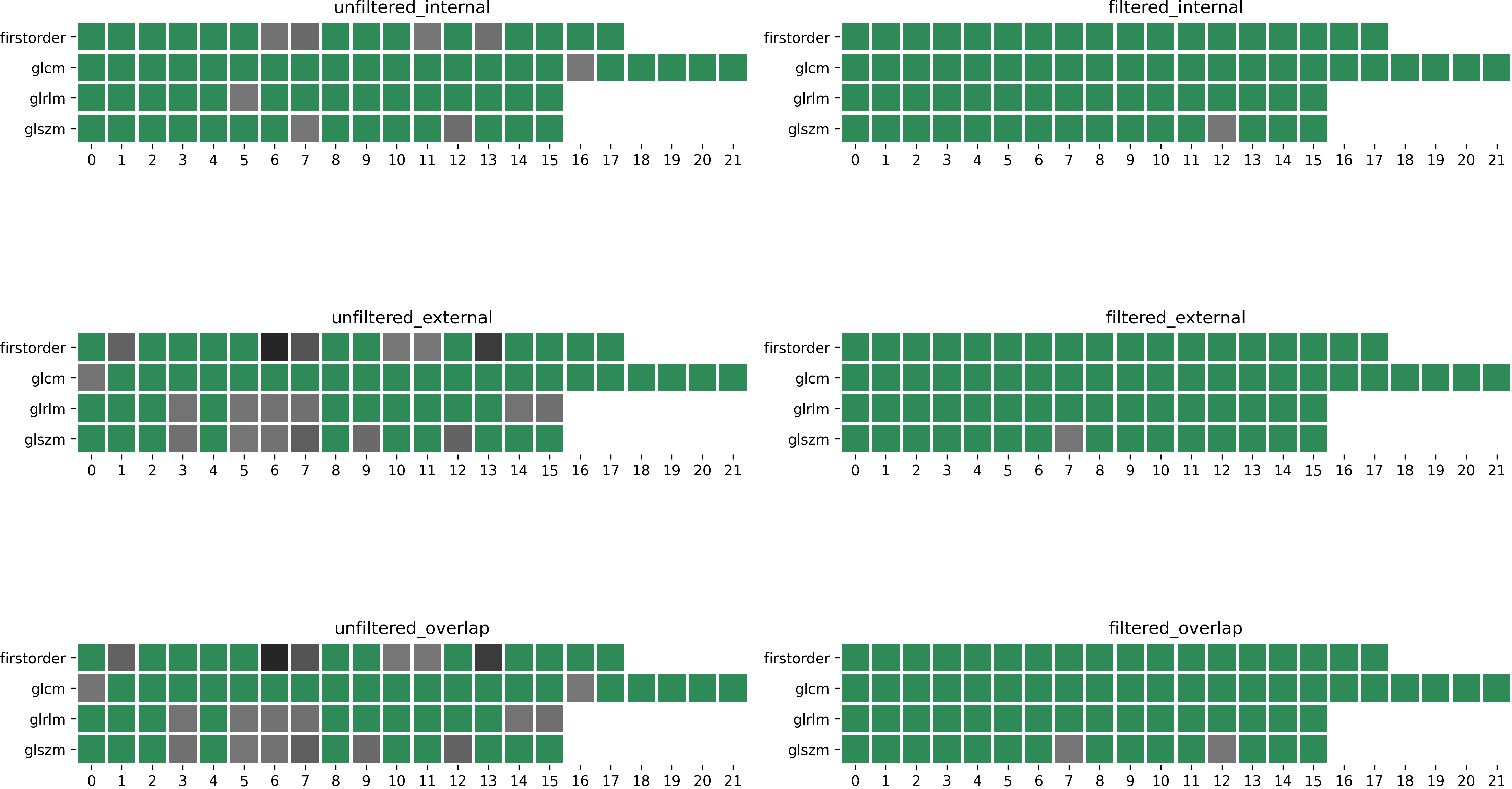

Supplement: Supplementary file 1 [file jpm-13-01172-s001.zip › heatmaps/sub_wout/in_plane_random.png]

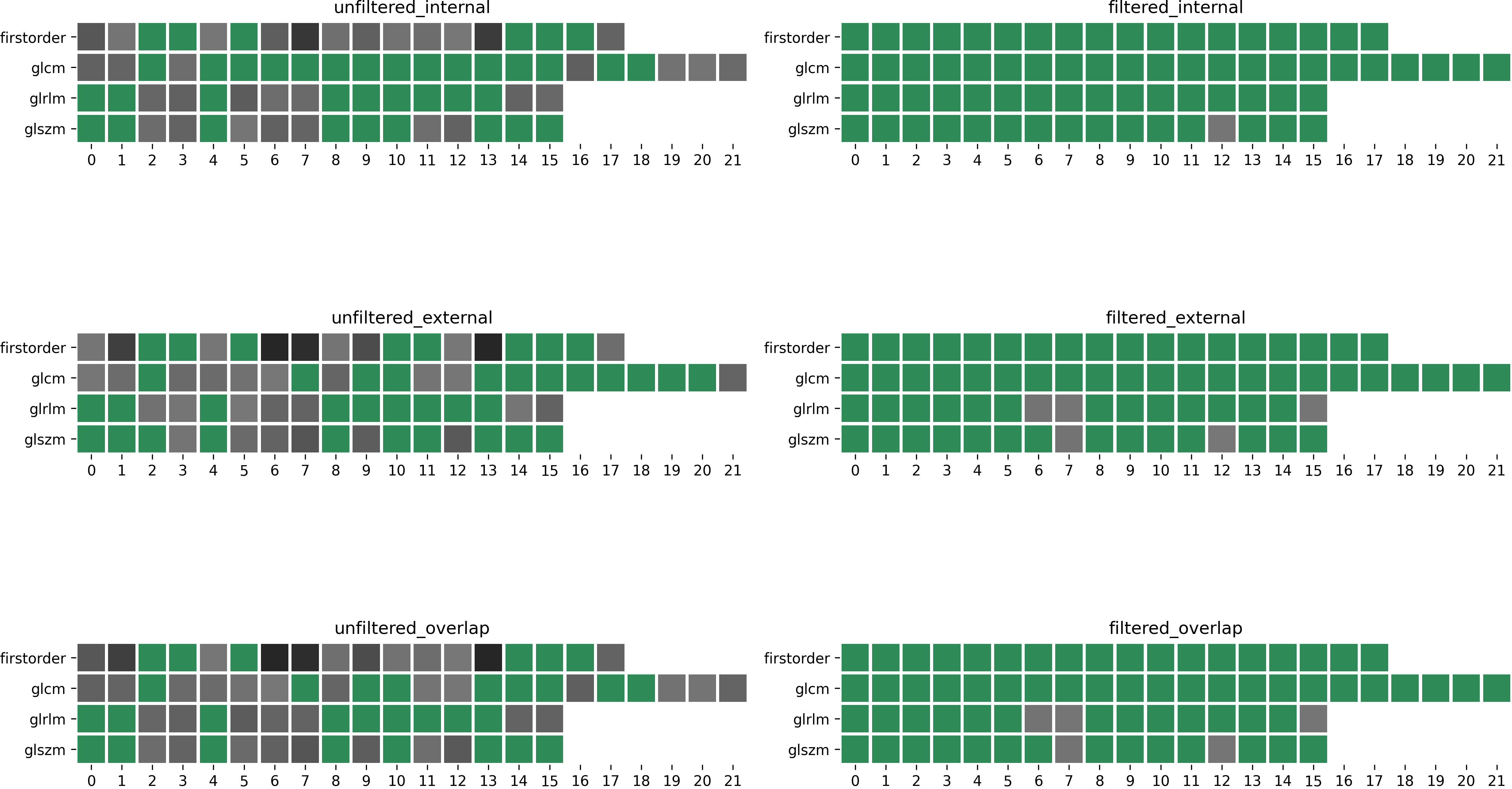

Supplement: Supplementary file 1 [file jpm-13-01172-s001.zip › heatmaps/sub_wout/in_plane_systematic.png]

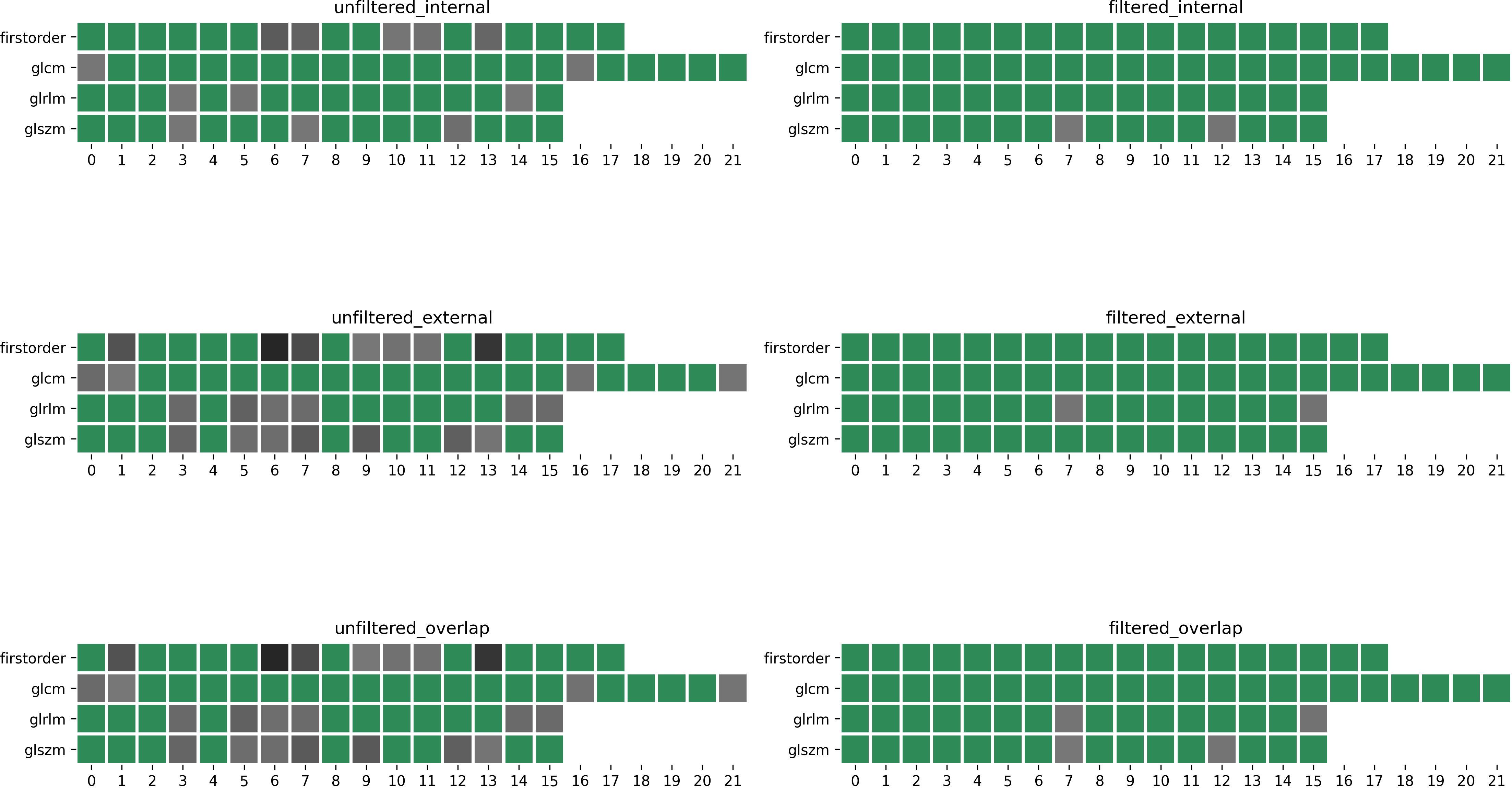

Supplement: Supplementary file 1 [file jpm-13-01172-s001.zip › heatmaps/sub_wout/inout_plane_random.png]

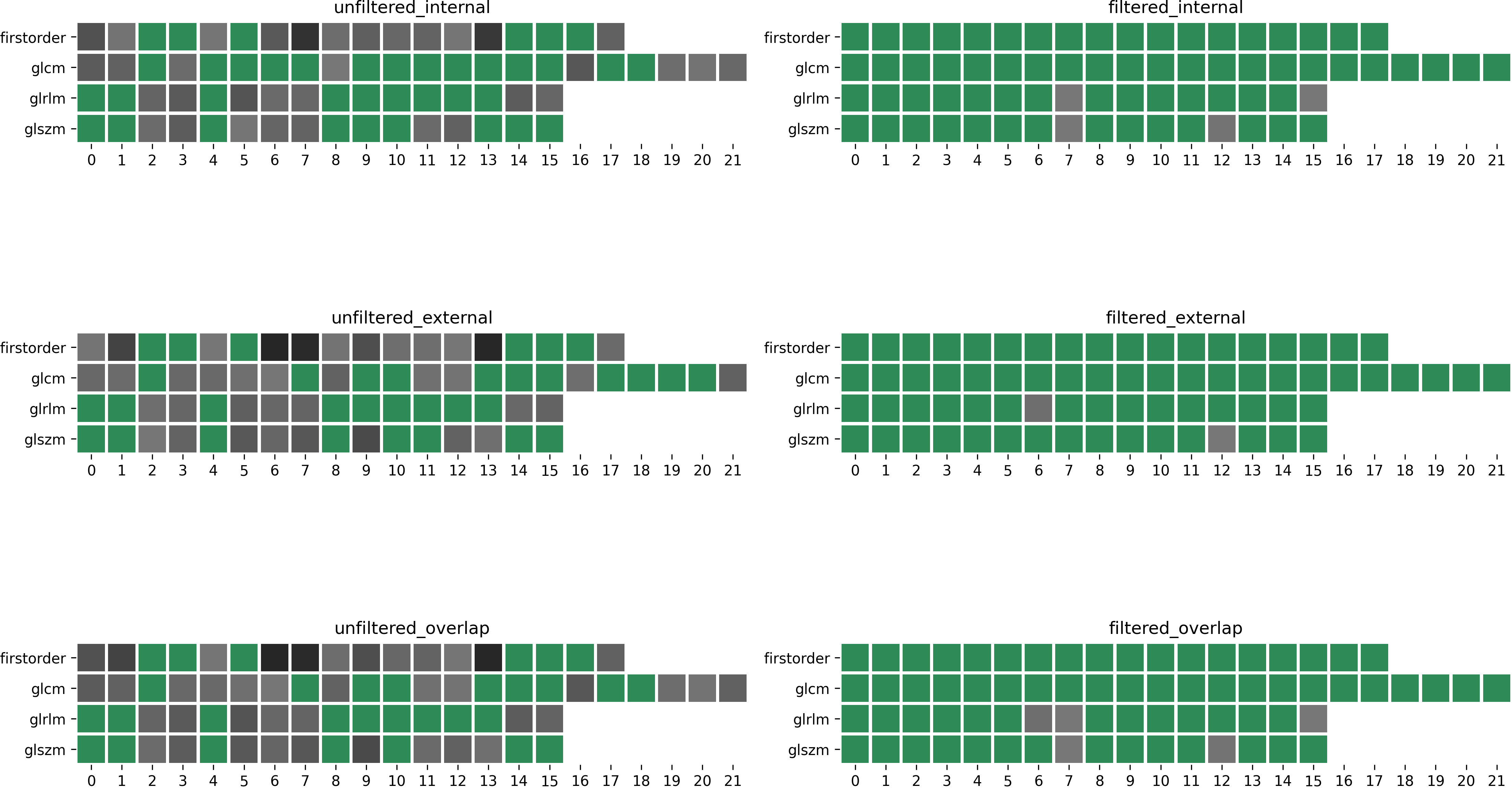

Supplement: Supplementary file 1 [file jpm-13-01172-s001.zip › heatmaps/sub_wout/inout_plane_systematic.png]

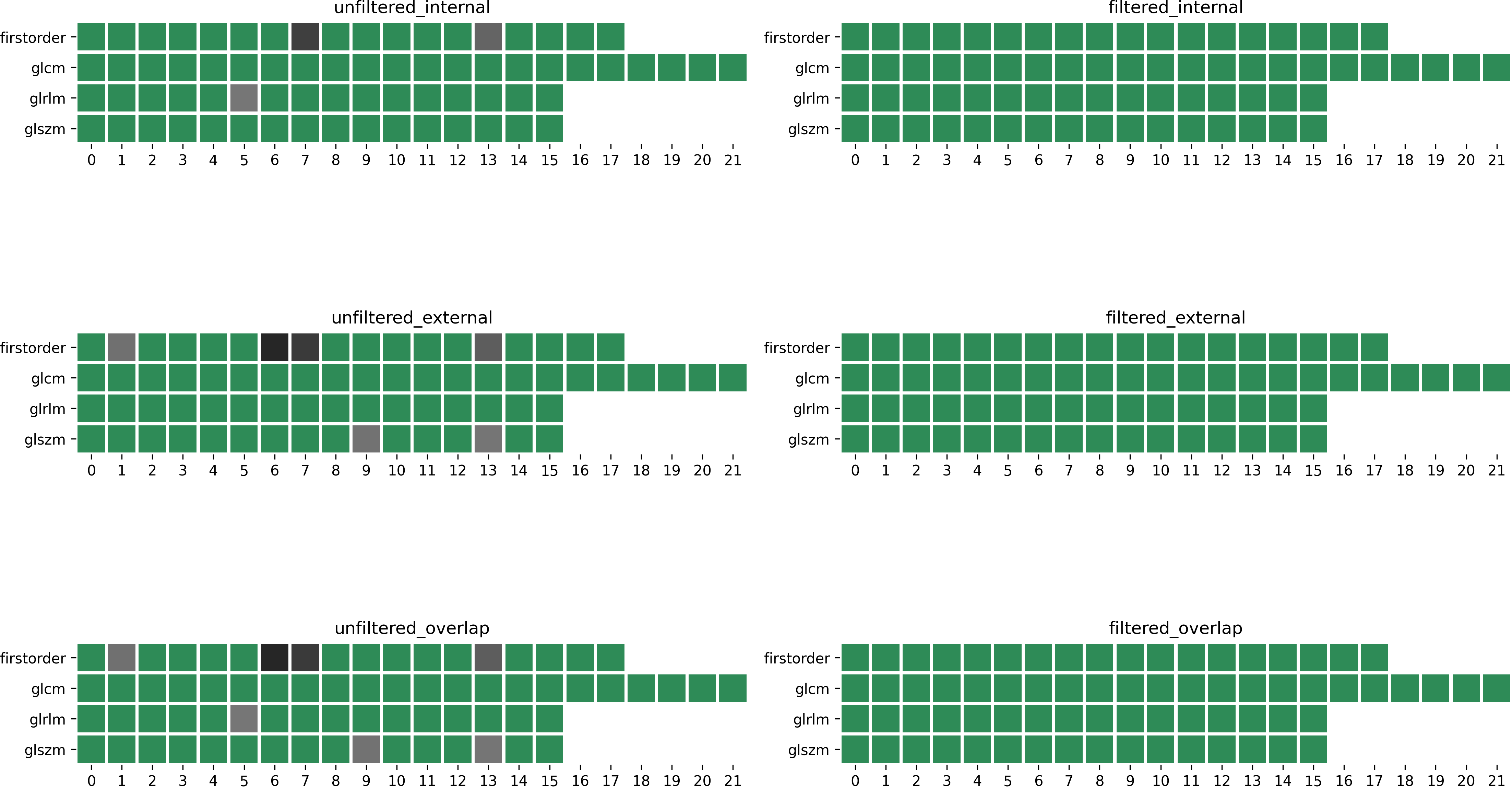

Supplement: Supplementary file 1 [file jpm-13-01172-s001.zip › heatmaps/sub_wout/out_plane.png]

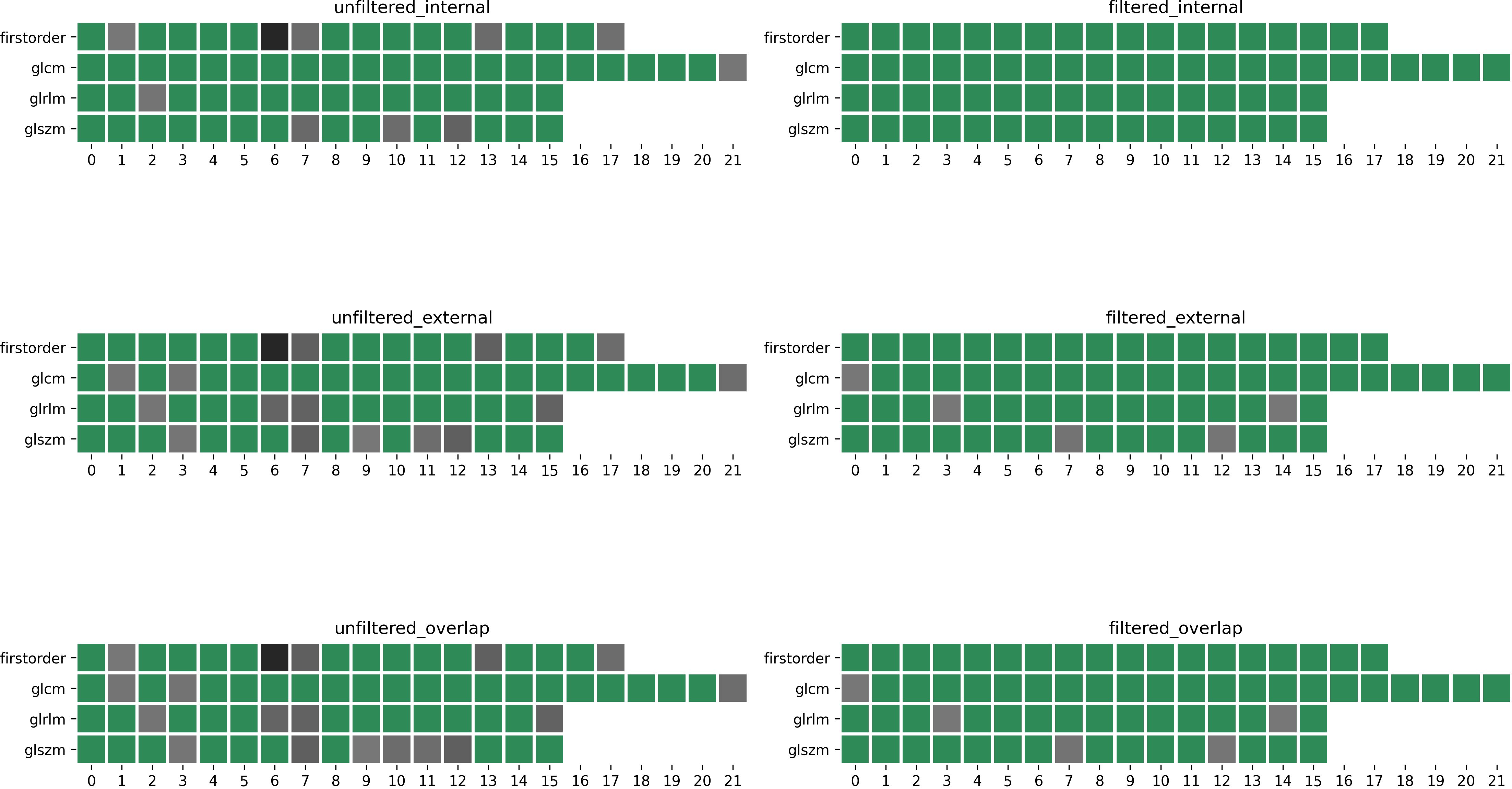

Supplement: Supplementary file 1 [file jpm-13-01172-s001.zip › heatmaps/t2w/in_plane_random.png]

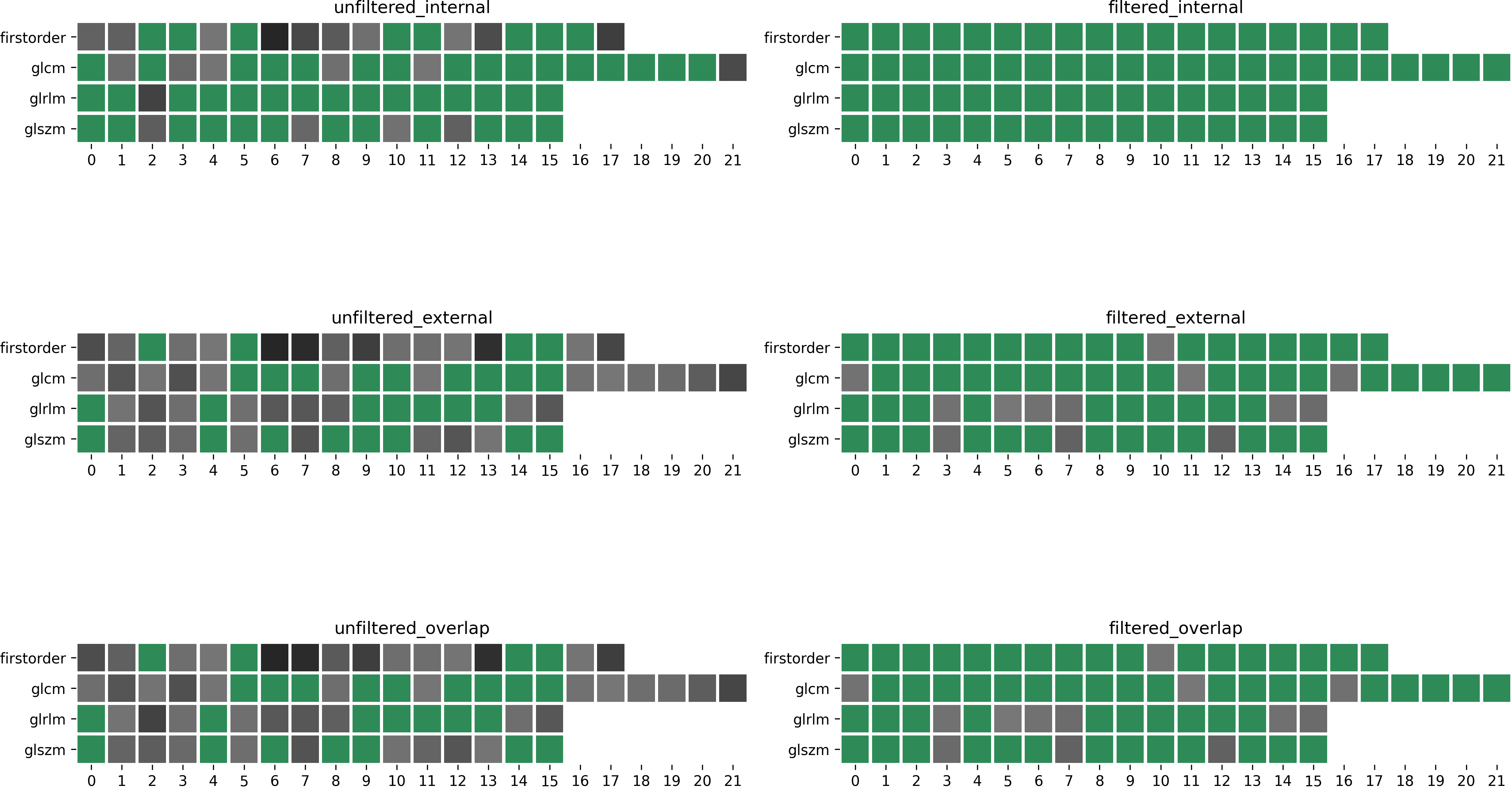

Supplement: Supplementary file 1 [file jpm-13-01172-s001.zip › heatmaps/t2w/in_plane_systematic.png]

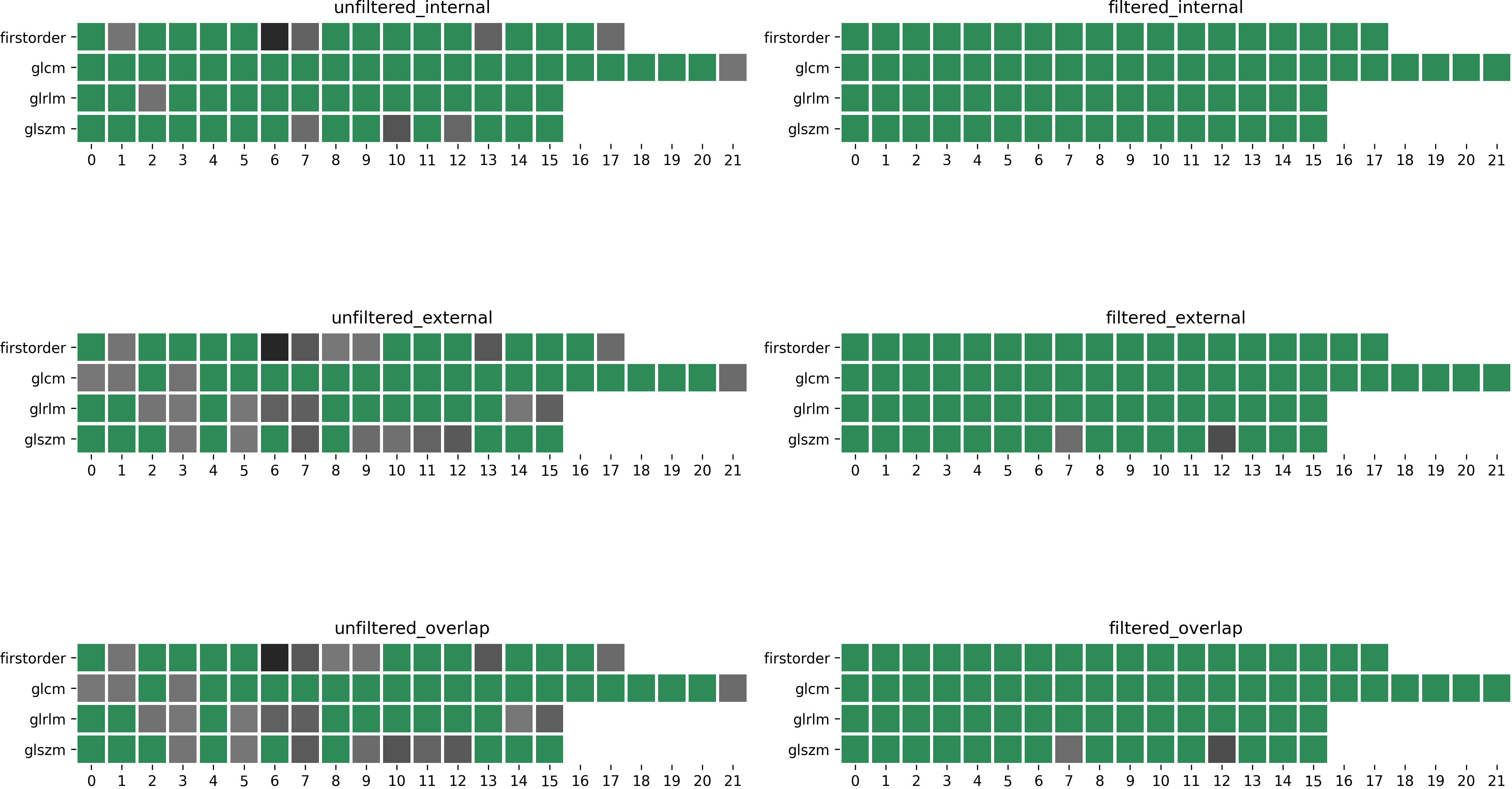

Supplement: Supplementary file 1 [file jpm-13-01172-s001.zip › heatmaps/t2w/inout_plane_random.png]

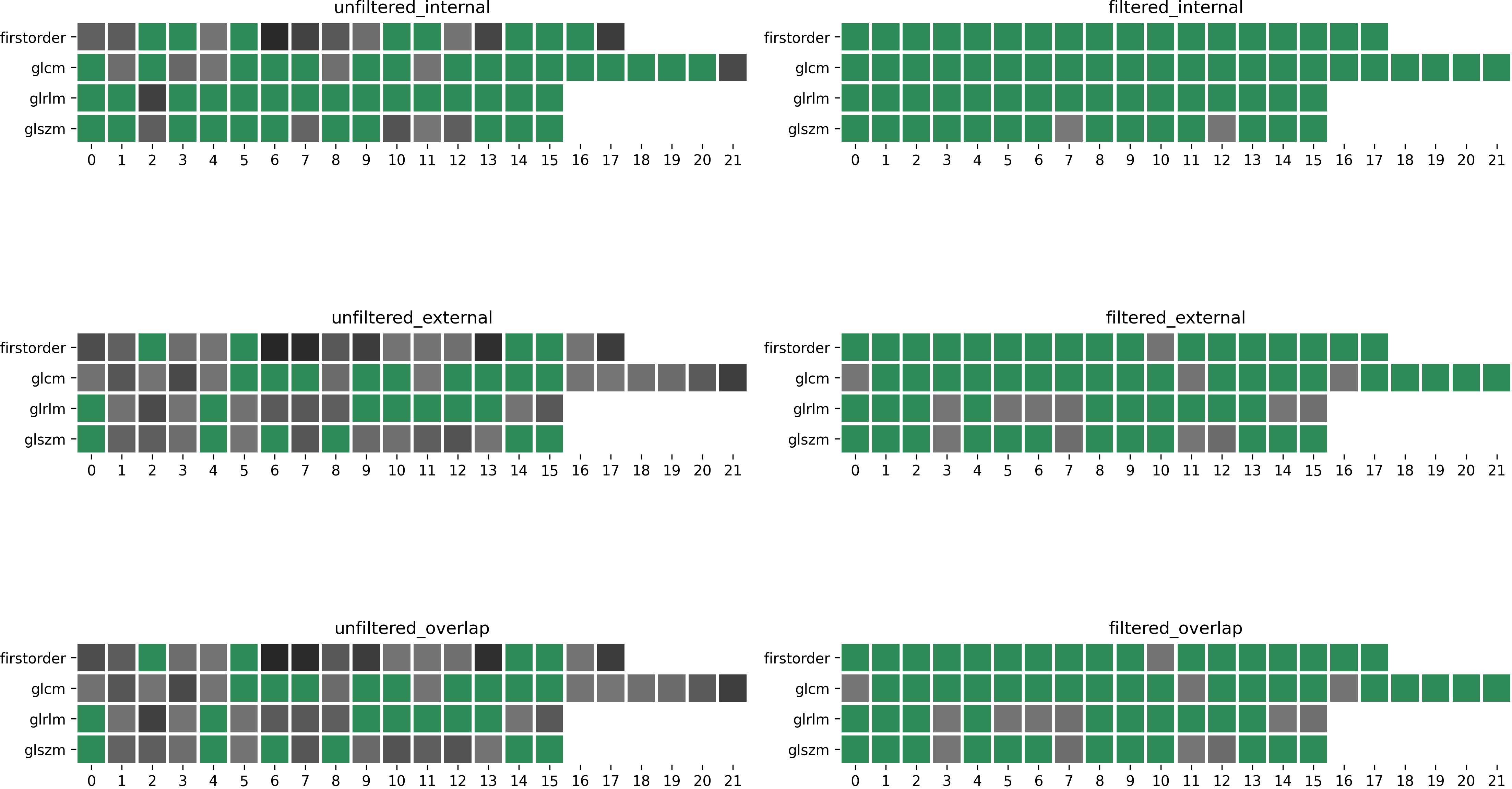

Supplement: Supplementary file 1 [file jpm-13-01172-s001.zip › heatmaps/t2w/inout_plane_systematic.png]

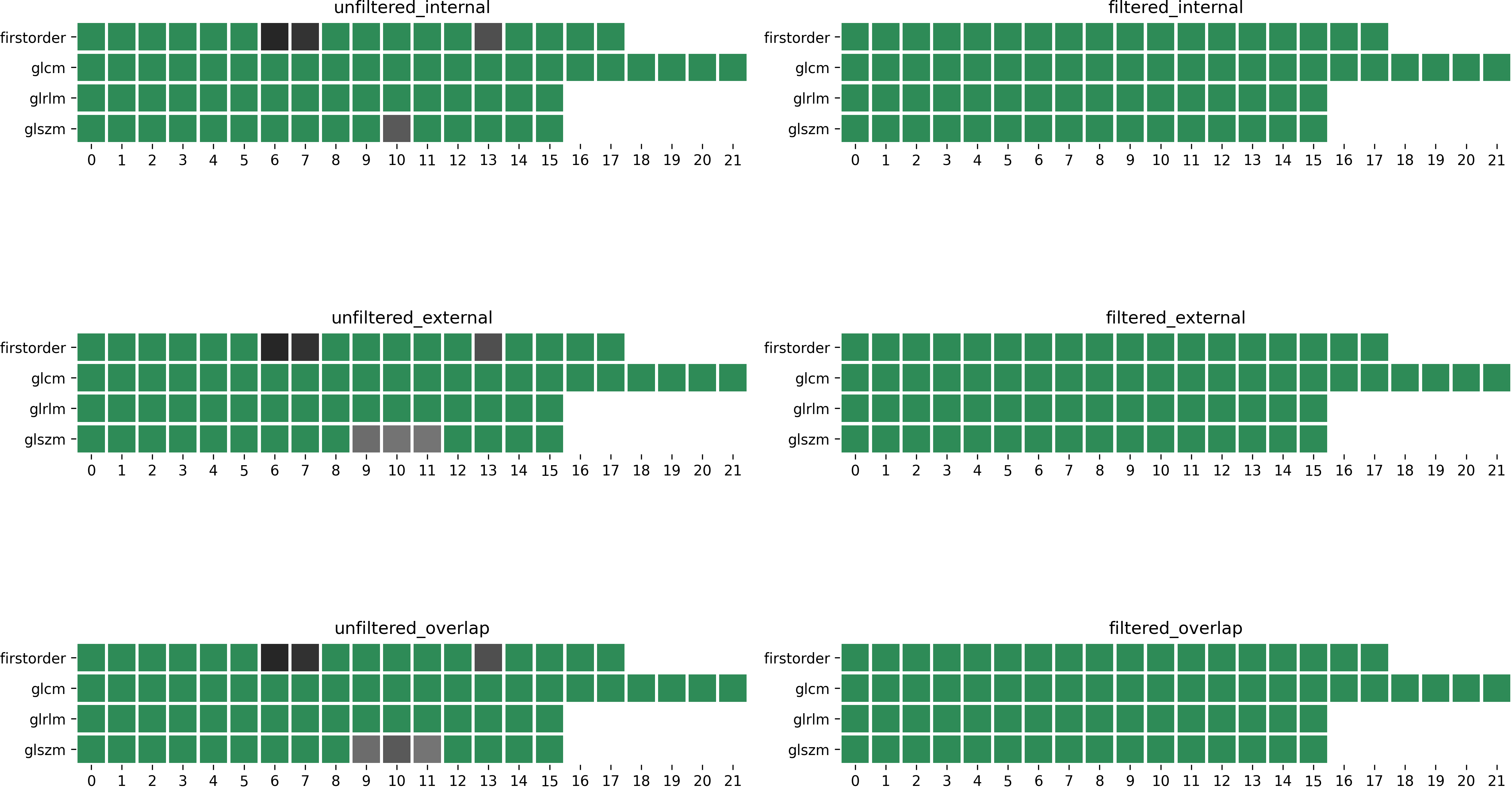

Supplement: Supplementary file 1 [file jpm-13-01172-s001.zip › heatmaps/t2w/out_plane.png]

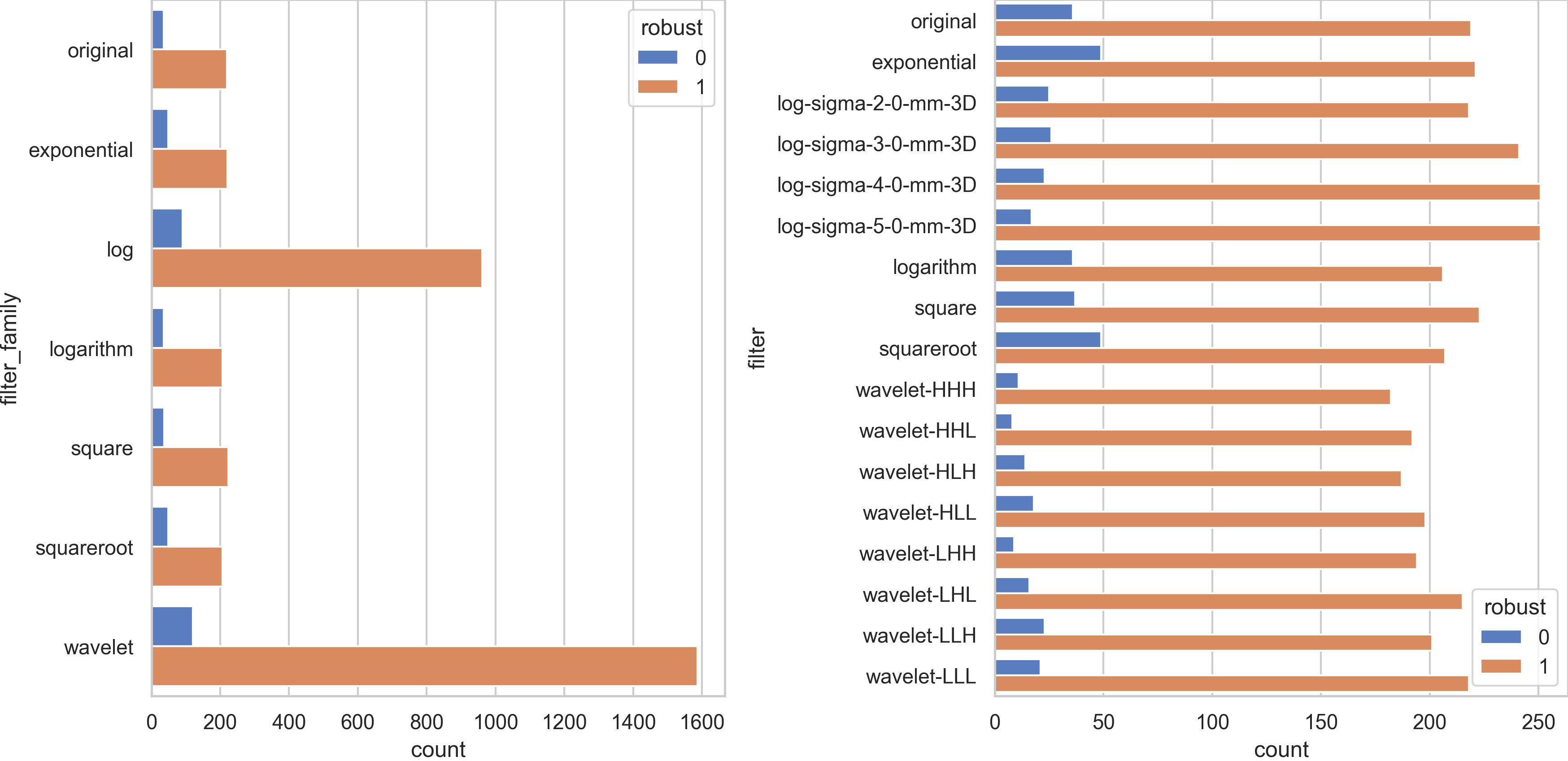

Supplement: Supplementary file 1 [file jpm-13-01172-s001.zip › histplots/in_plane_random.png]

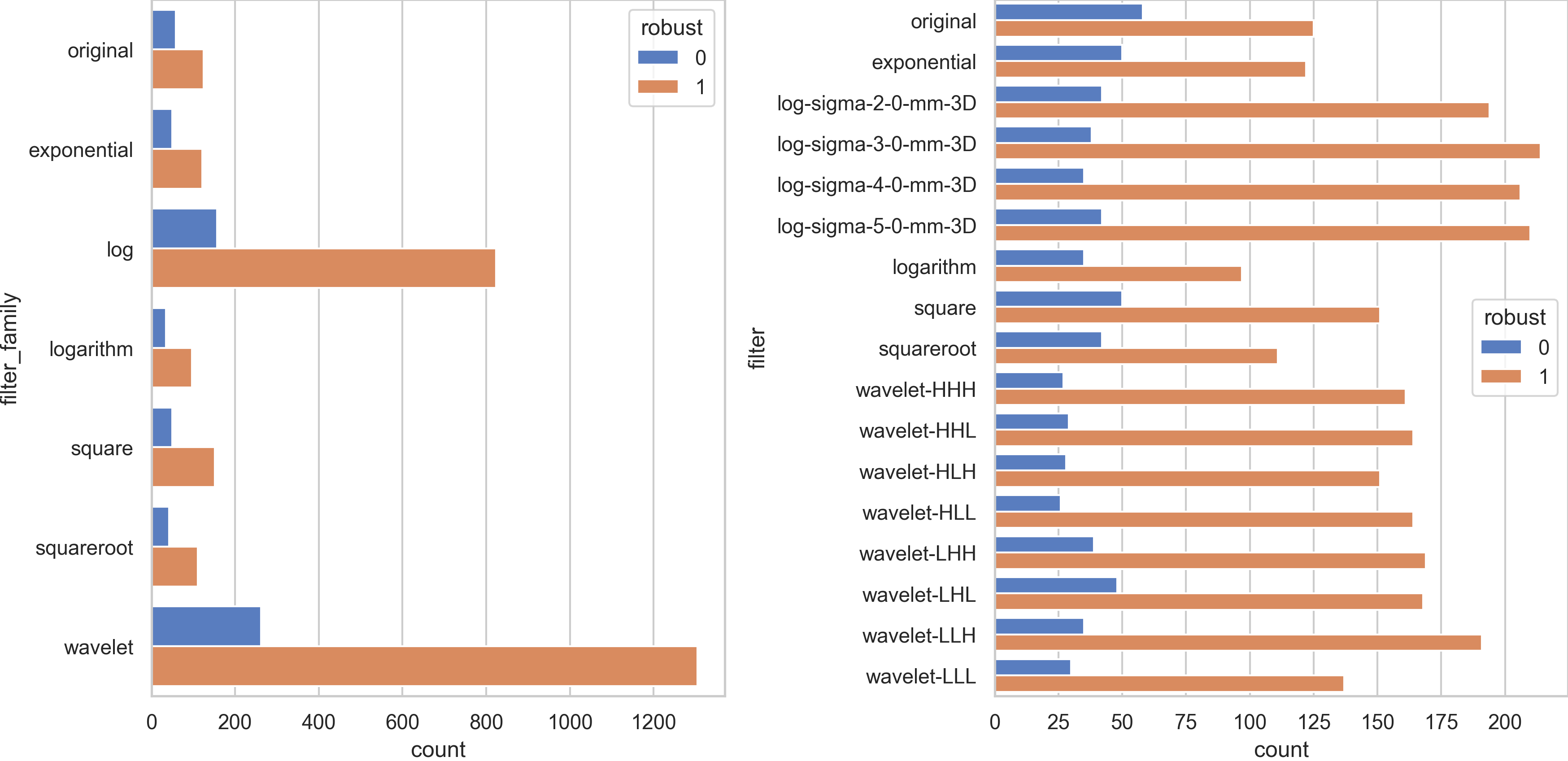

Supplement: Supplementary file 1 [file jpm-13-01172-s001.zip › histplots/in_plane_systematic.png]

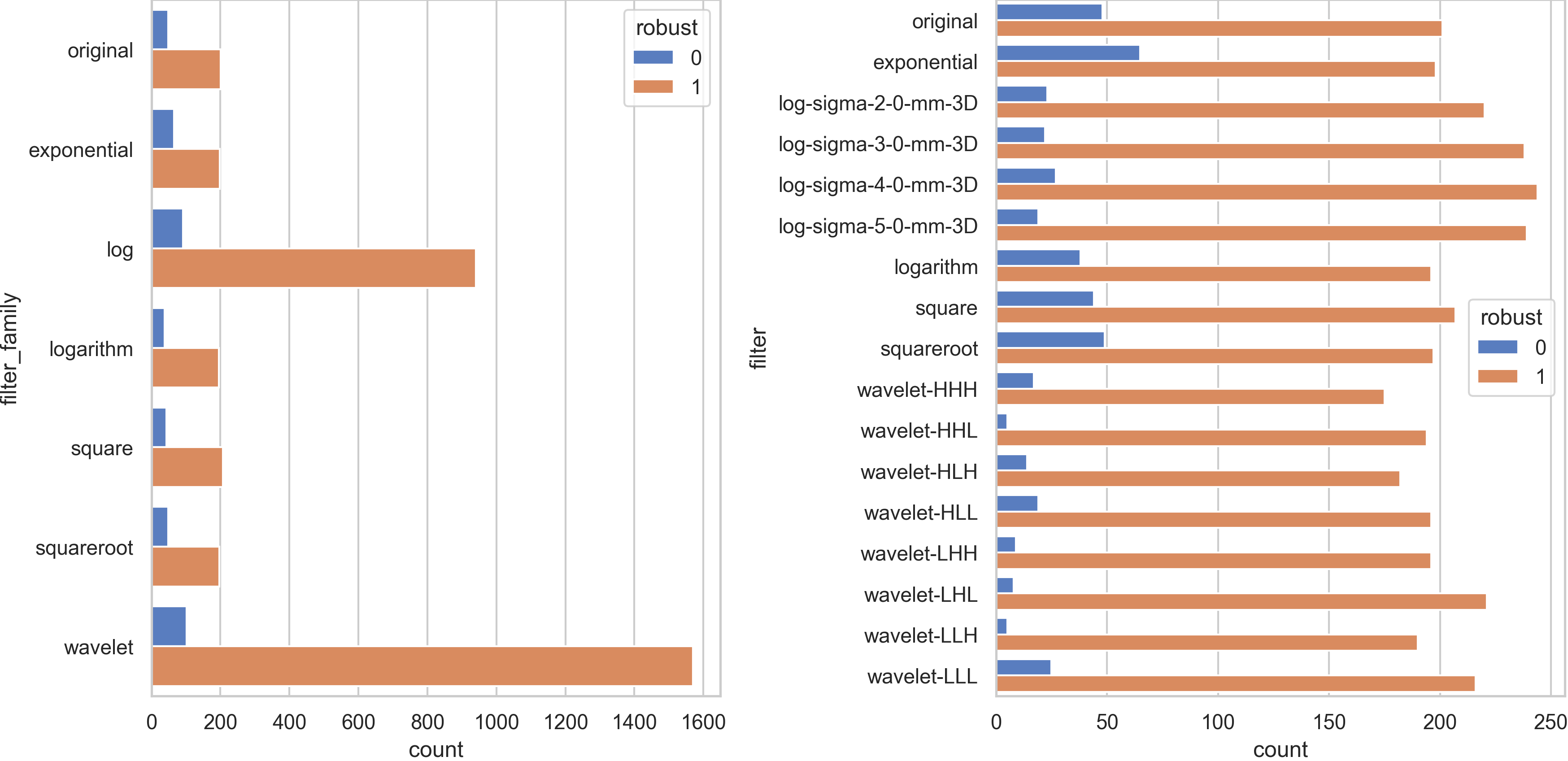

Supplement: Supplementary file 1 [file jpm-13-01172-s001.zip › histplots/inout_plane_random.png]

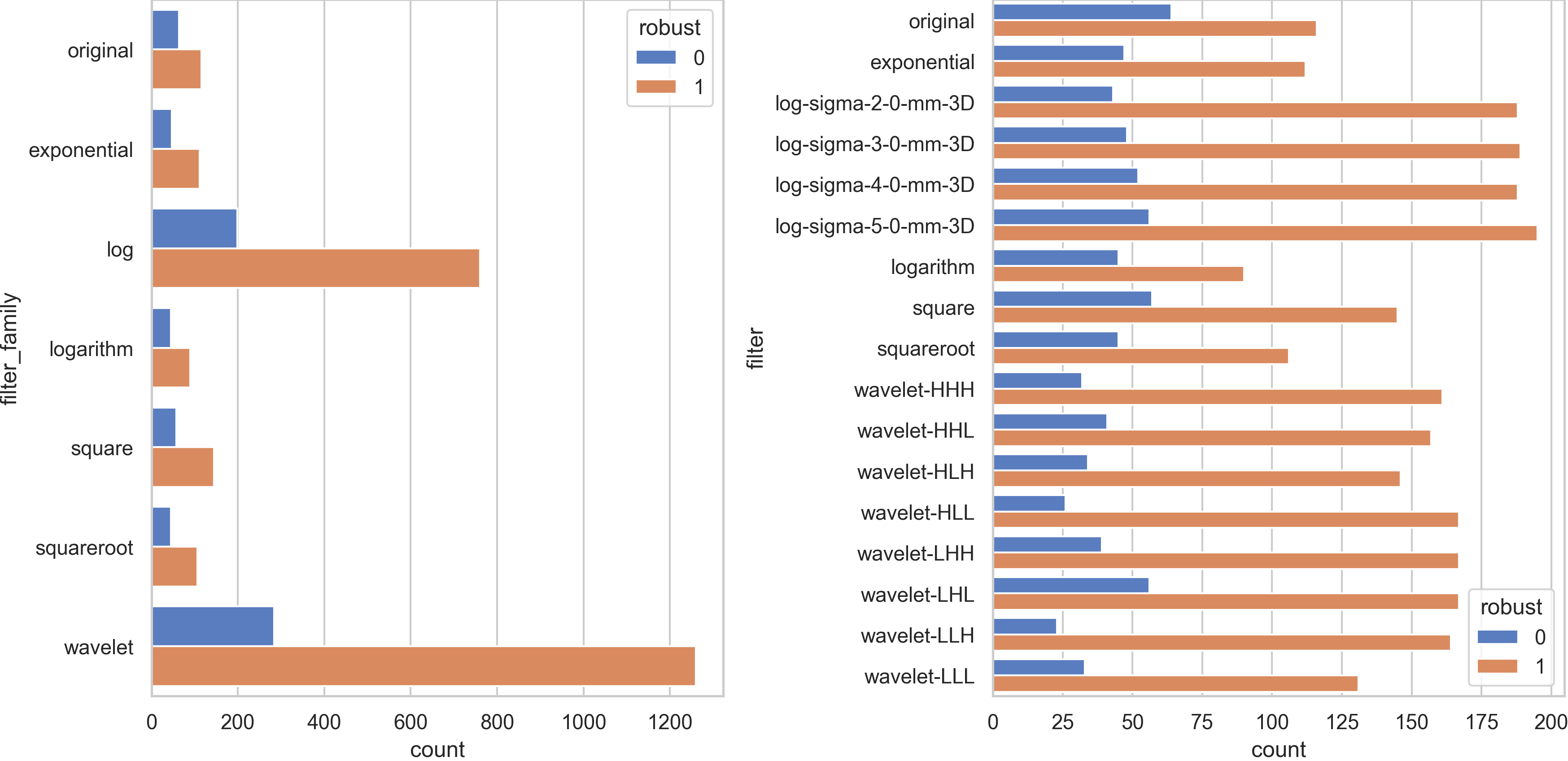

Supplement: Supplementary file 1 [file jpm-13-01172-s001.zip › histplots/inout_plane_systematic.png]

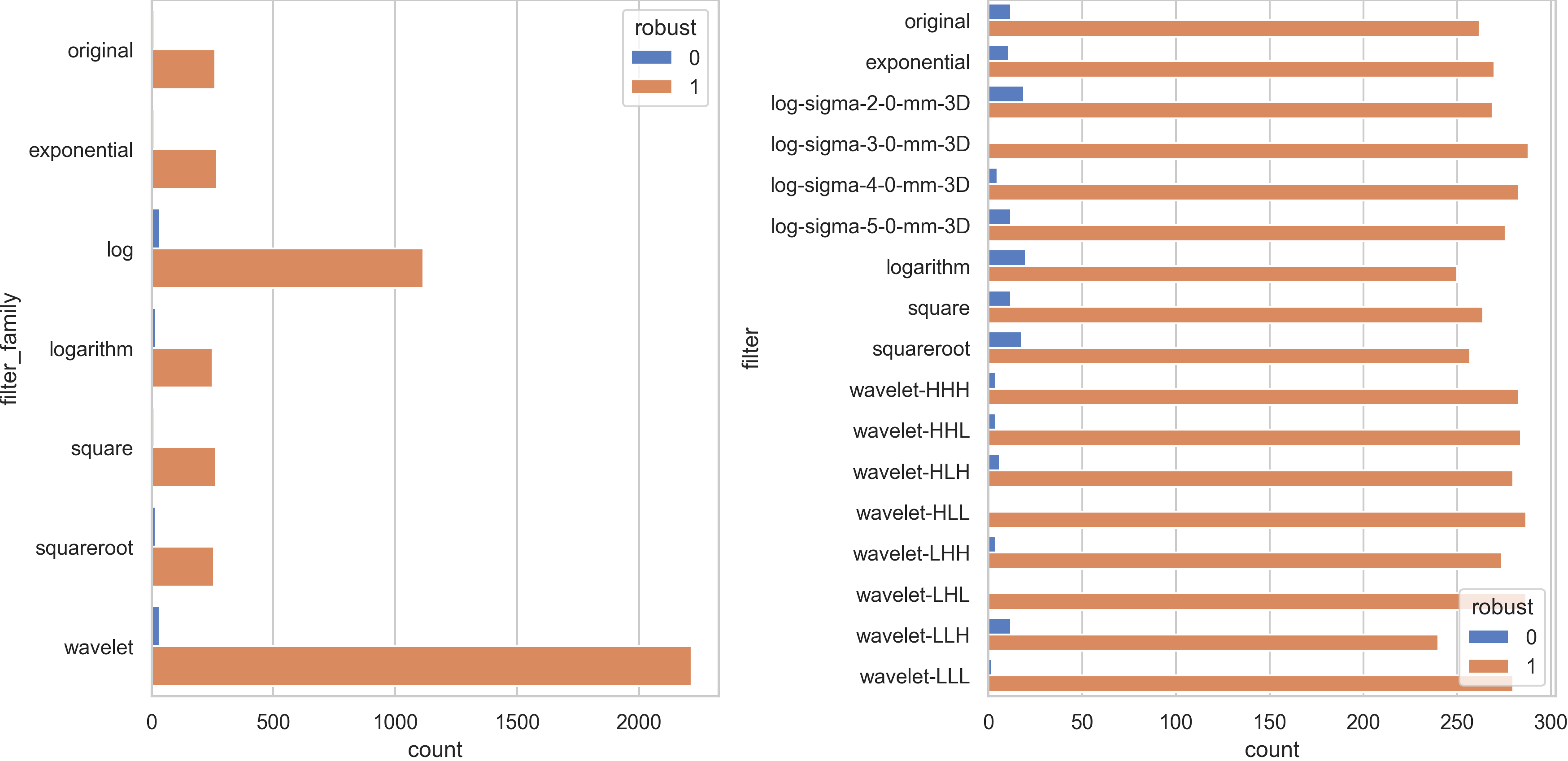

Supplement: Supplementary file 1 [file jpm-13-01172-s001.zip › histplots/out_plane.png]

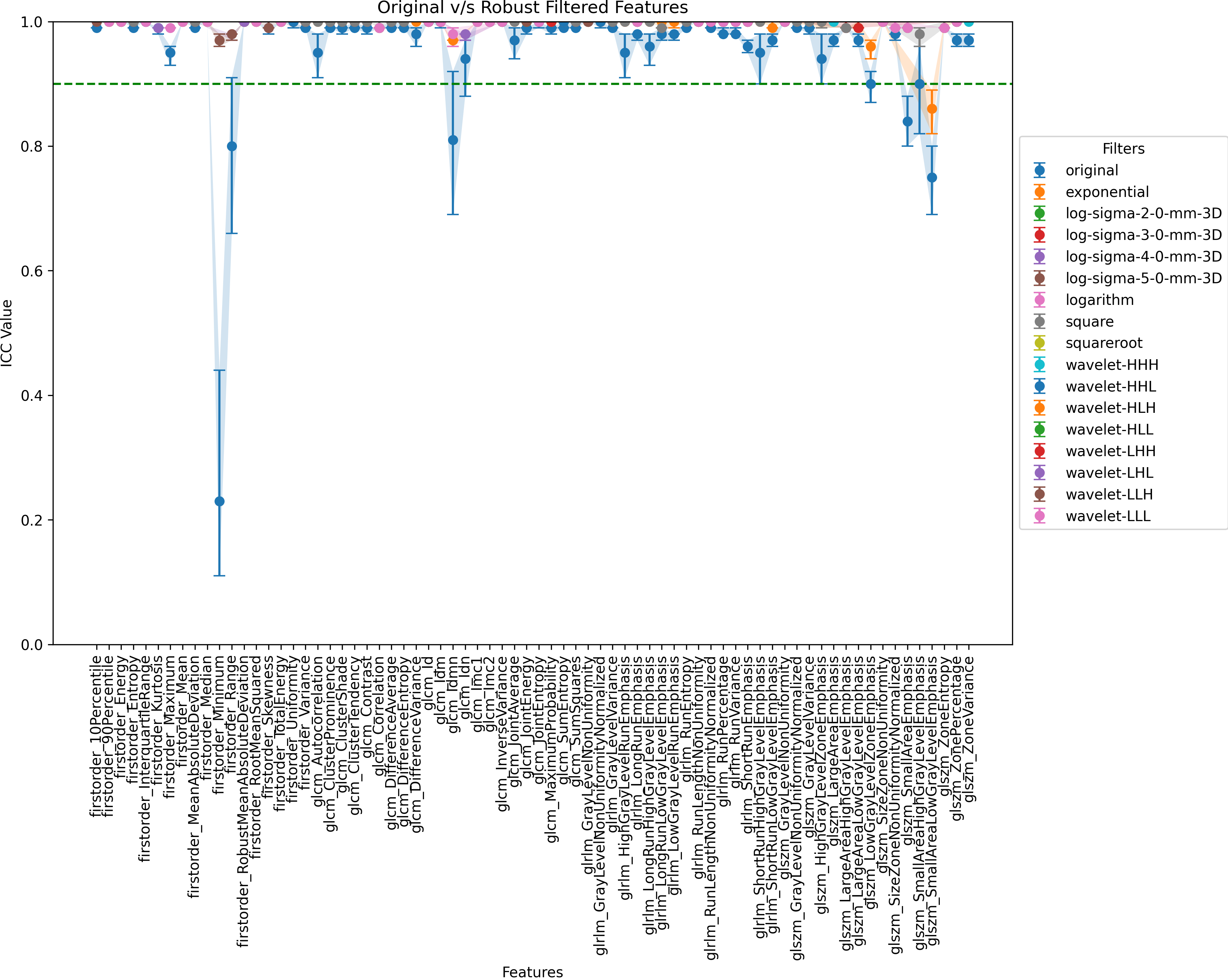

Supplement: Supplementary file 1 [file jpm-13-01172-s001.zip › overlap_plots/adc/in_plane_random.png]

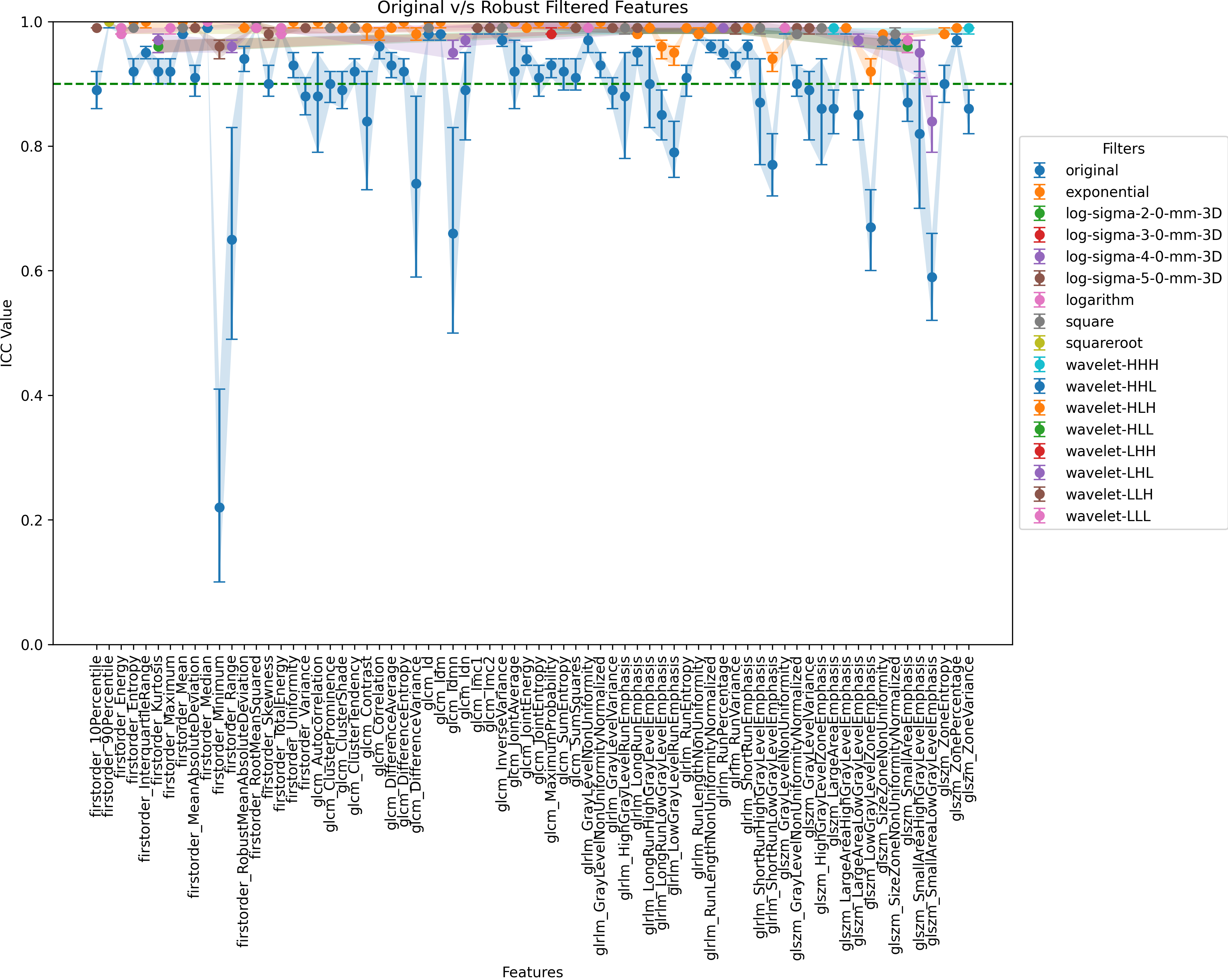

Supplement: Supplementary file 1 [file jpm-13-01172-s001.zip › overlap_plots/adc/in_plane_systematic.png]

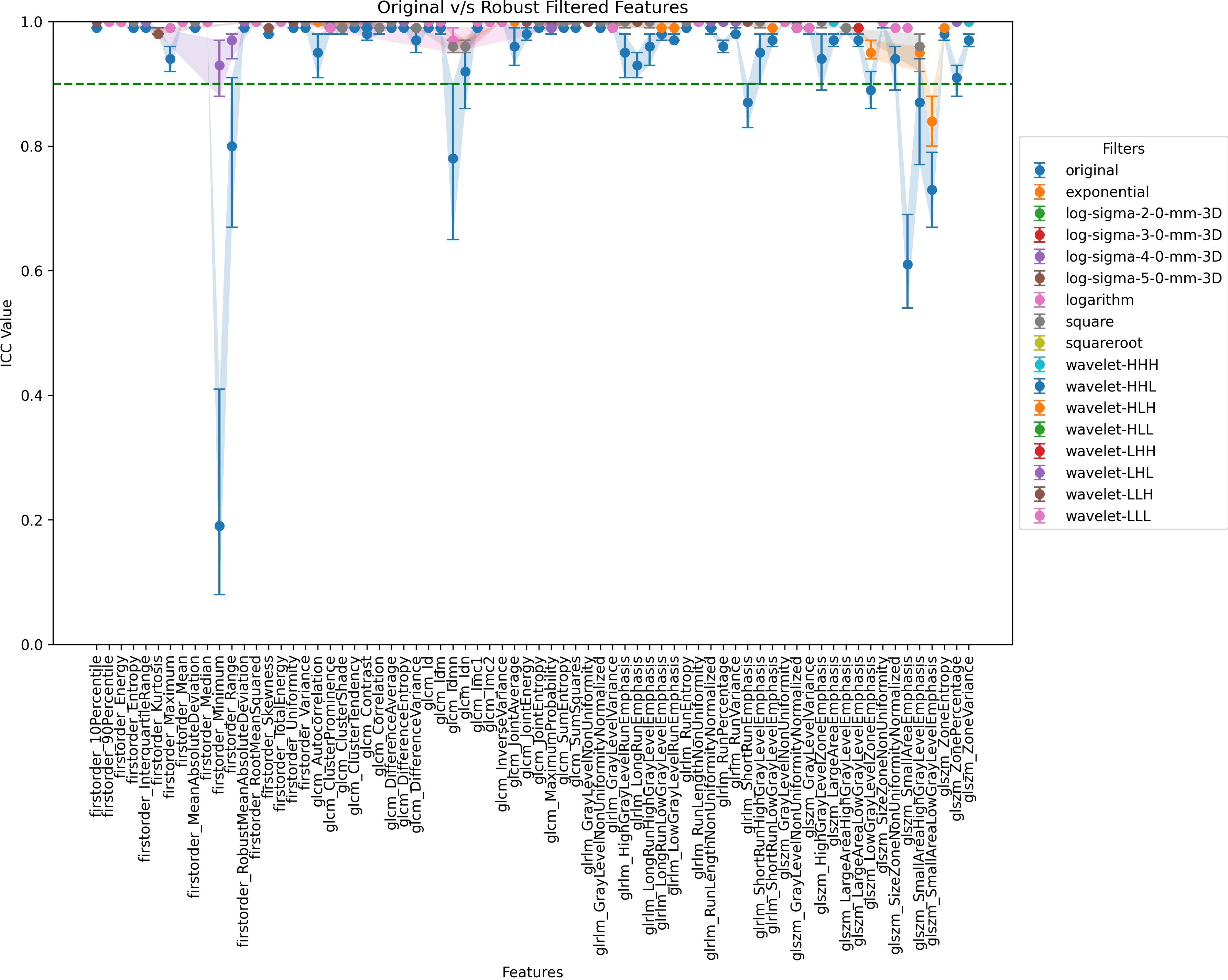

Supplement: Supplementary file 1 [file jpm-13-01172-s001.zip › overlap_plots/adc/inout_plane_random.png]

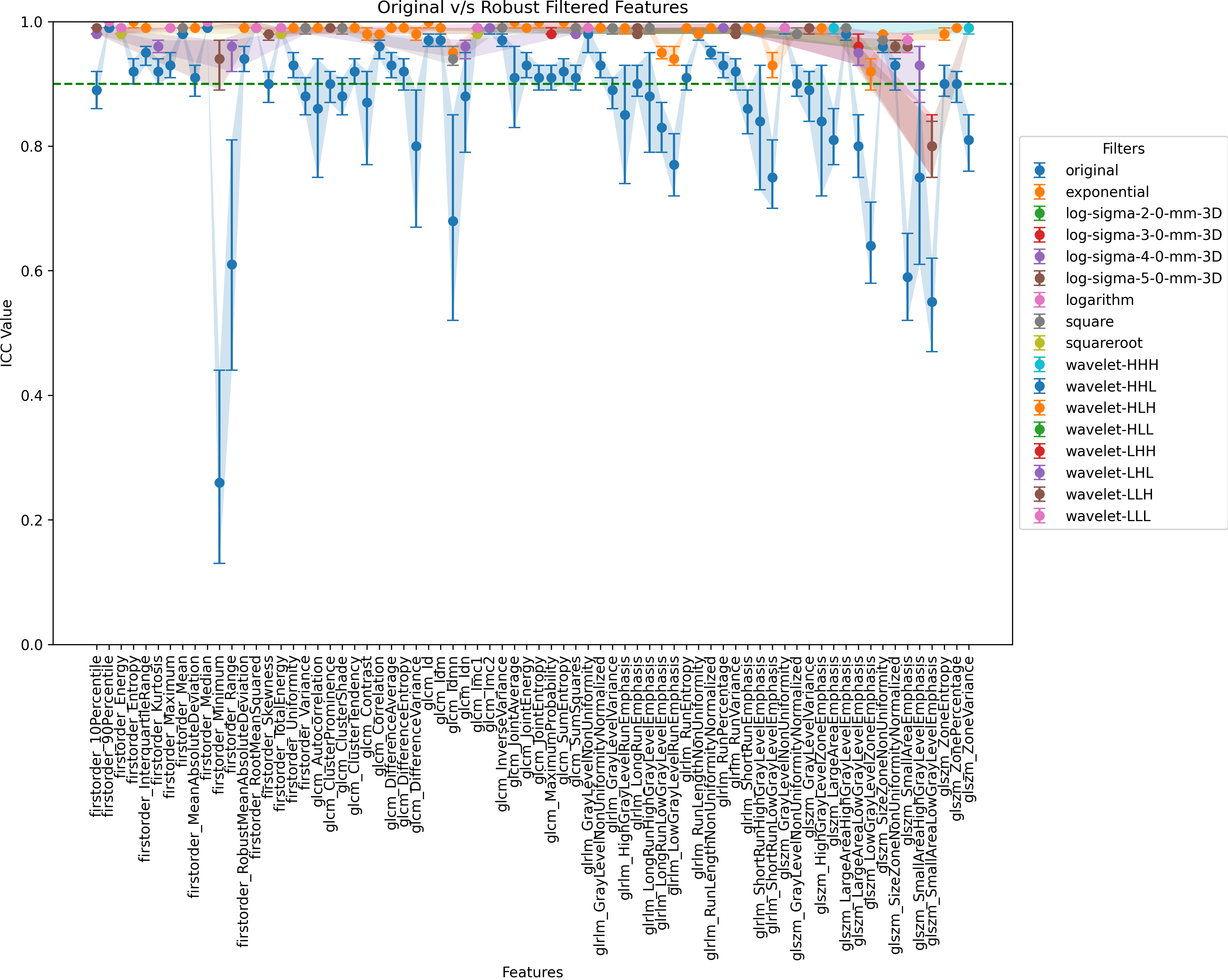

Supplement: Supplementary file 1 [file jpm-13-01172-s001.zip › overlap_plots/adc/inout_plane_systematic.png]

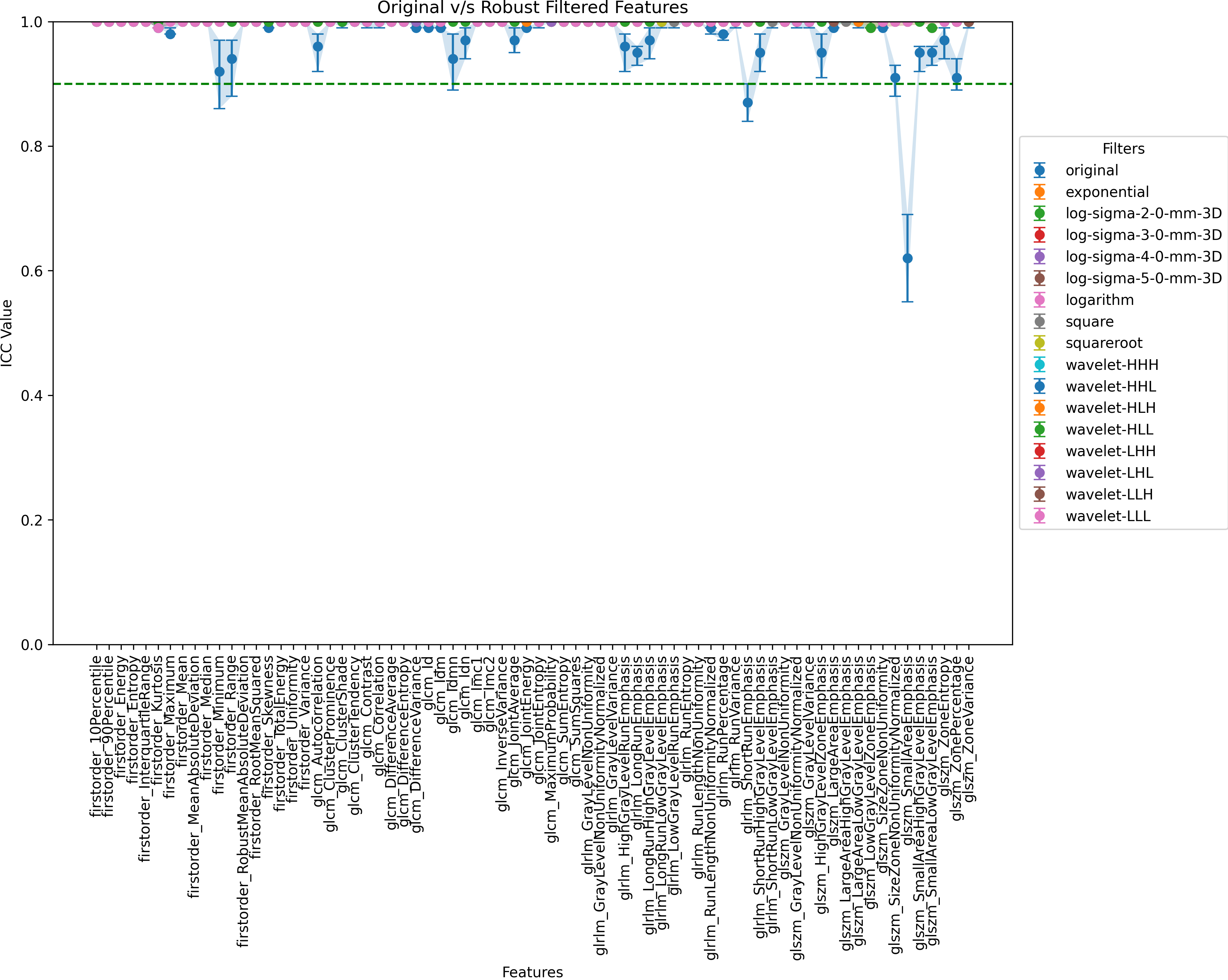

Supplement: Supplementary file 1 [file jpm-13-01172-s001.zip › overlap_plots/adc/out_plane.png]

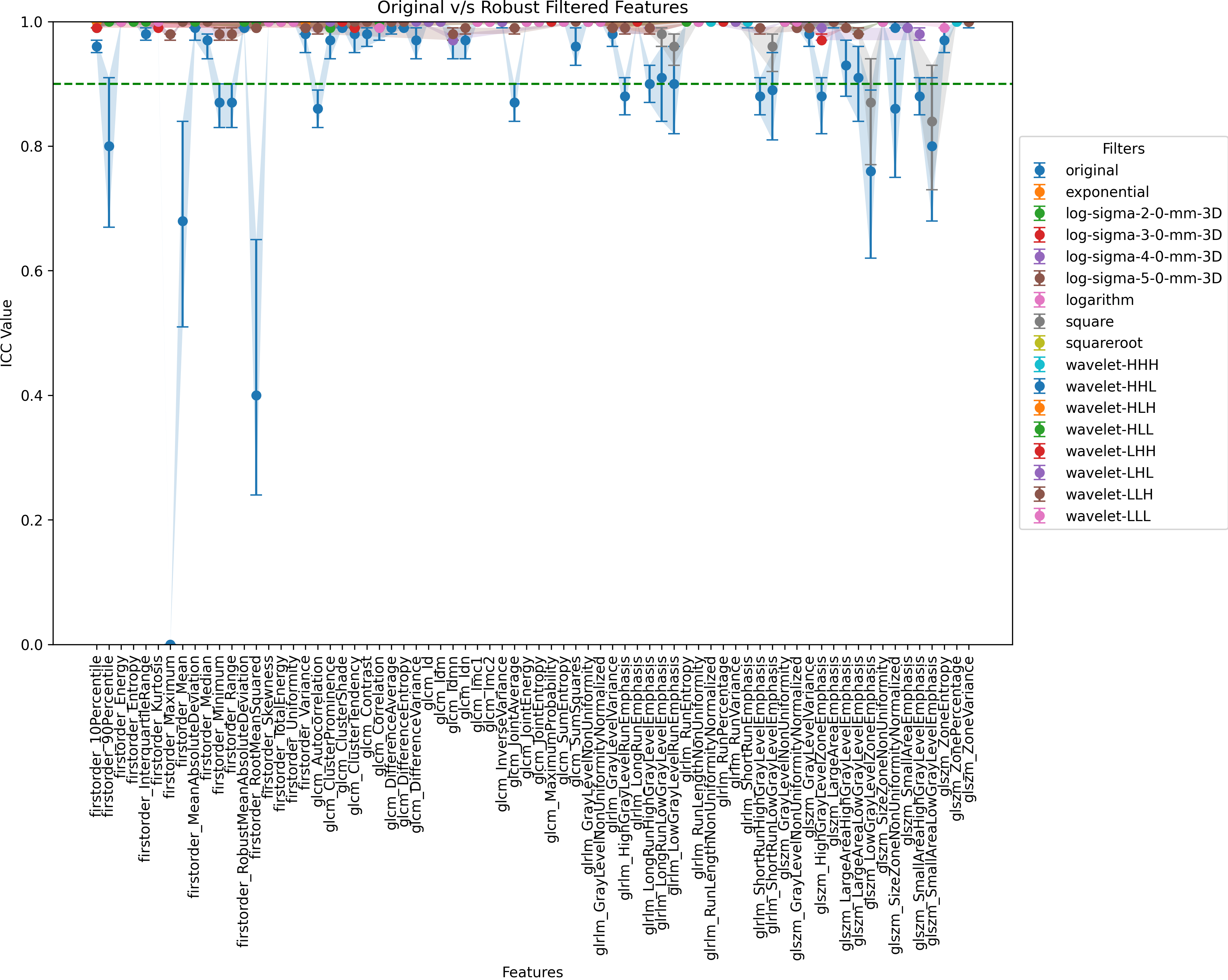

Supplement: Supplementary file 1 [file jpm-13-01172-s001.zip › overlap_plots/sub_win/in_plane_random.png]

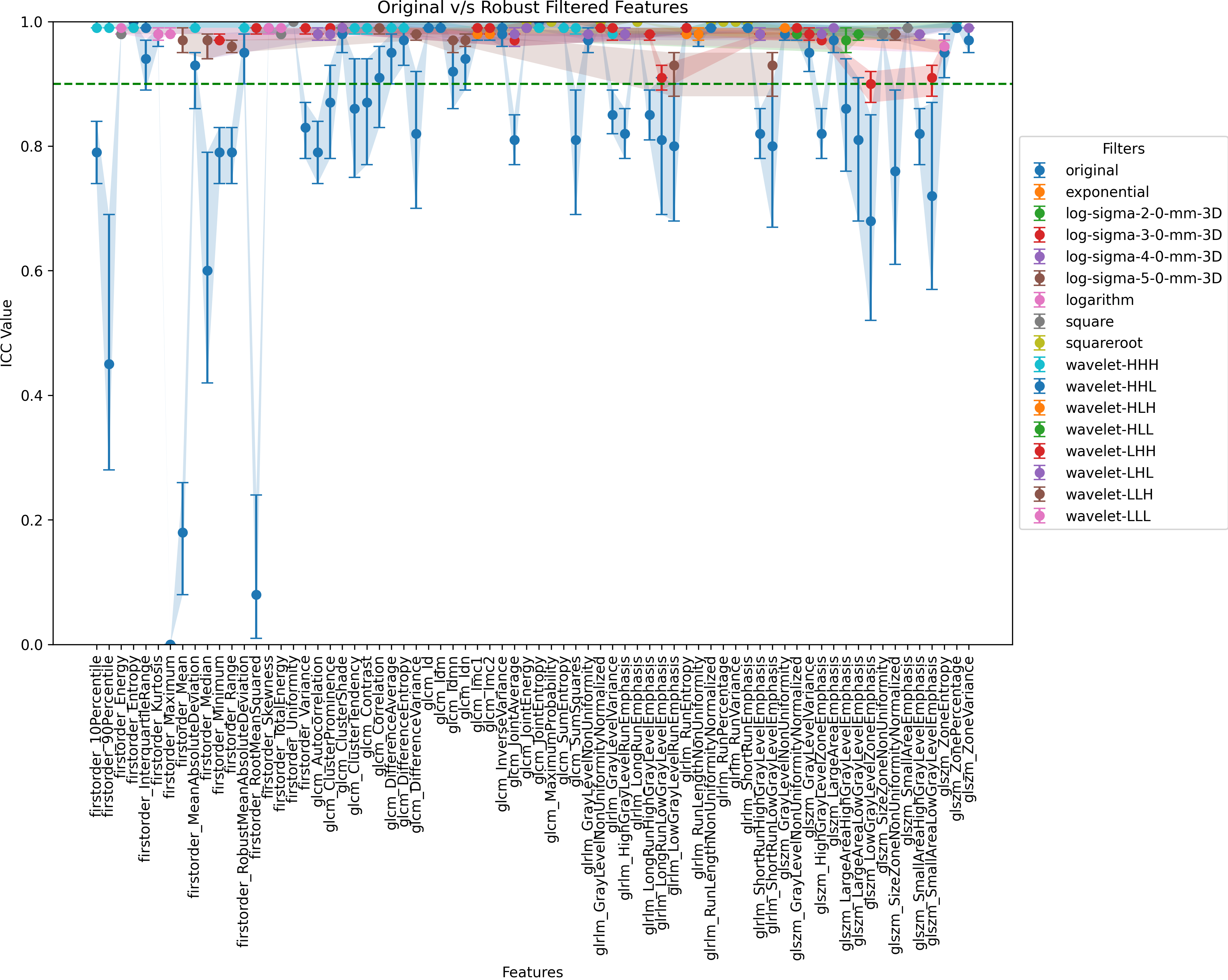

Supplement: Supplementary file 1 [file jpm-13-01172-s001.zip › overlap_plots/sub_win/in_plane_systematic.png]

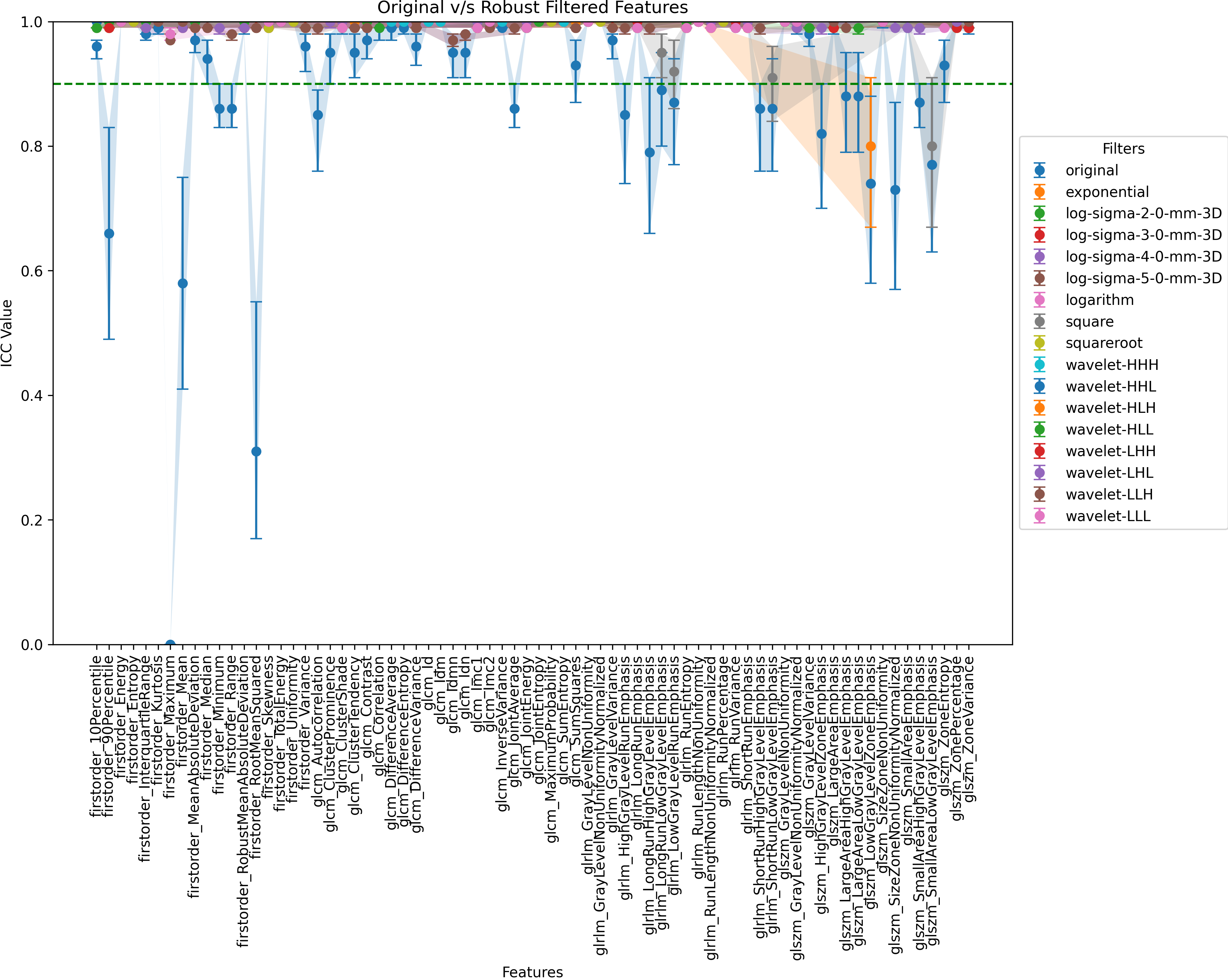

Supplement: Supplementary file 1 [file jpm-13-01172-s001.zip › overlap_plots/sub_win/inout_plane_random.png]

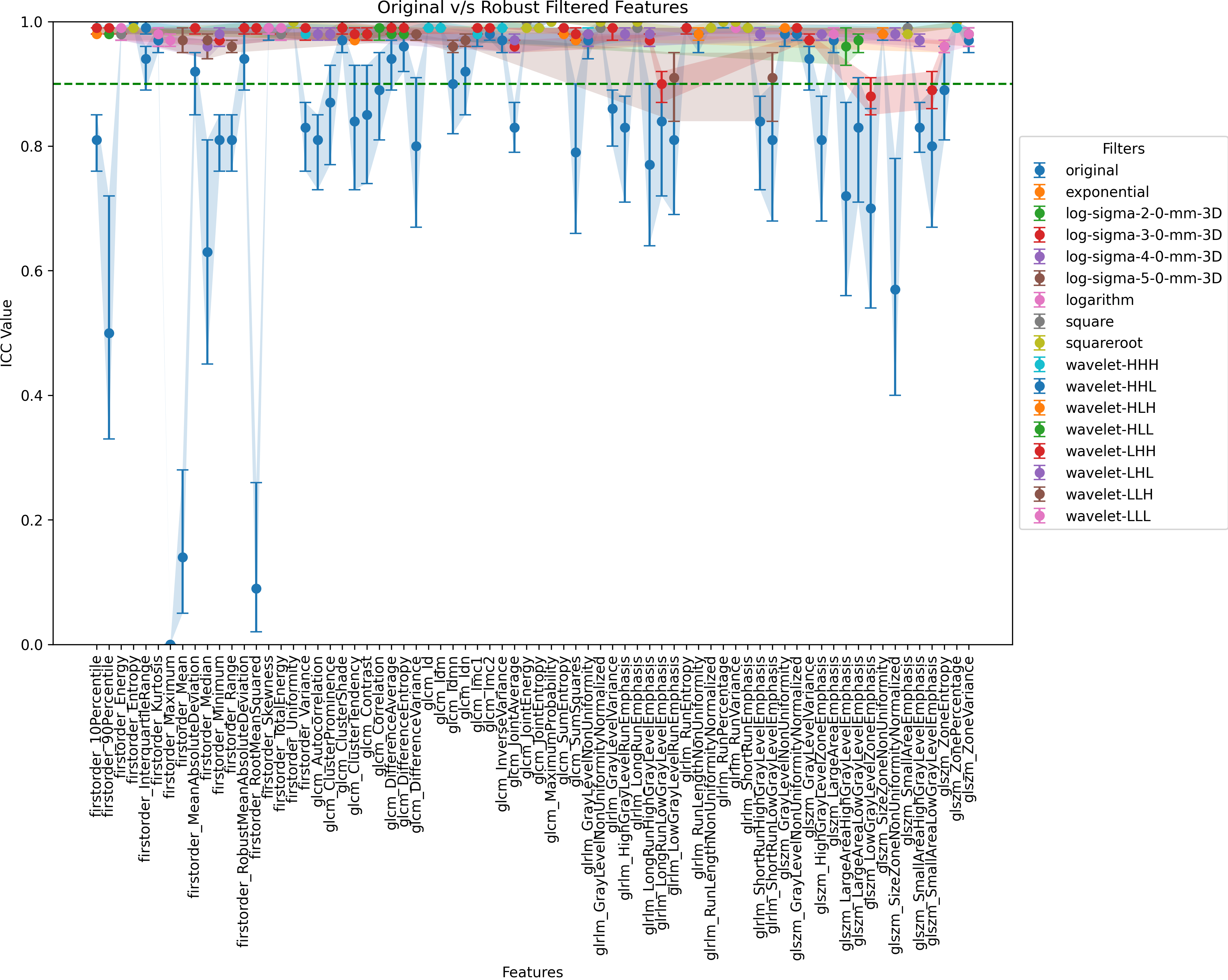

Supplement: Supplementary file 1 [file jpm-13-01172-s001.zip › overlap_plots/sub_win/inout_plane_systematic.png]

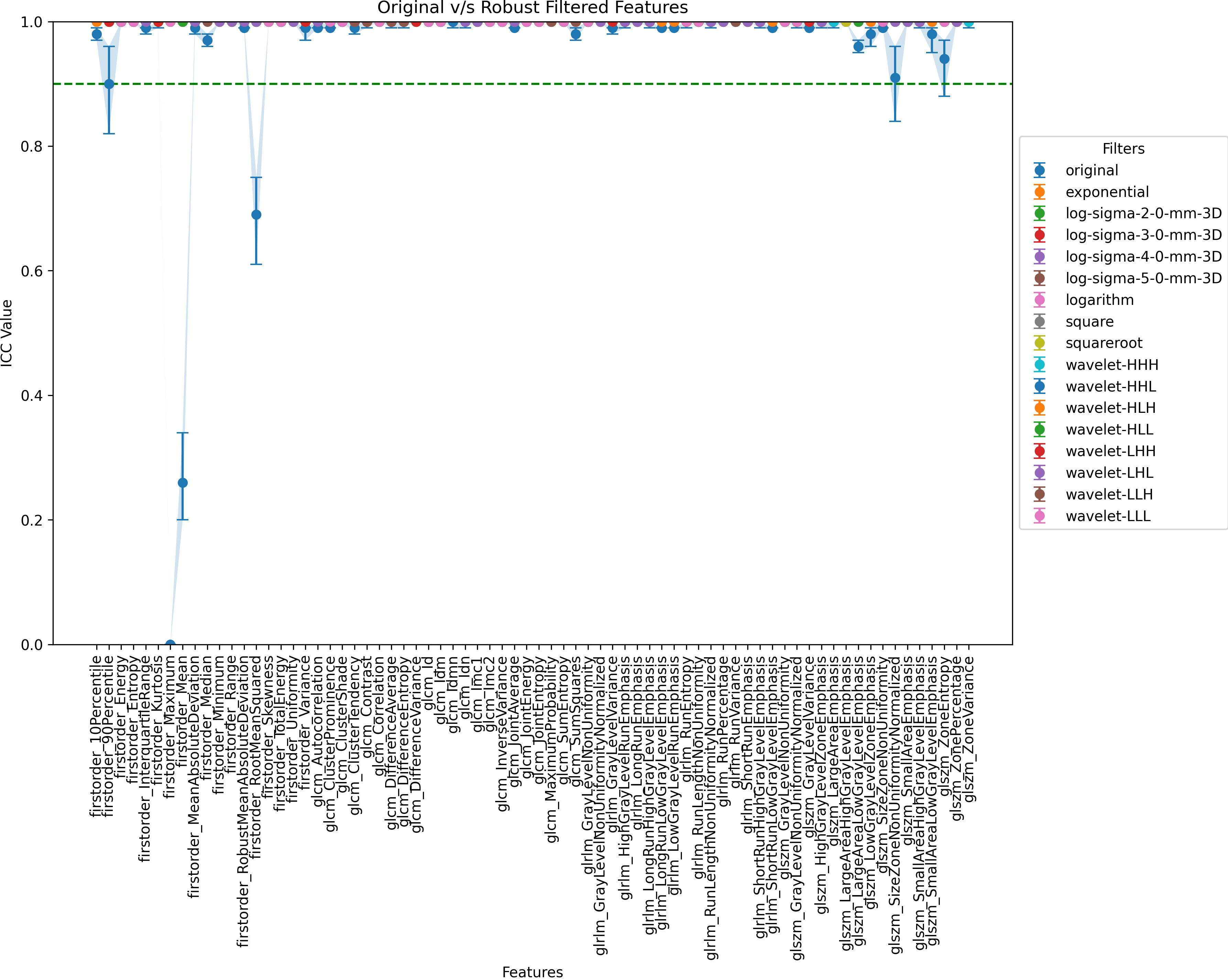

Supplement: Supplementary file 1 [file jpm-13-01172-s001.zip › overlap_plots/sub_win/out_plane.png]

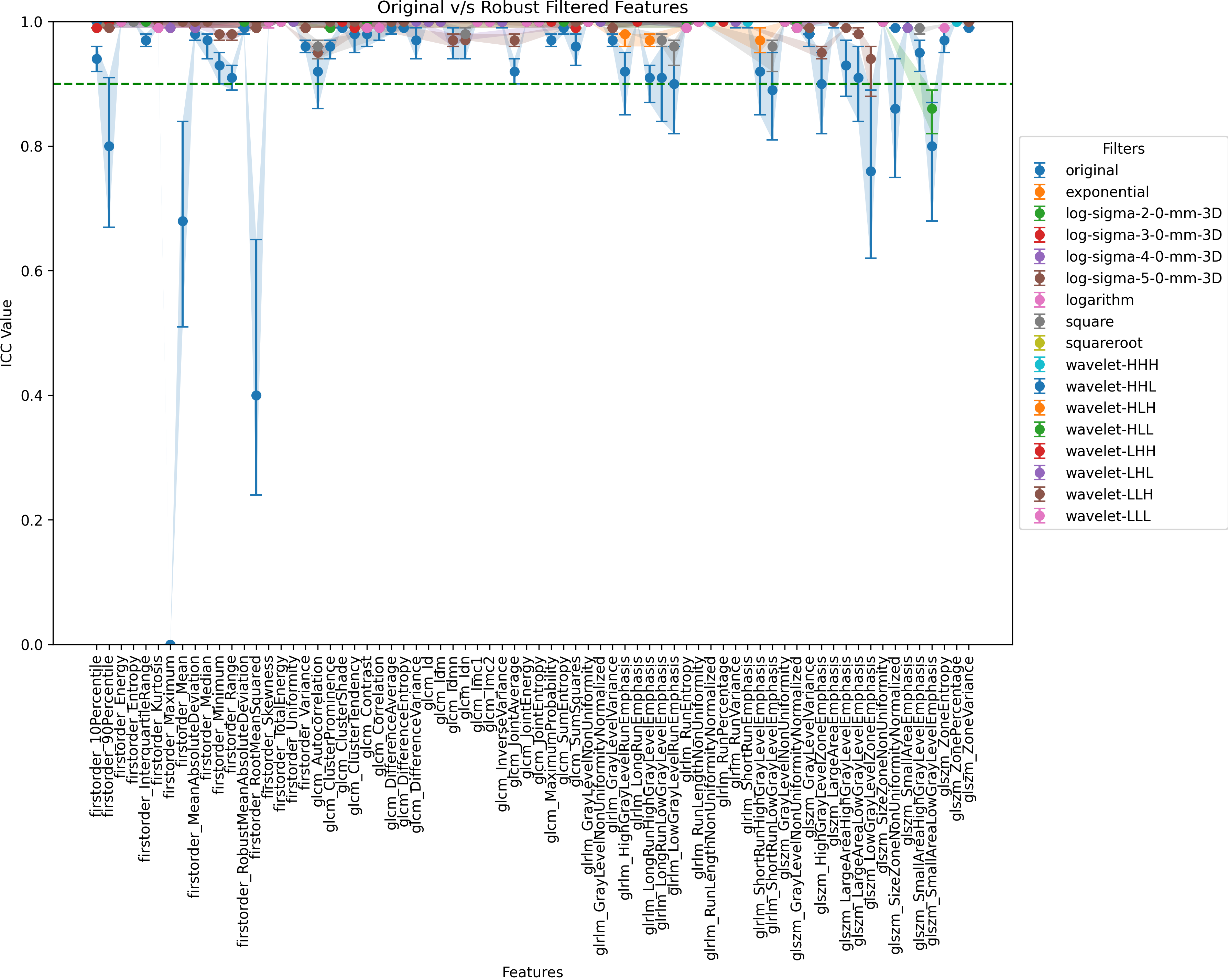

Supplement: Supplementary file 1 [file jpm-13-01172-s001.zip › overlap_plots/sub_wout/in_plane_random.png]

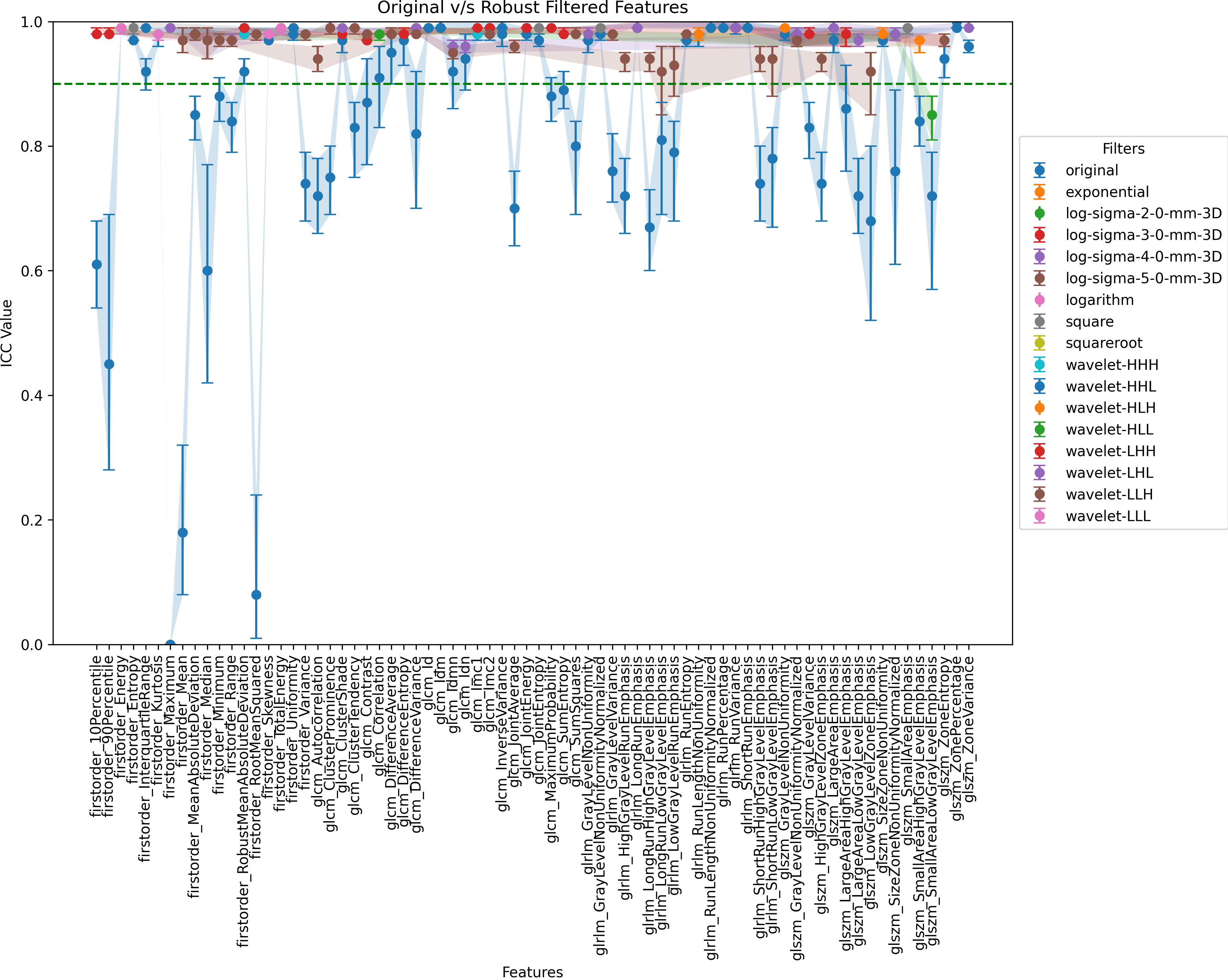

Supplement: Supplementary file 1 [file jpm-13-01172-s001.zip › overlap_plots/sub_wout/in_plane_systematic.png]

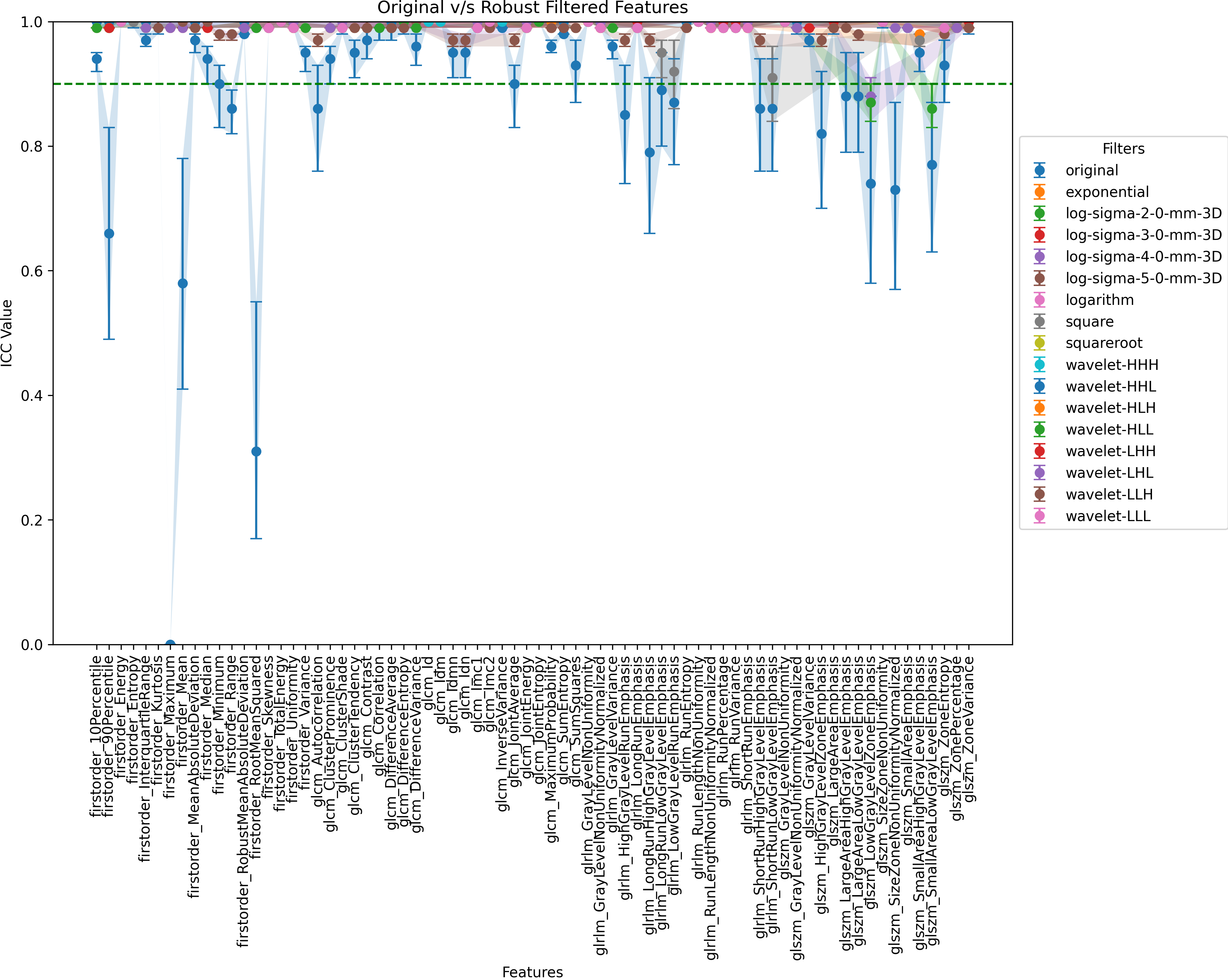

Supplement: Supplementary file 1 [file jpm-13-01172-s001.zip › overlap_plots/sub_wout/inout_plane_random.png]

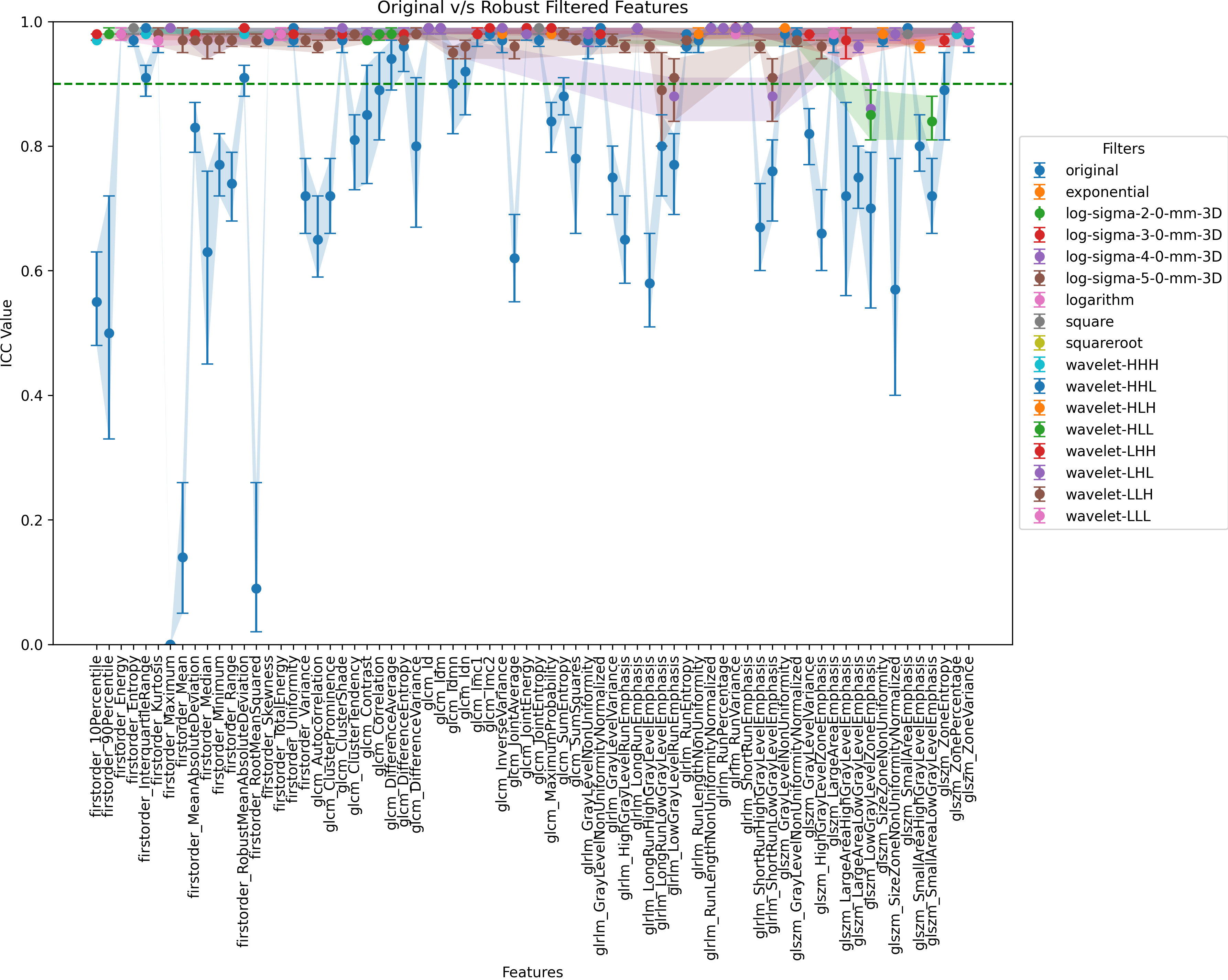

Supplement: Supplementary file 1 [file jpm-13-01172-s001.zip › overlap_plots/sub_wout/inout_plane_systematic.png]

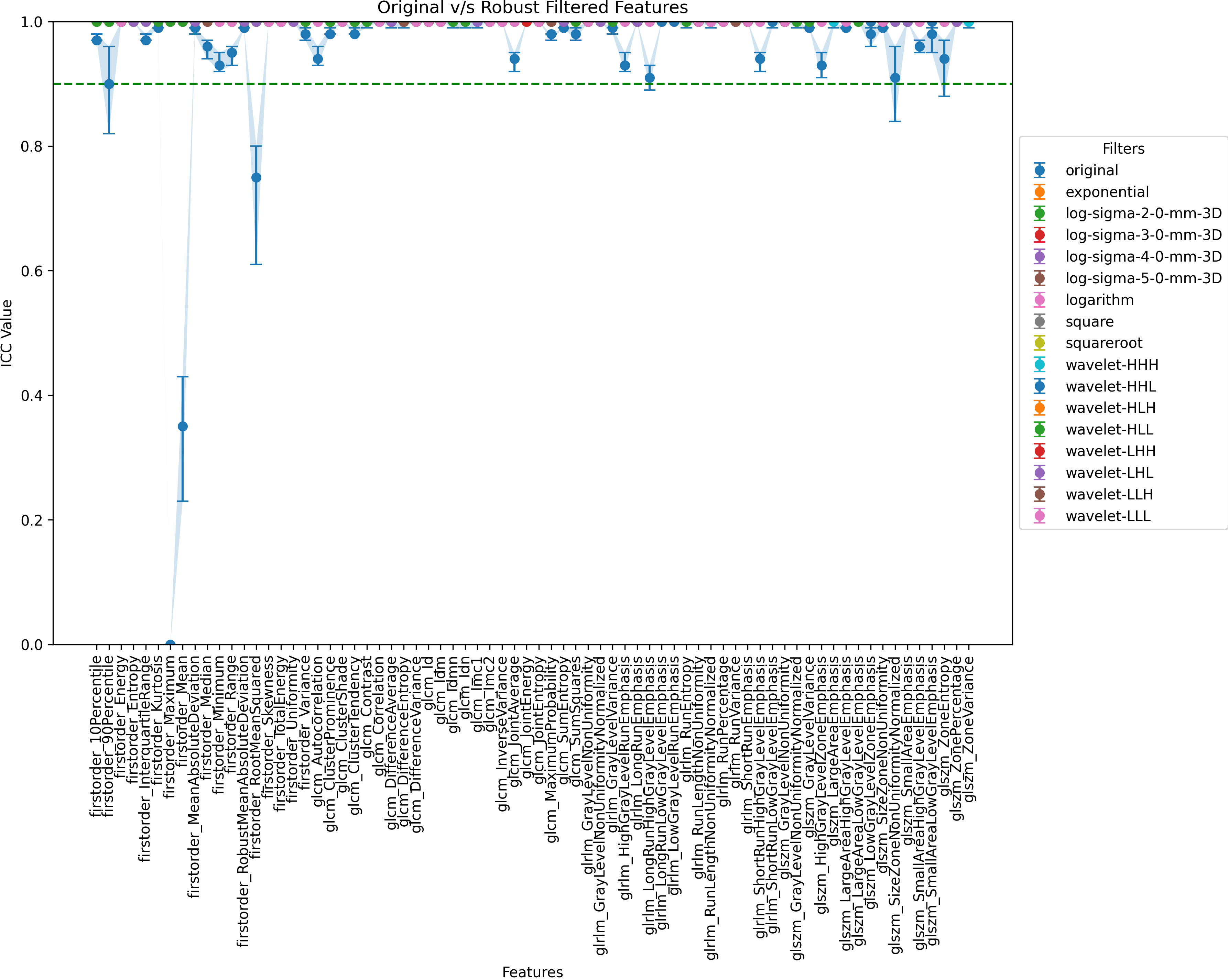

Supplement: Supplementary file 1 [file jpm-13-01172-s001.zip › overlap_plots/sub_wout/out_plane.png]

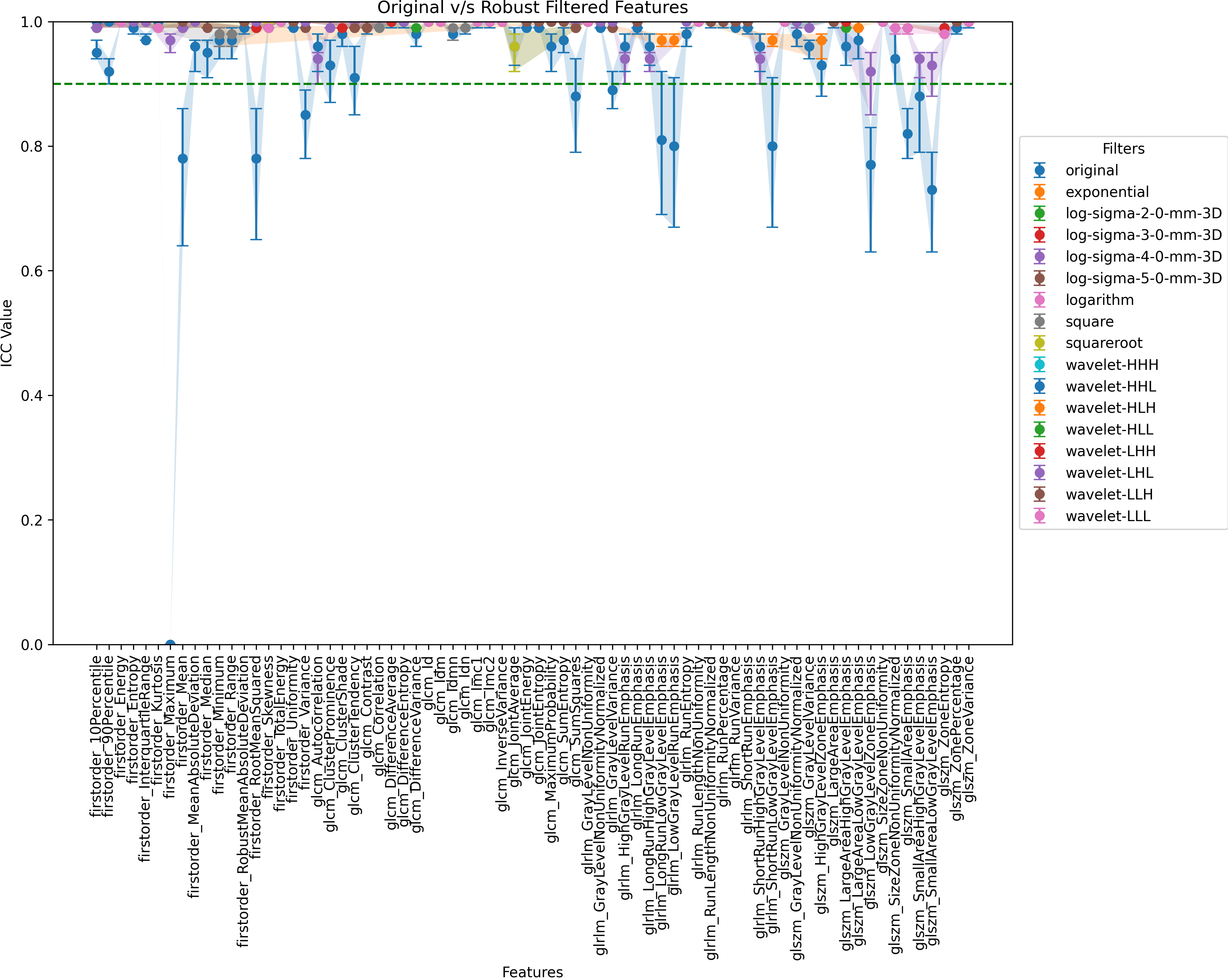

Supplement: Supplementary file 1 [file jpm-13-01172-s001.zip › overlap_plots/t2w/in_plane_random.png]

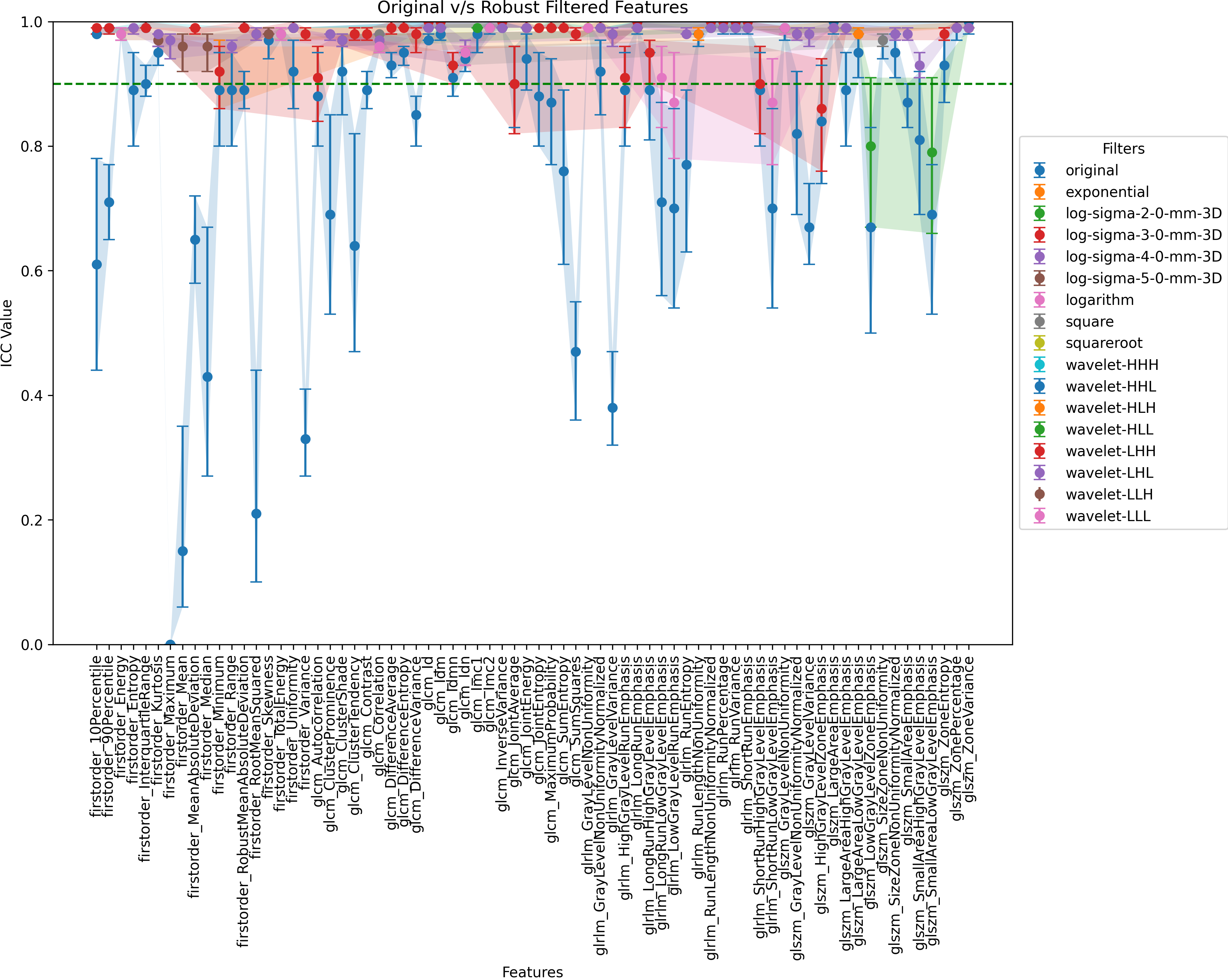

Supplement: Supplementary file 1 [file jpm-13-01172-s001.zip › overlap_plots/t2w/in_plane_systematic.png]

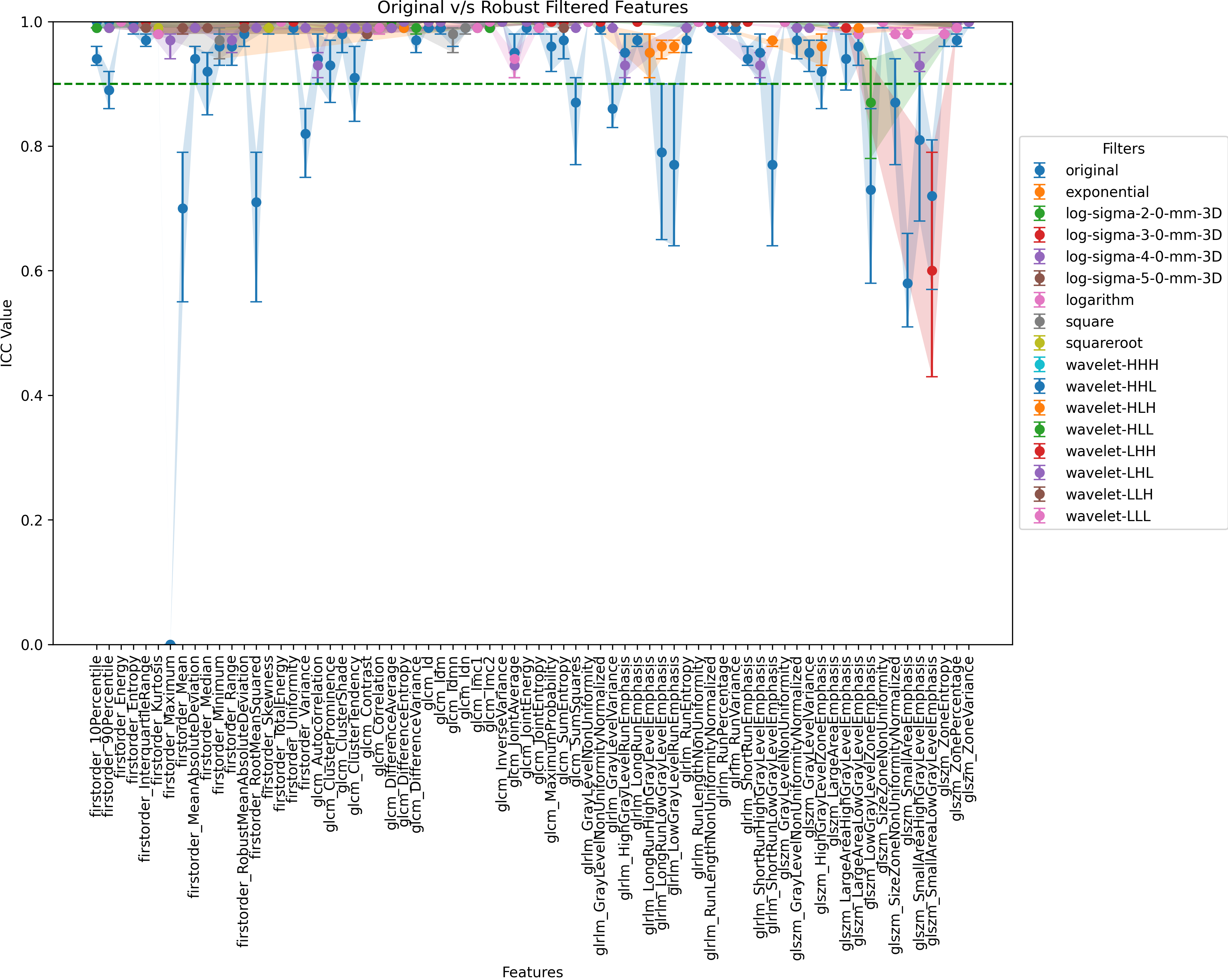

Supplement: Supplementary file 1 [file jpm-13-01172-s001.zip › overlap_plots/t2w/inout_plane_random.png]

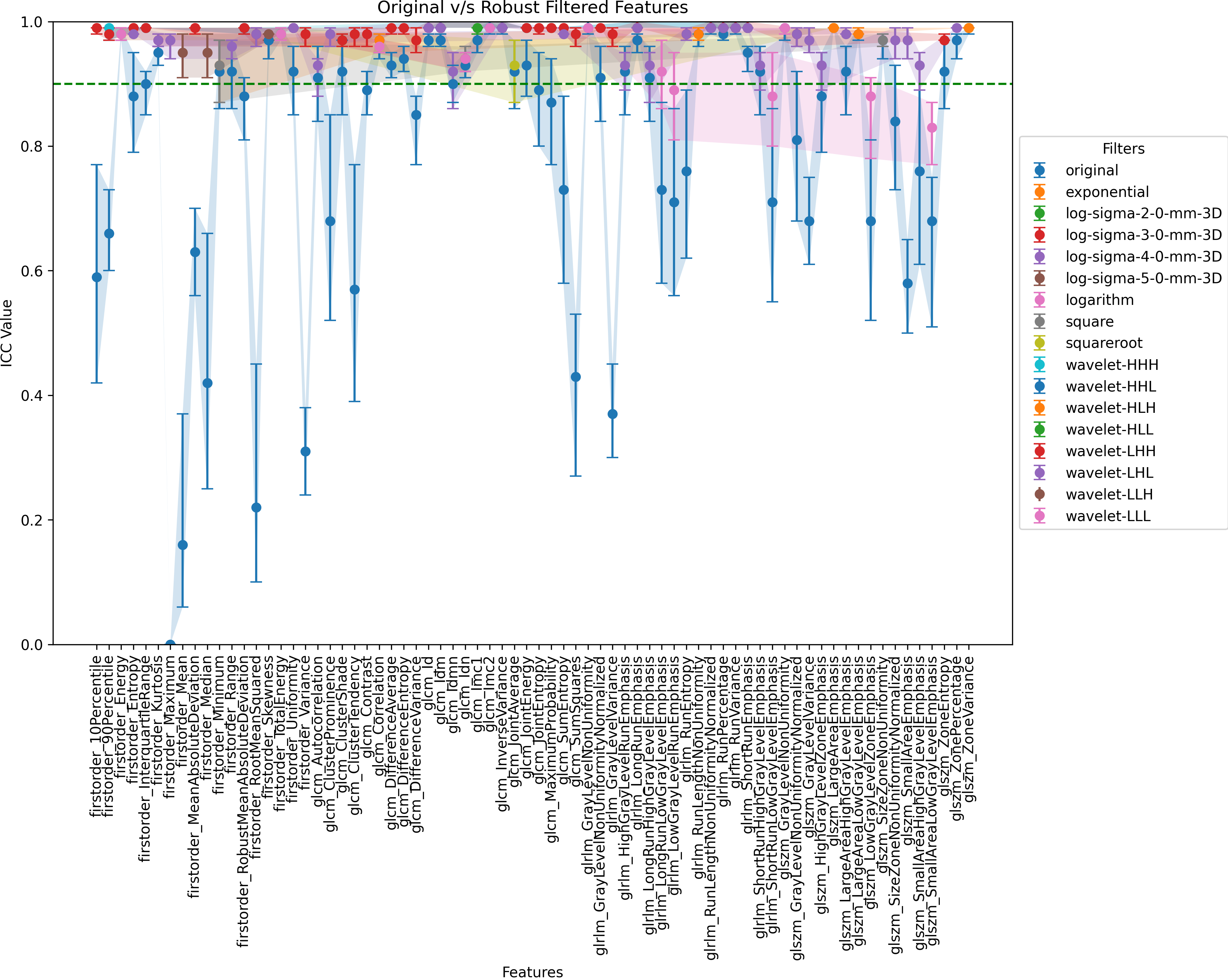

Supplement: Supplementary file 1 [file jpm-13-01172-s001.zip › overlap_plots/t2w/inout_plane_systematic.png]

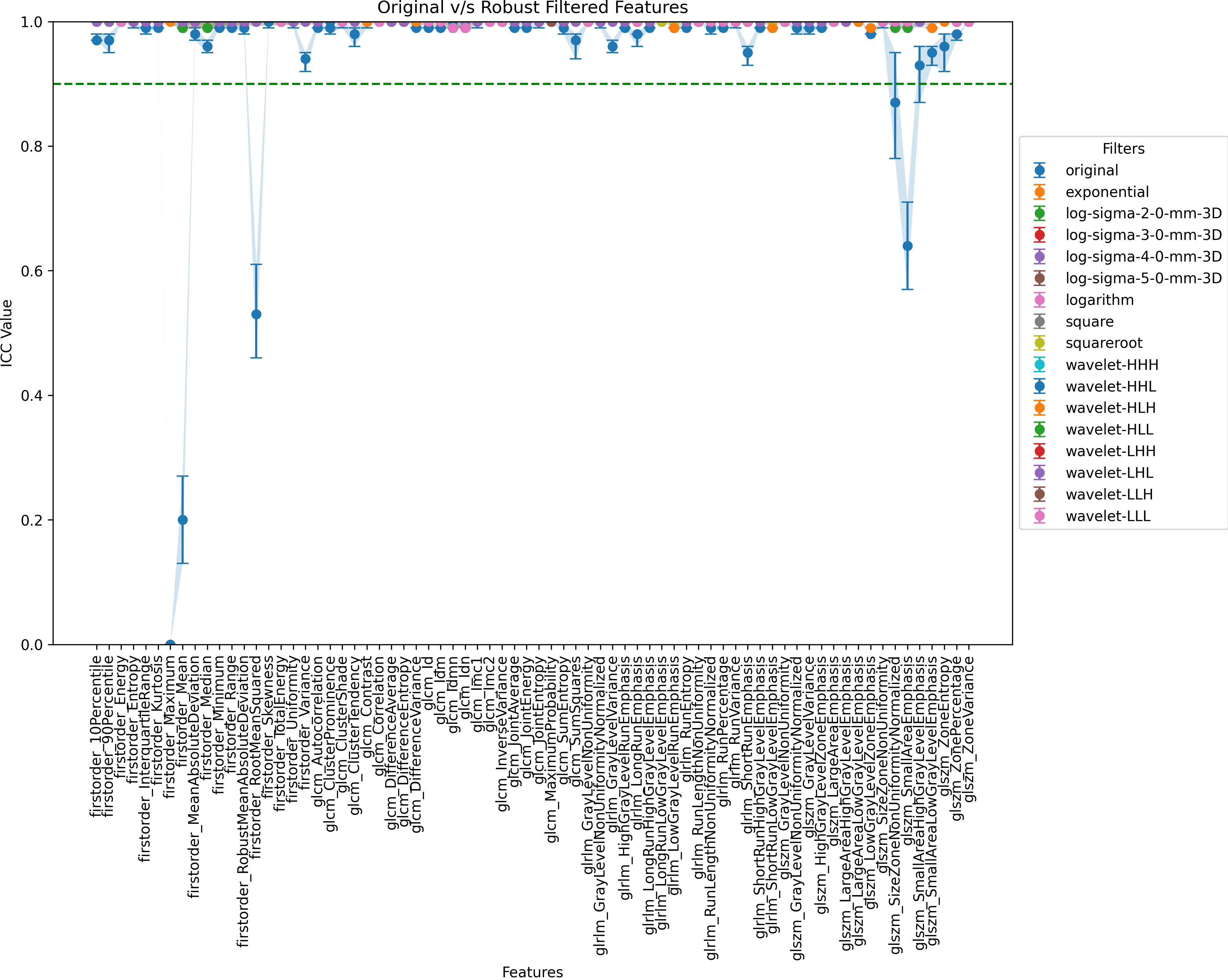

Supplement: Supplementary file 1 [file jpm-13-01172-s001.zip › overlap_plots/t2w/out_plane.png]

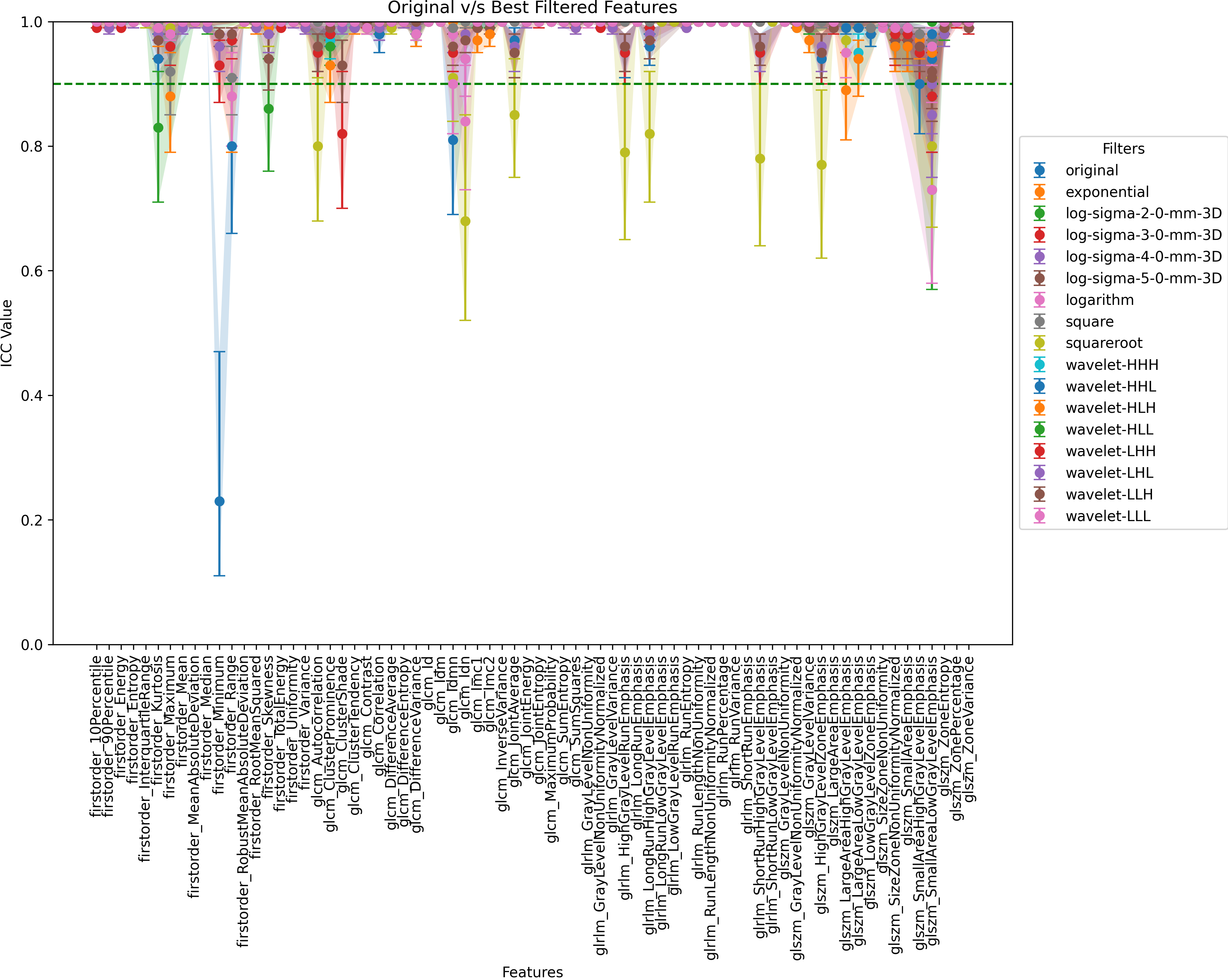

Supplement: Supplementary file 1 [file jpm-13-01172-s001.zip › plots/adc/in_plane_random_external.png]

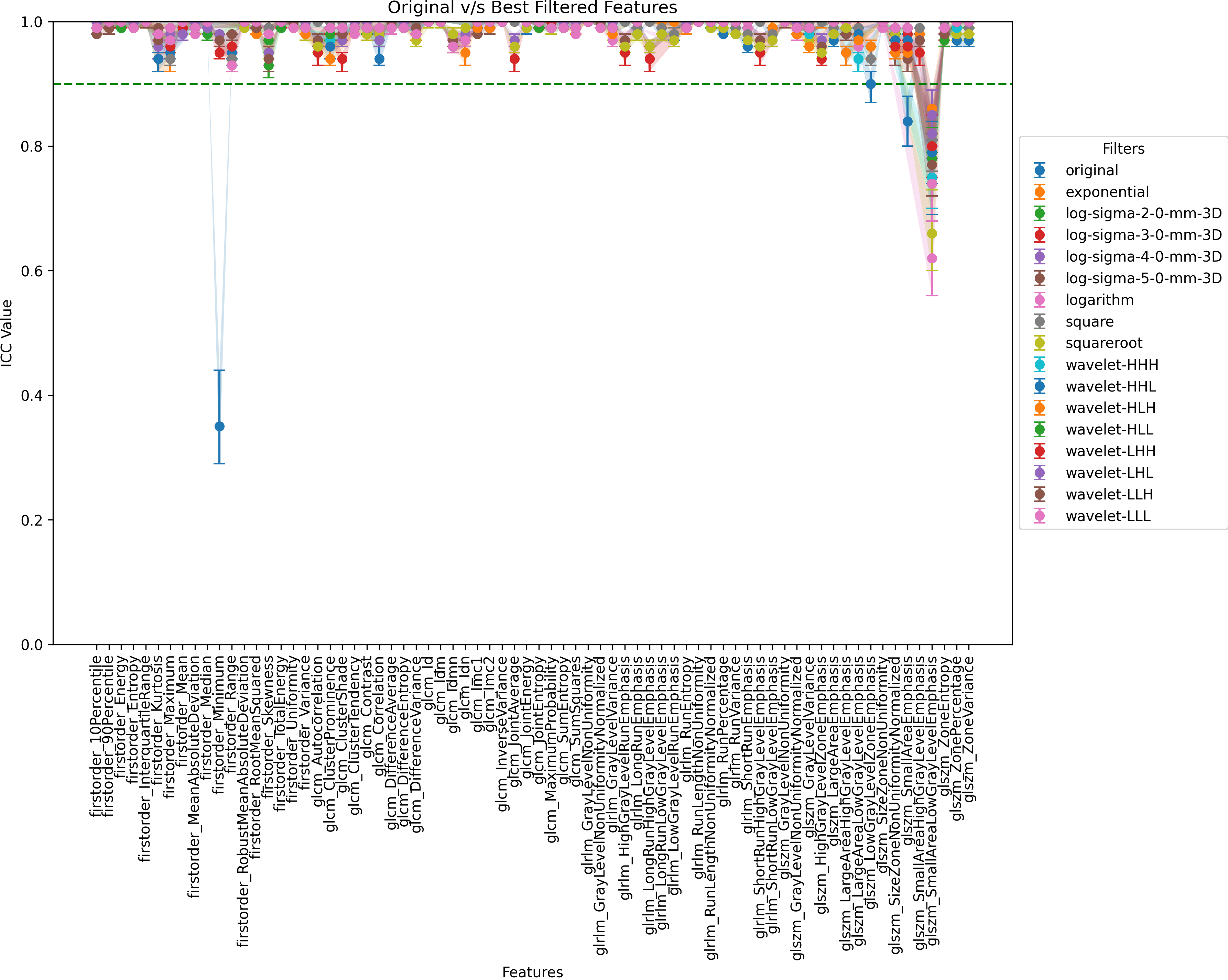

Supplement: Supplementary file 1 [file jpm-13-01172-s001.zip › plots/adc/in_plane_random_internal.png]

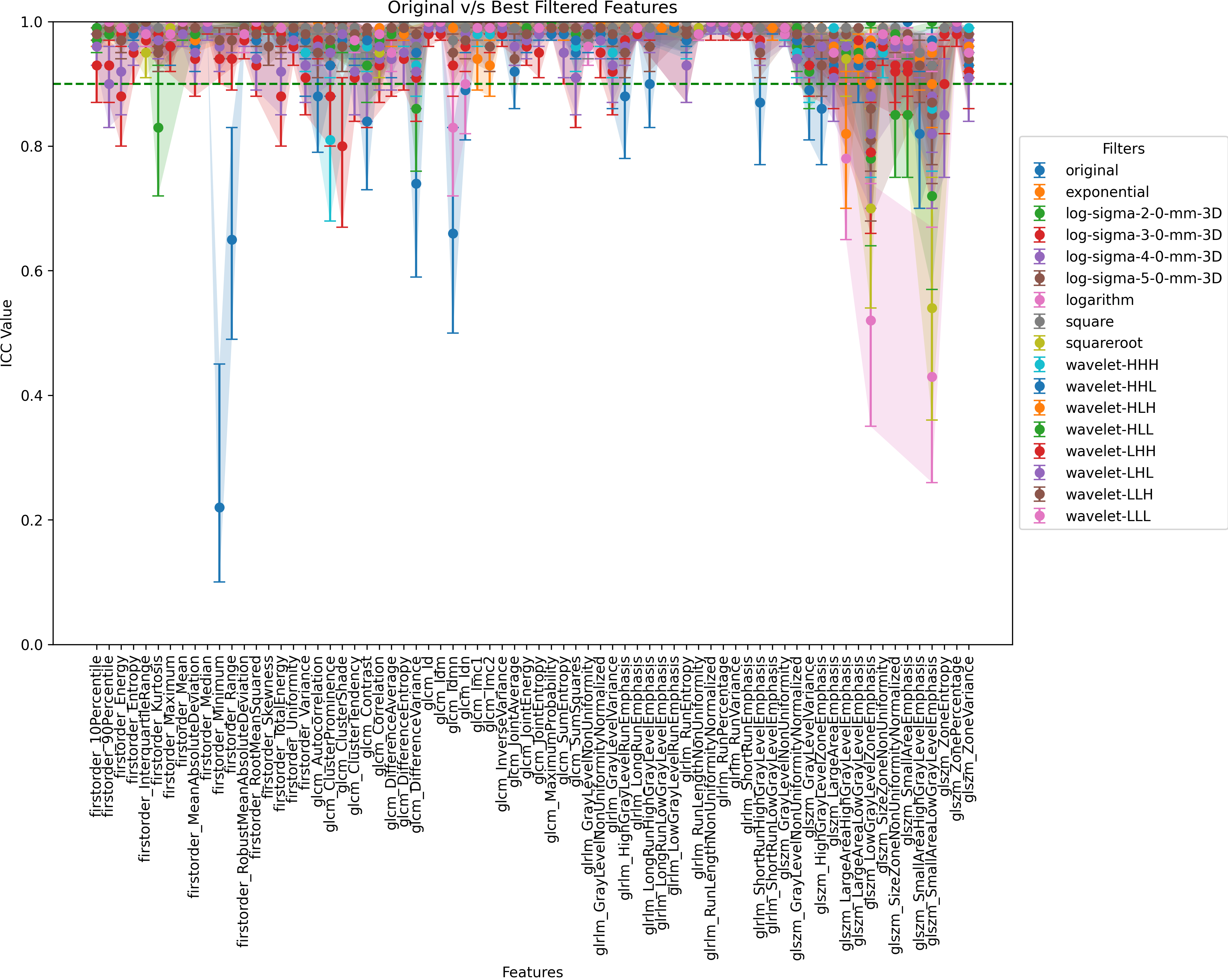

Supplement: Supplementary file 1 [file jpm-13-01172-s001.zip › plots/adc/in_plane_systematic_external.png]

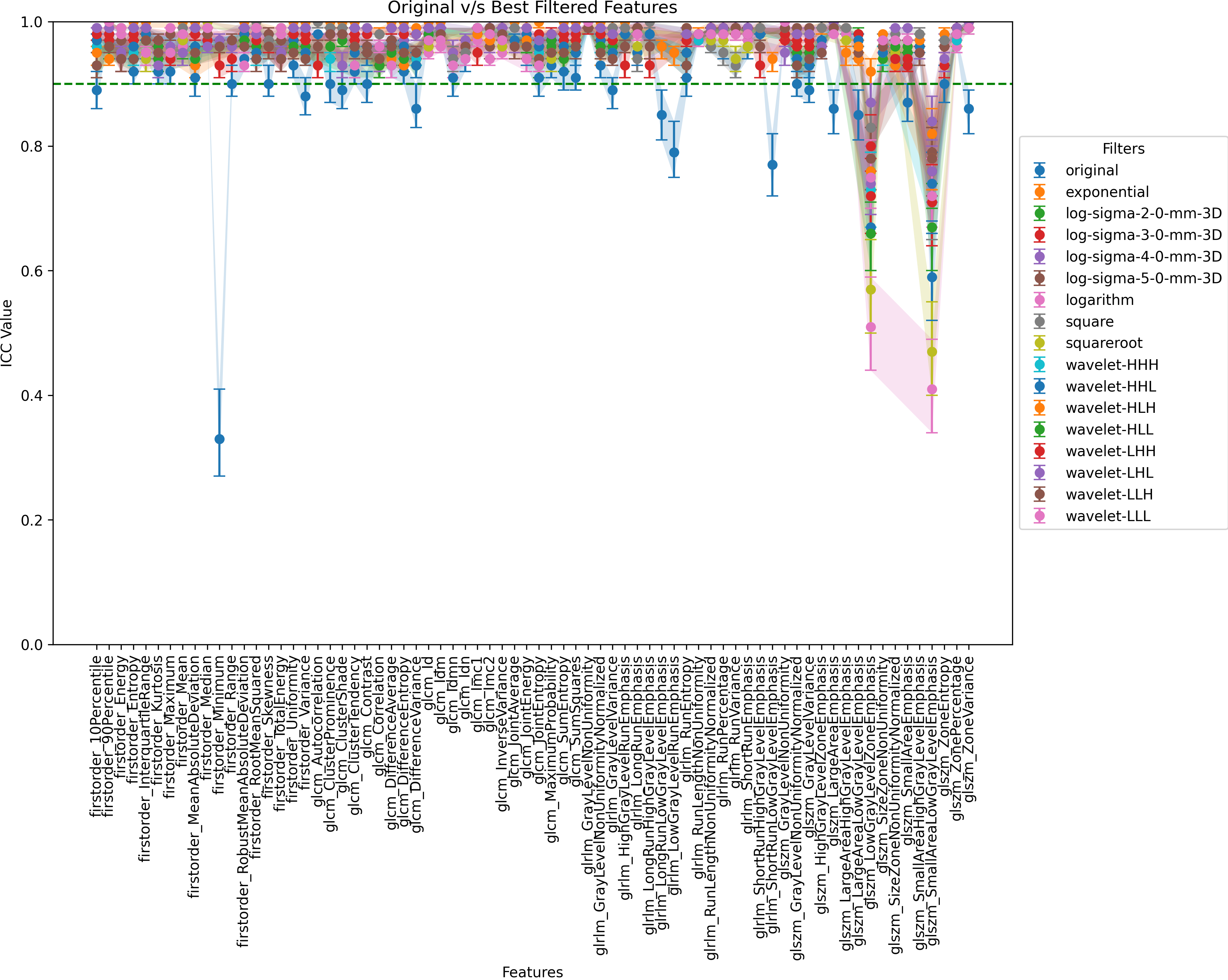

Supplement: Supplementary file 1 [file jpm-13-01172-s001.zip › plots/adc/in_plane_systematic_internal.png]

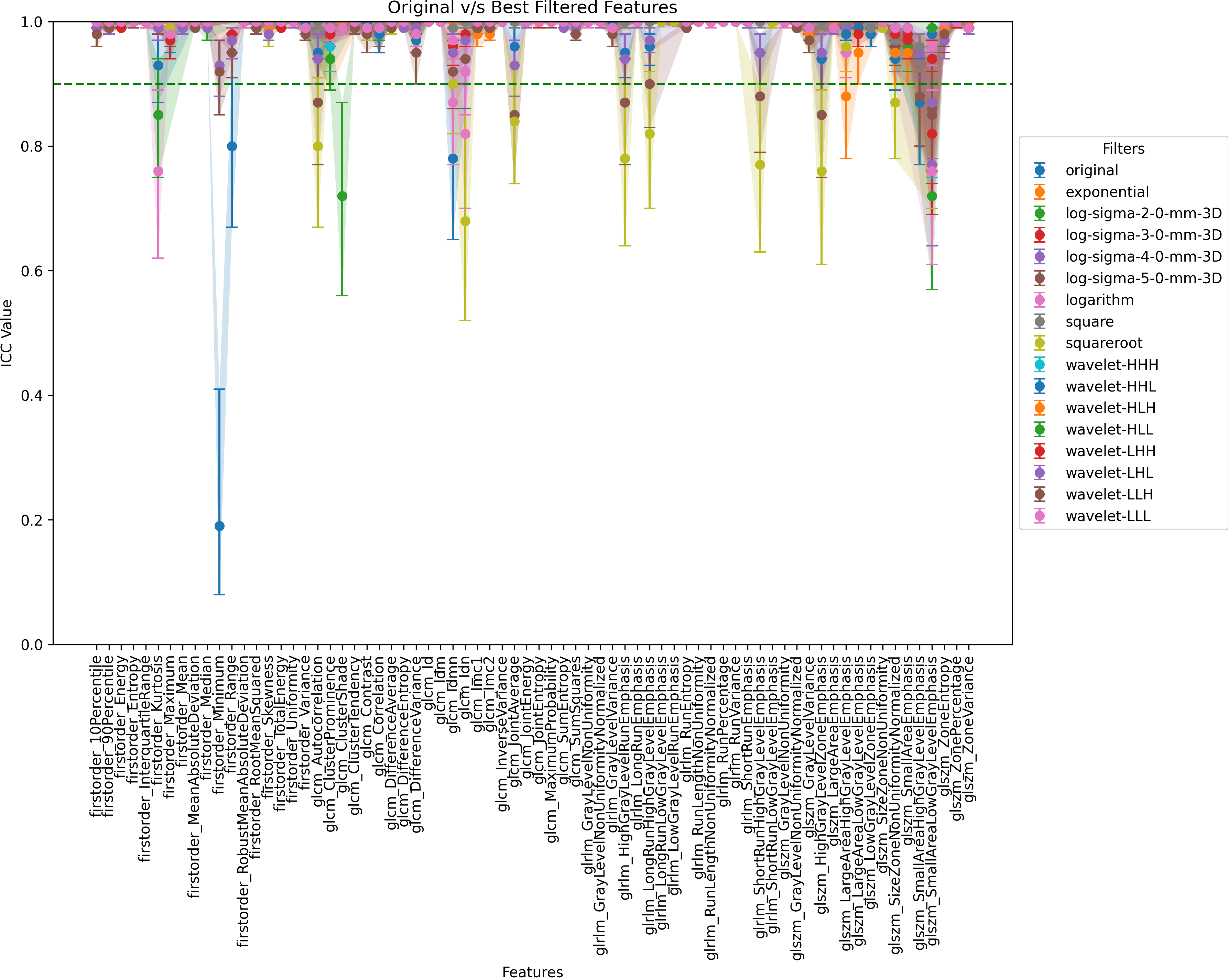

Supplement: Supplementary file 1 [file jpm-13-01172-s001.zip › plots/adc/inout_plane_random_external.png]

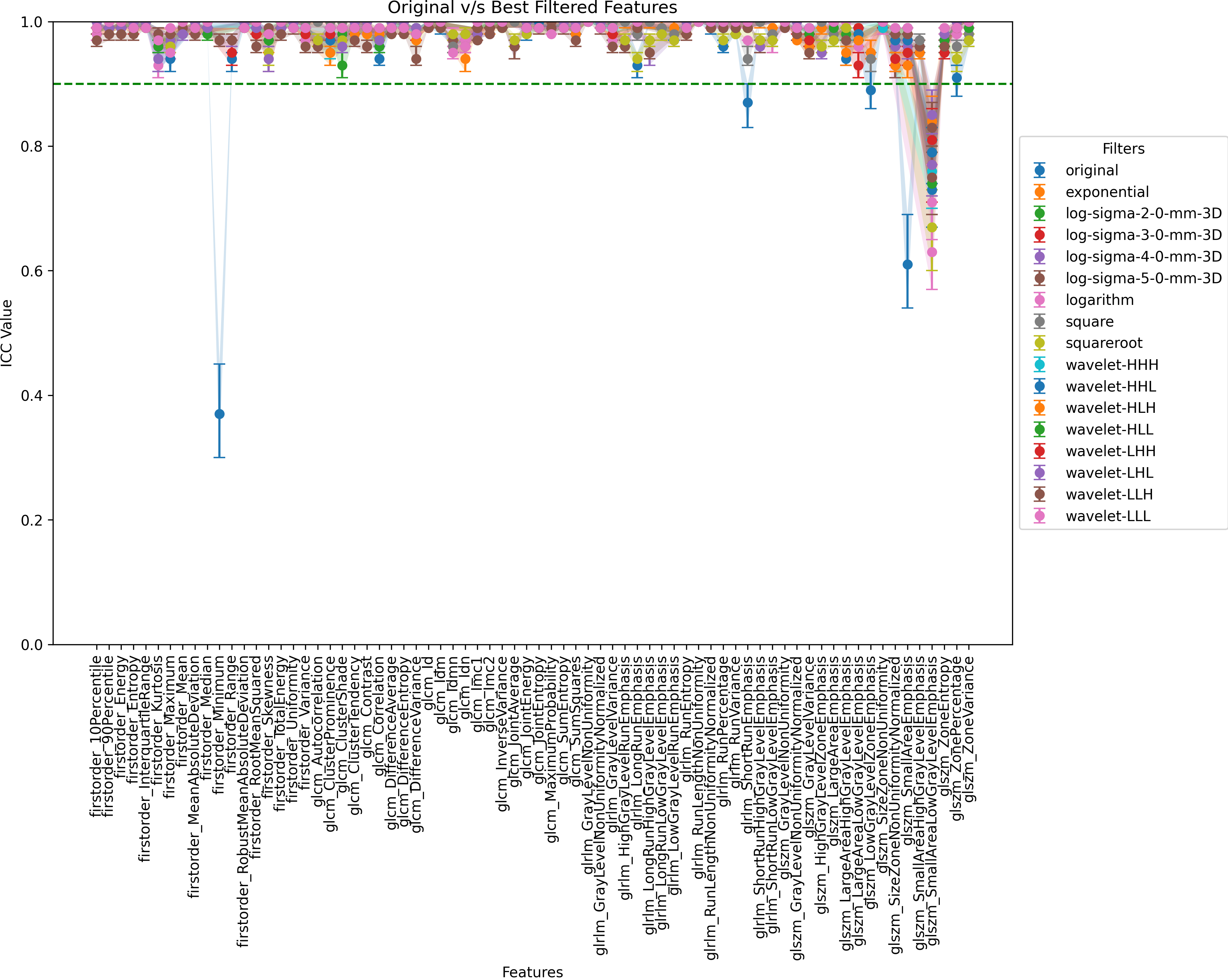

Supplement: Supplementary file 1 [file jpm-13-01172-s001.zip › plots/adc/inout_plane_random_internal.png]

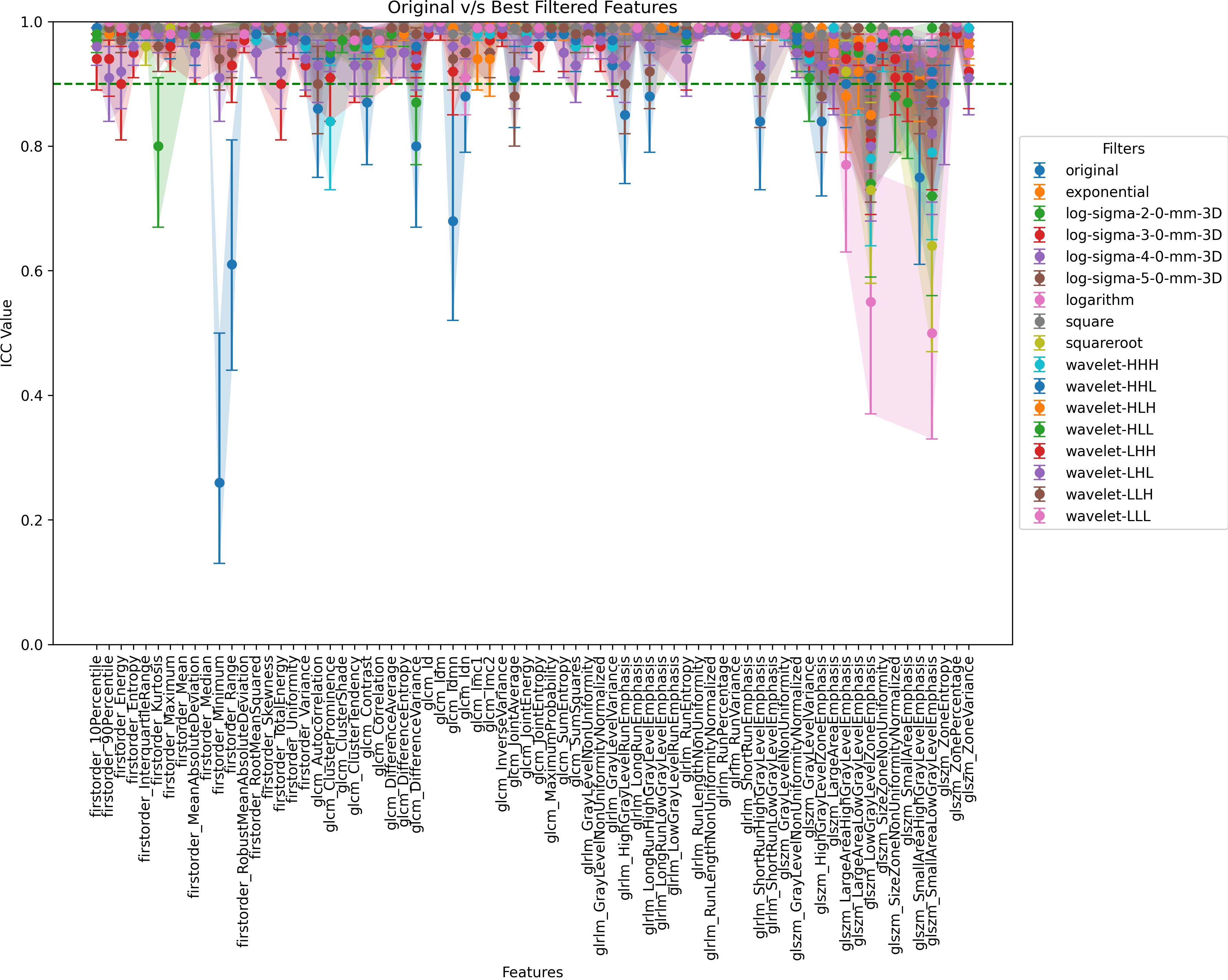

Supplement: Supplementary file 1 [file jpm-13-01172-s001.zip › plots/adc/inout_plane_systematic_external.png]

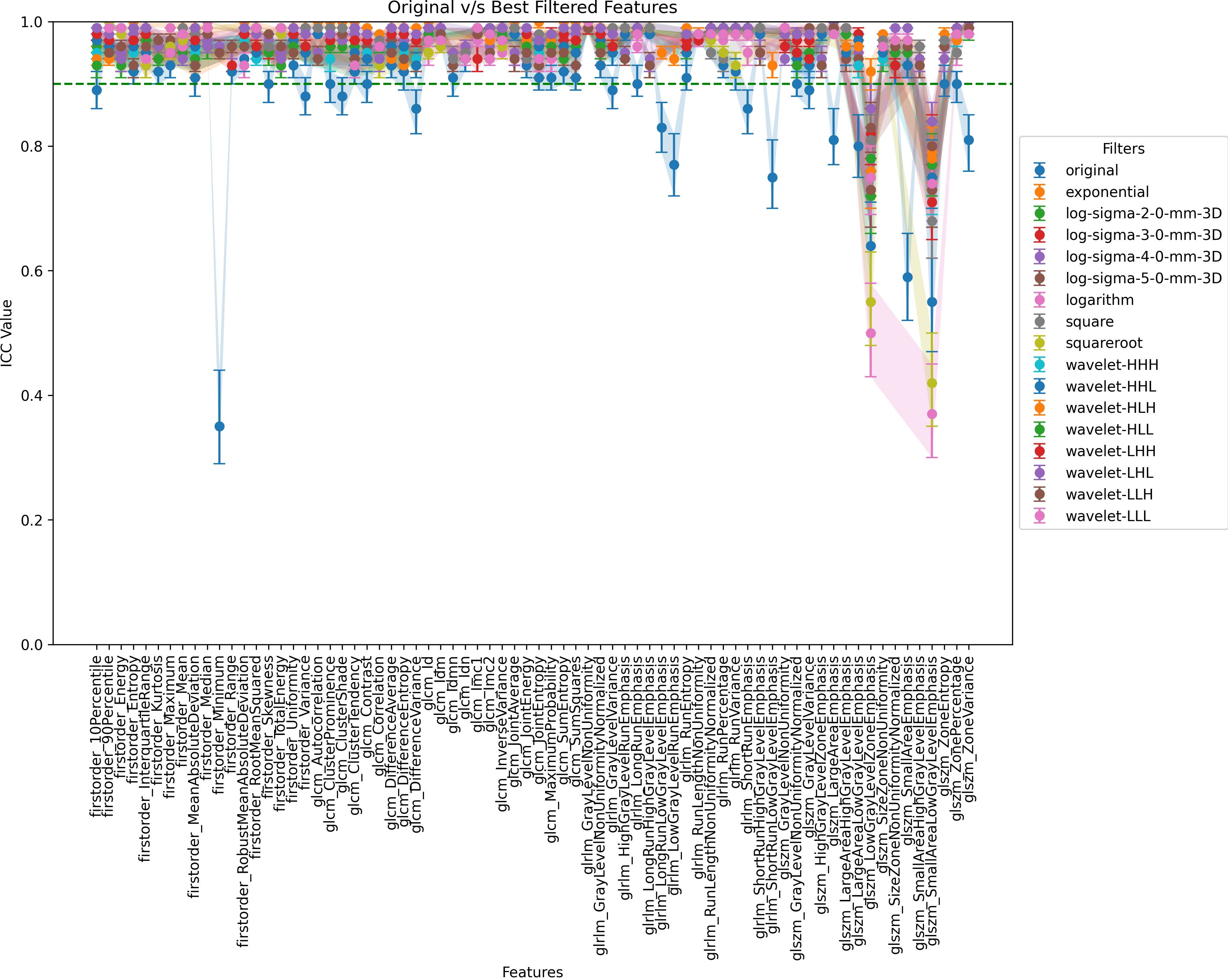

Supplement: Supplementary file 1 [file jpm-13-01172-s001.zip › plots/adc/inout_plane_systematic_internal.png]

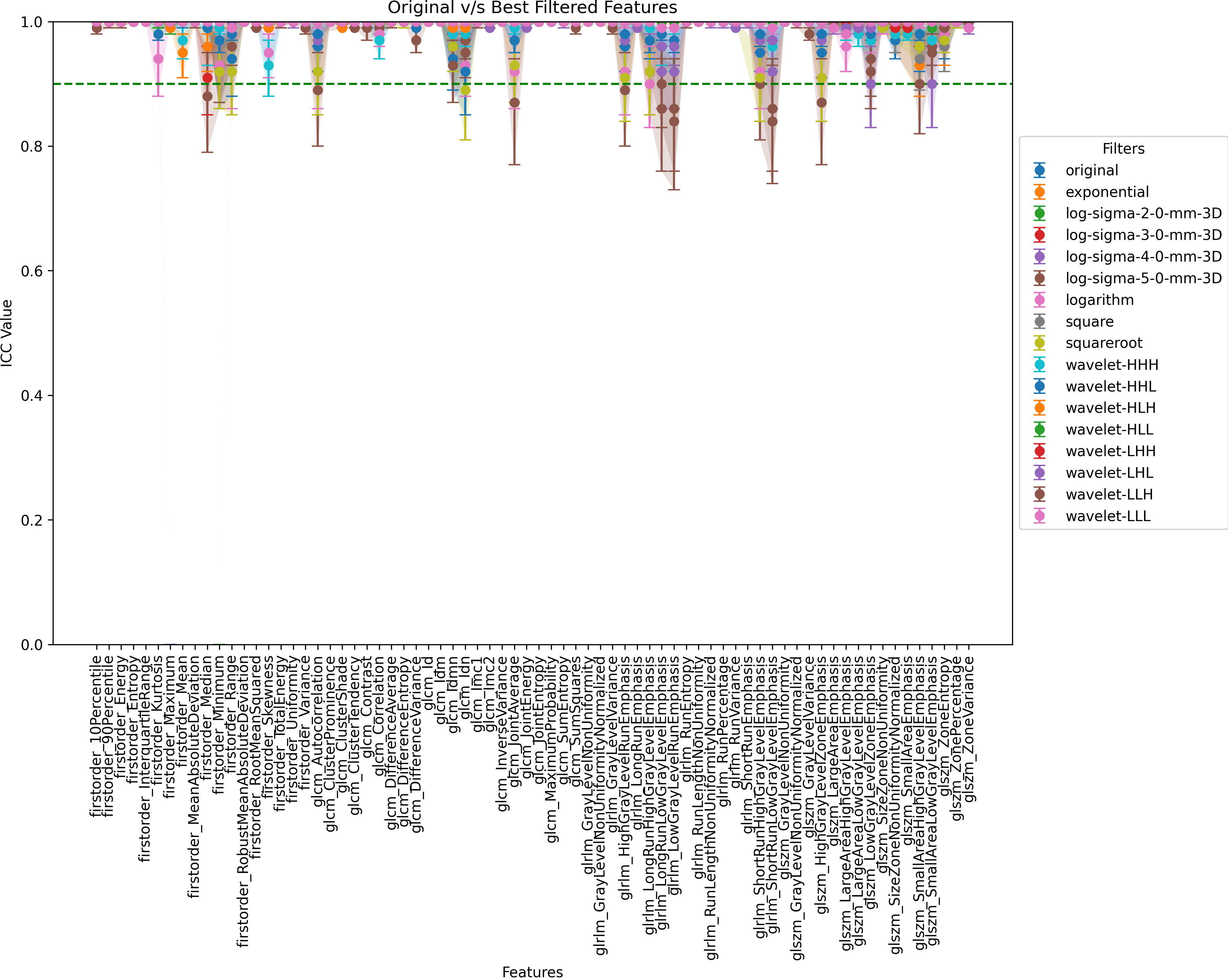

Supplement: Supplementary file 1 [file jpm-13-01172-s001.zip › plots/adc/out_plane_external.png]

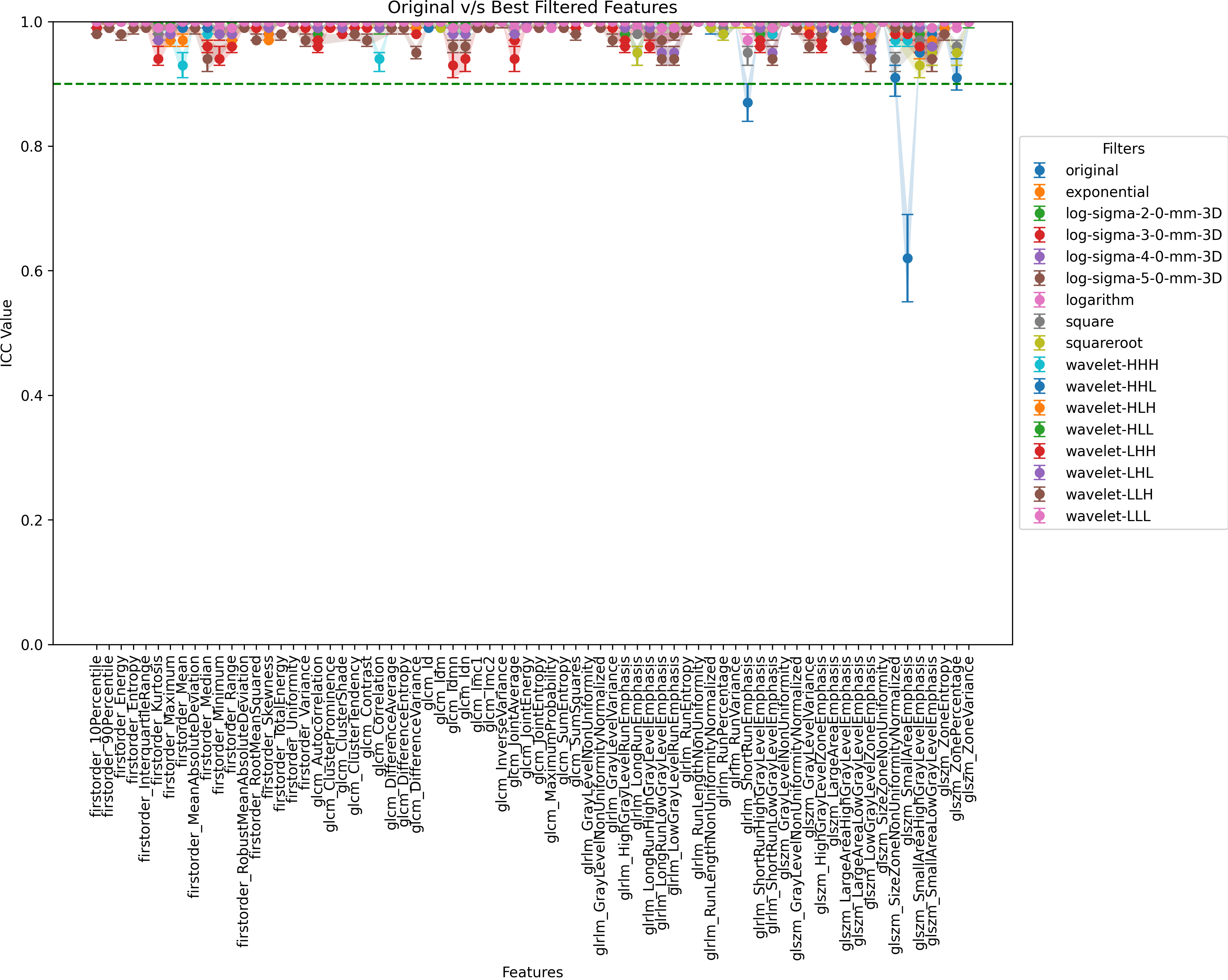

Supplement: Supplementary file 1 [file jpm-13-01172-s001.zip › plots/adc/out_plane_internal.png]

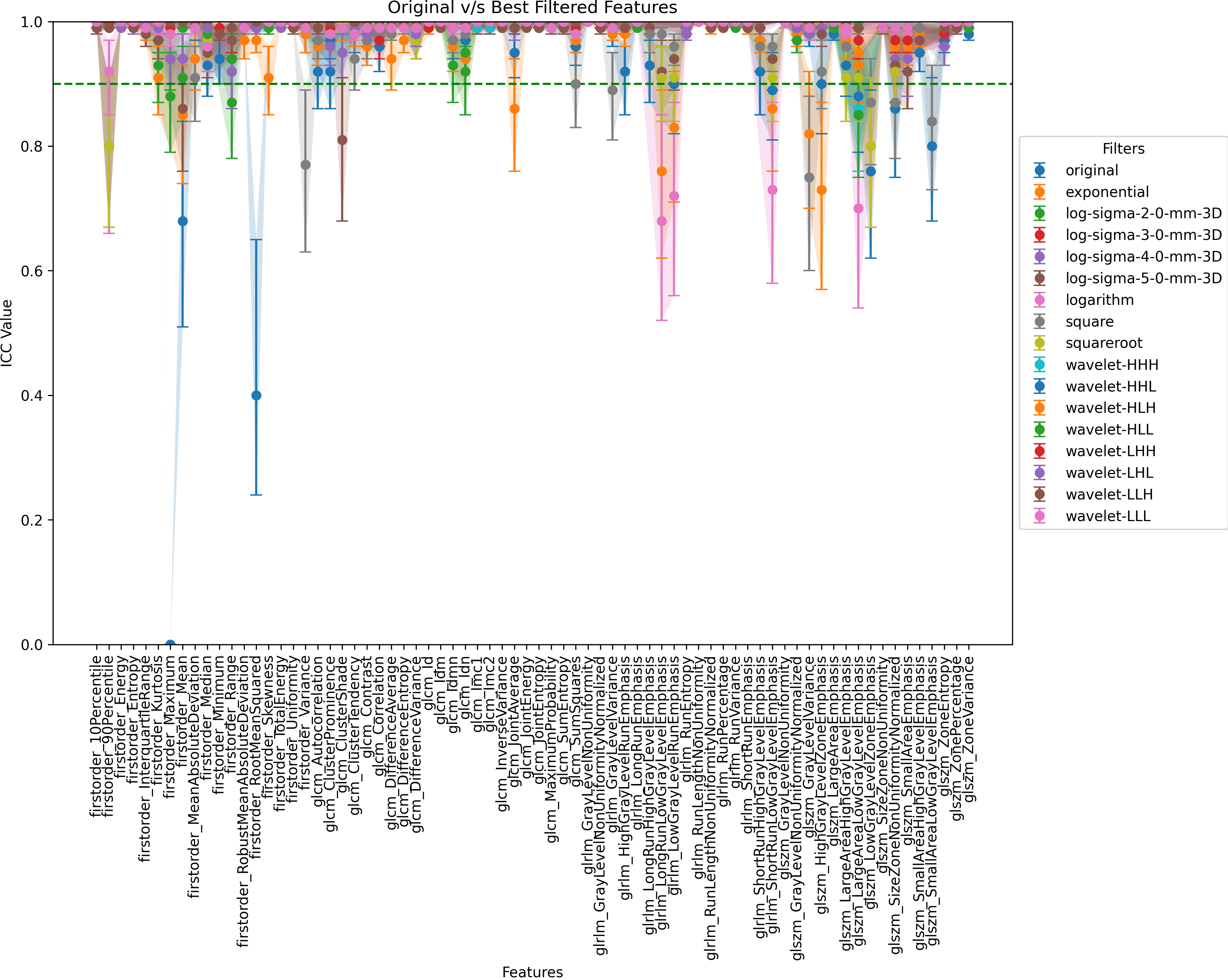

Supplement: Supplementary file 1 [file jpm-13-01172-s001.zip › plots/sub_win/in_plane_random_external.png]

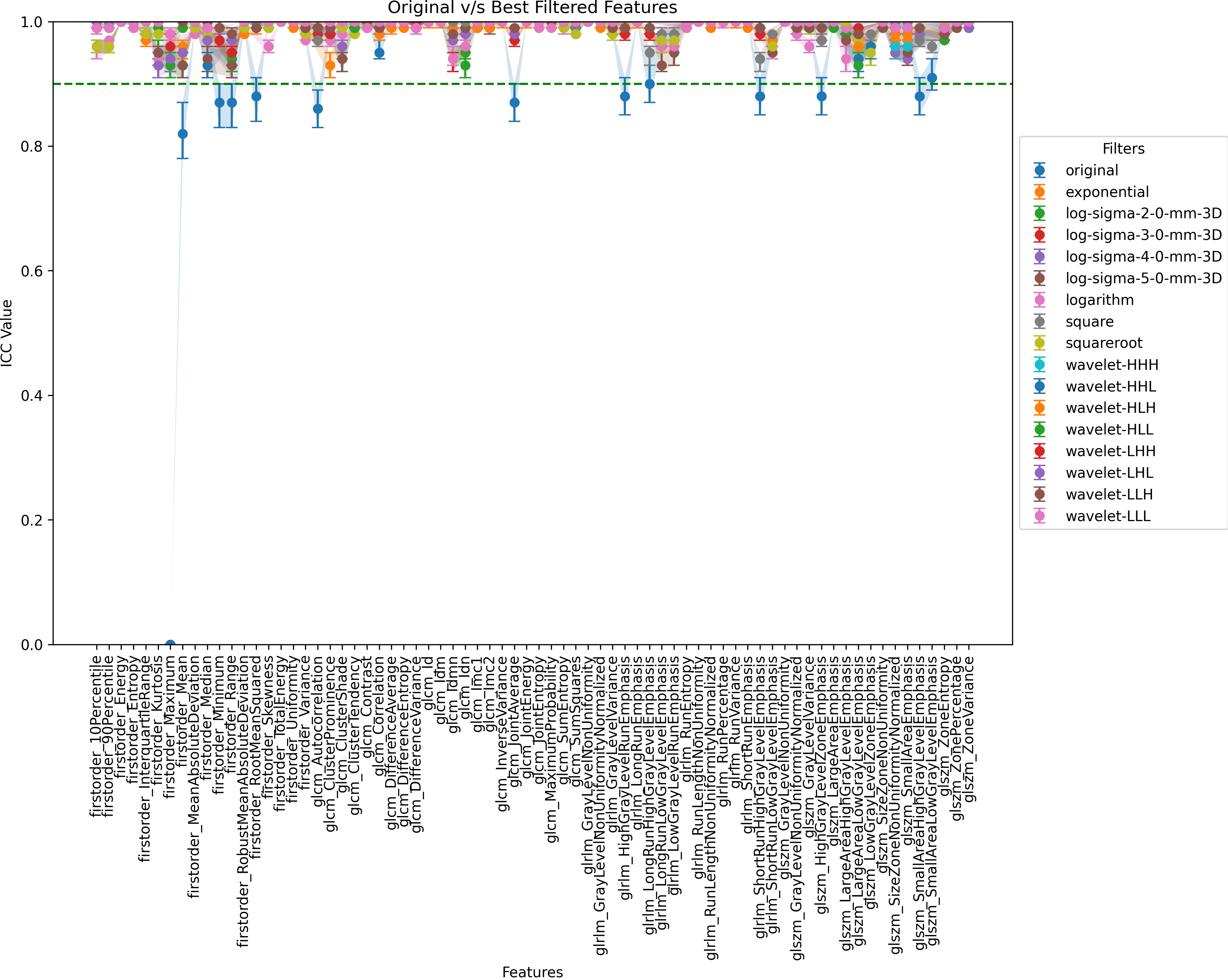

Supplement: Supplementary file 1 [file jpm-13-01172-s001.zip › plots/sub_win/in_plane_random_internal.png]

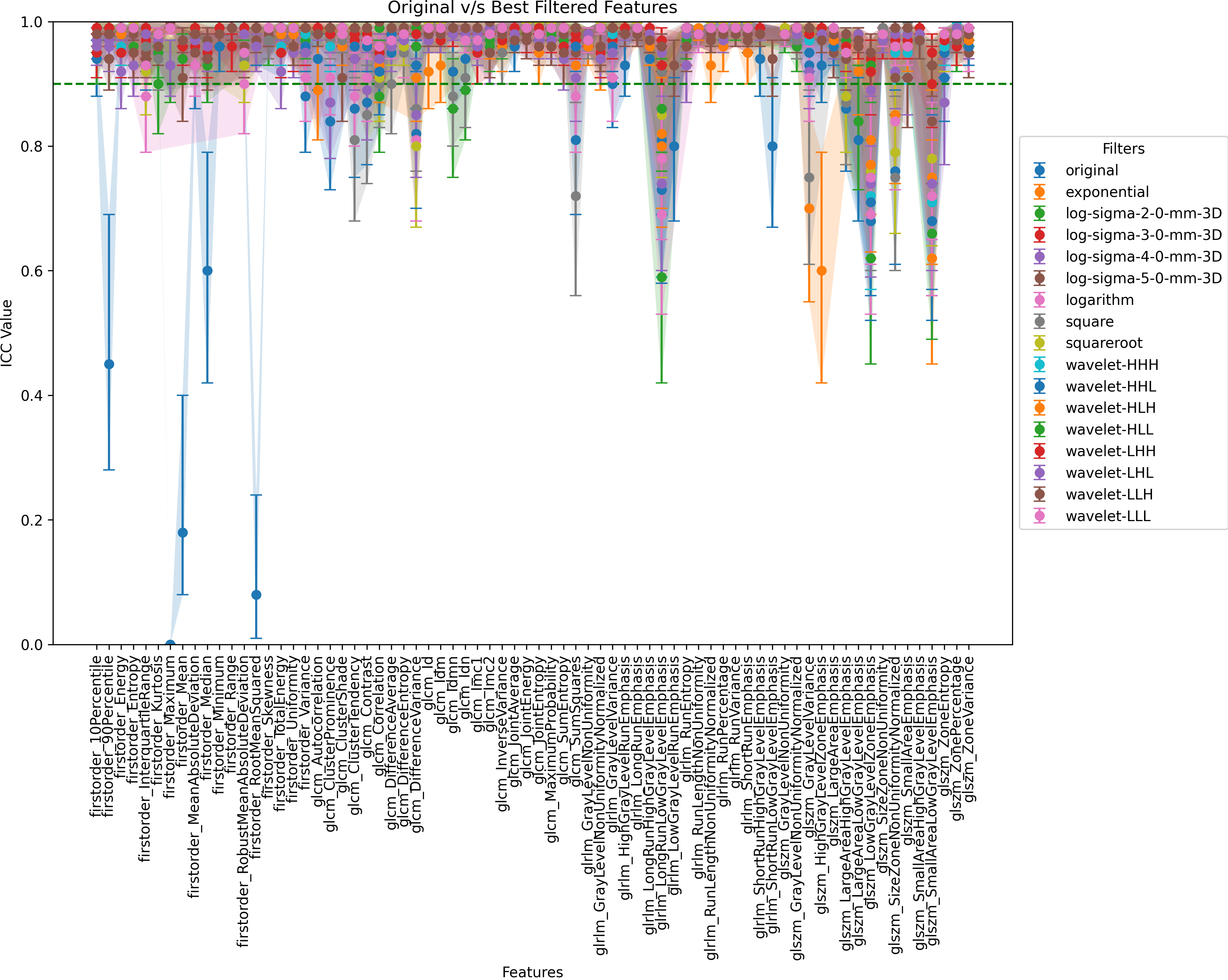

Supplement: Supplementary file 1 [file jpm-13-01172-s001.zip › plots/sub_win/in_plane_systematic_external.png]

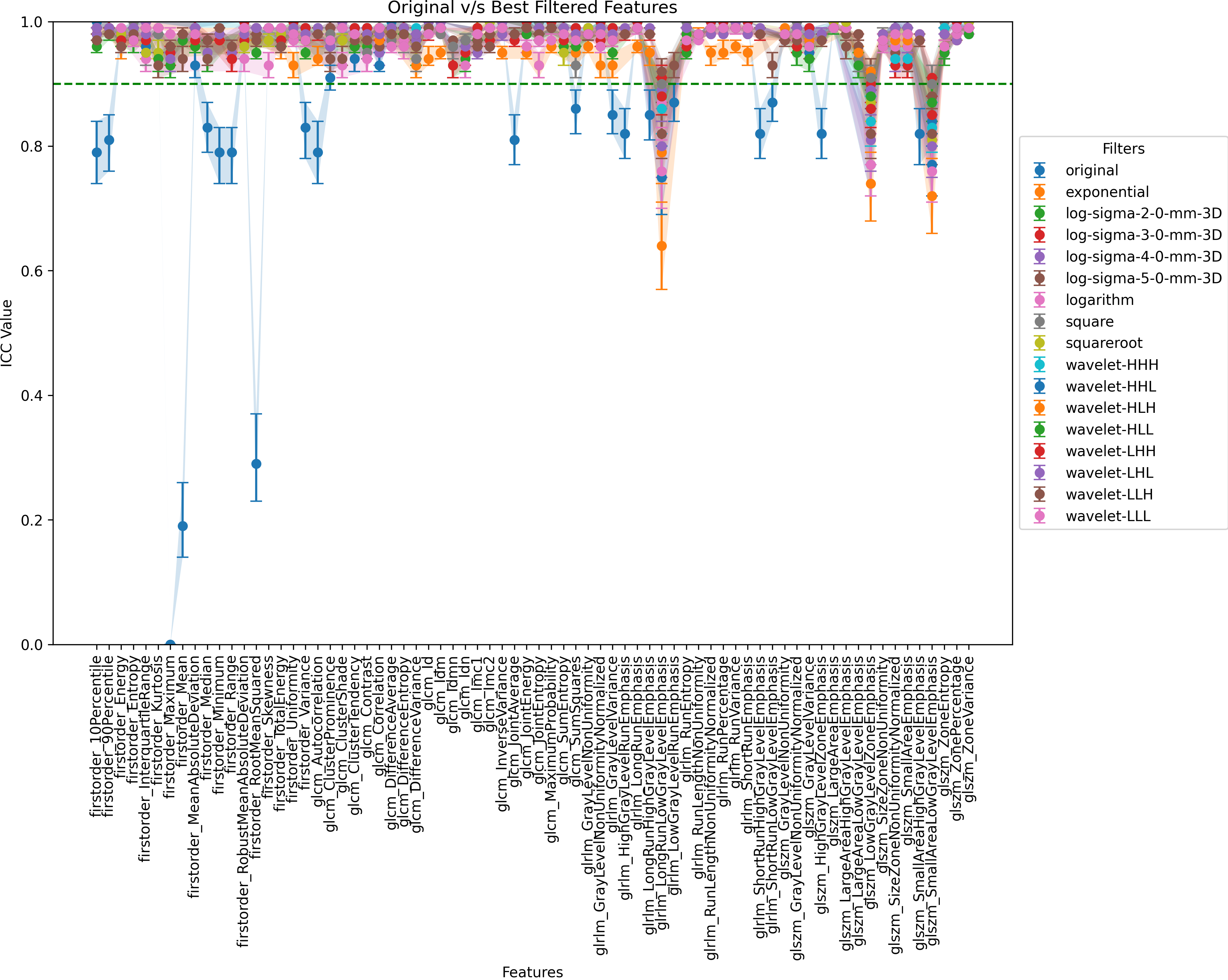

Supplement: Supplementary file 1 [file jpm-13-01172-s001.zip › plots/sub_win/in_plane_systematic_internal.png]

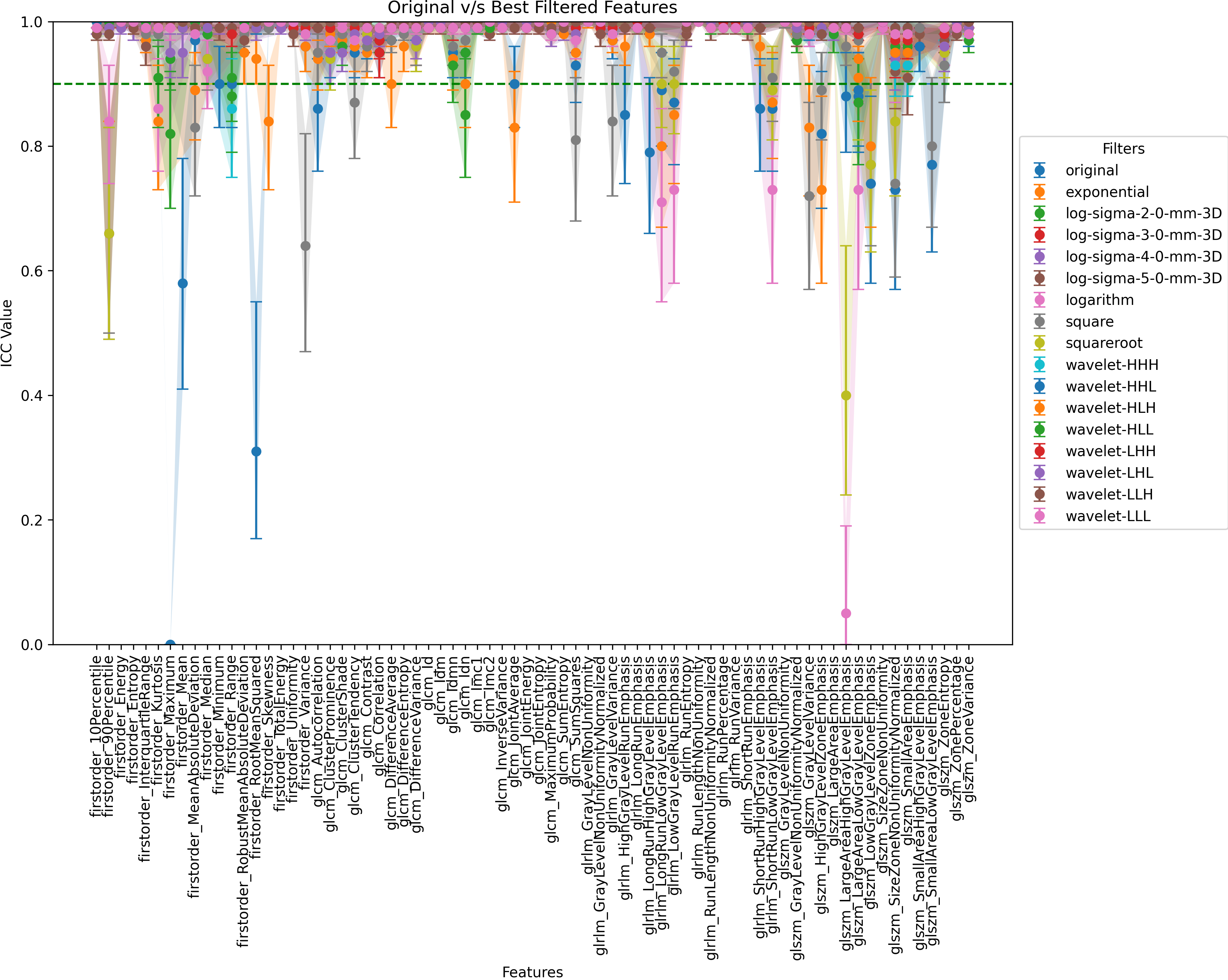

Supplement: Supplementary file 1 [file jpm-13-01172-s001.zip › plots/sub_win/inout_plane_random_external.png]

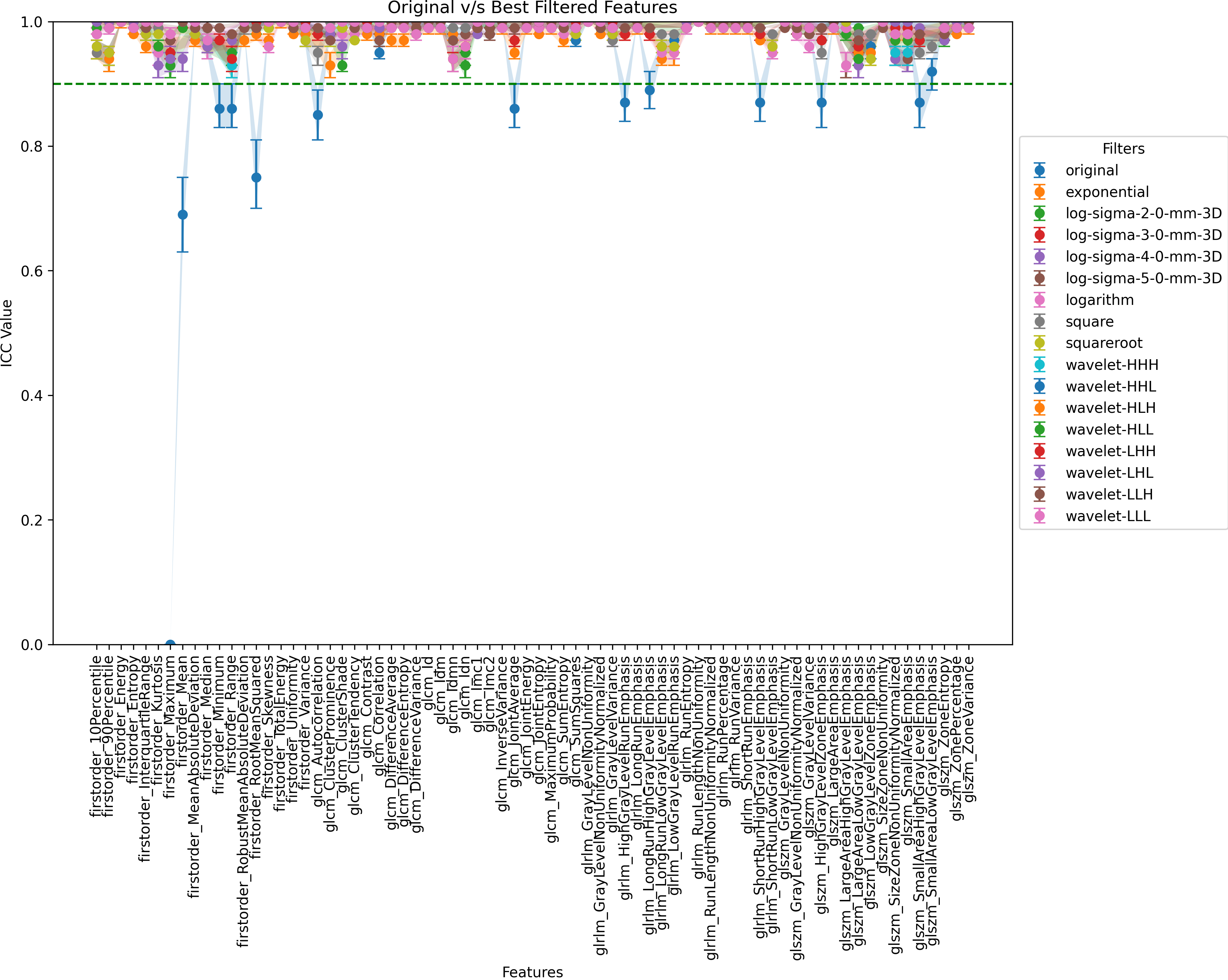

Supplement: Supplementary file 1 [file jpm-13-01172-s001.zip › plots/sub_win/inout_plane_random_internal.png]

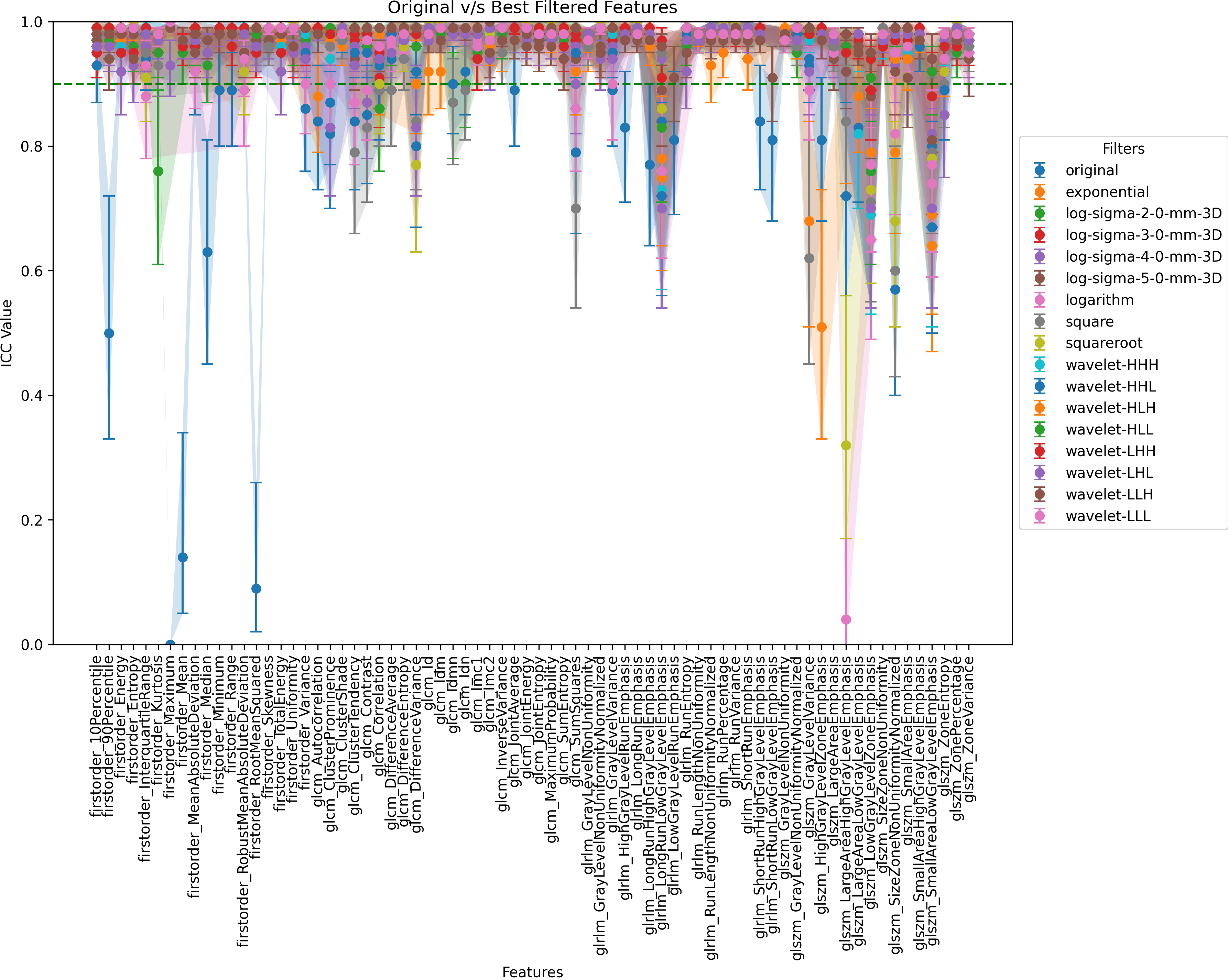

Supplement: Supplementary file 1 [file jpm-13-01172-s001.zip › plots/sub_win/inout_plane_systematic_external.png]

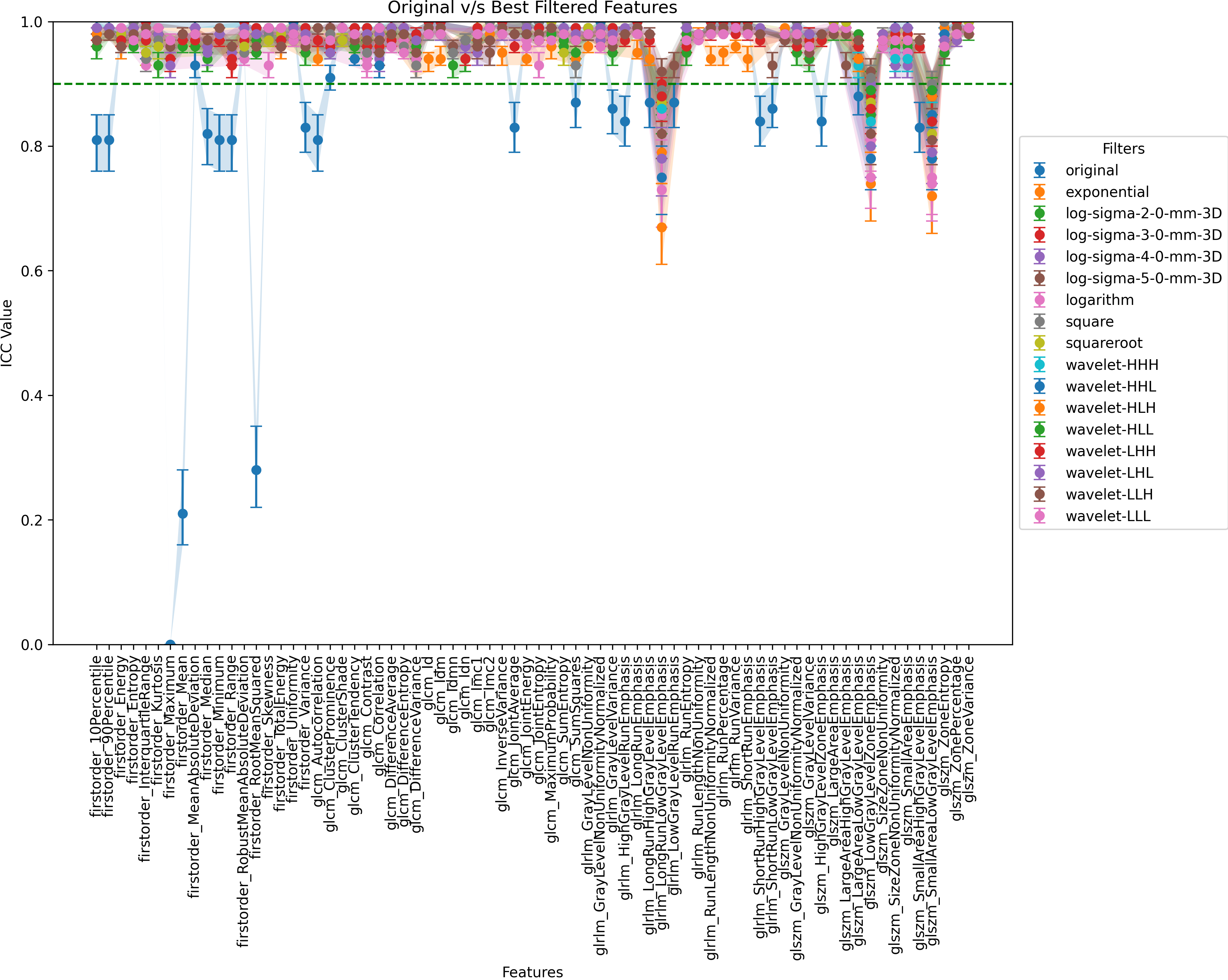

Supplement: Supplementary file 1 [file jpm-13-01172-s001.zip › plots/sub_win/inout_plane_systematic_internal.png]

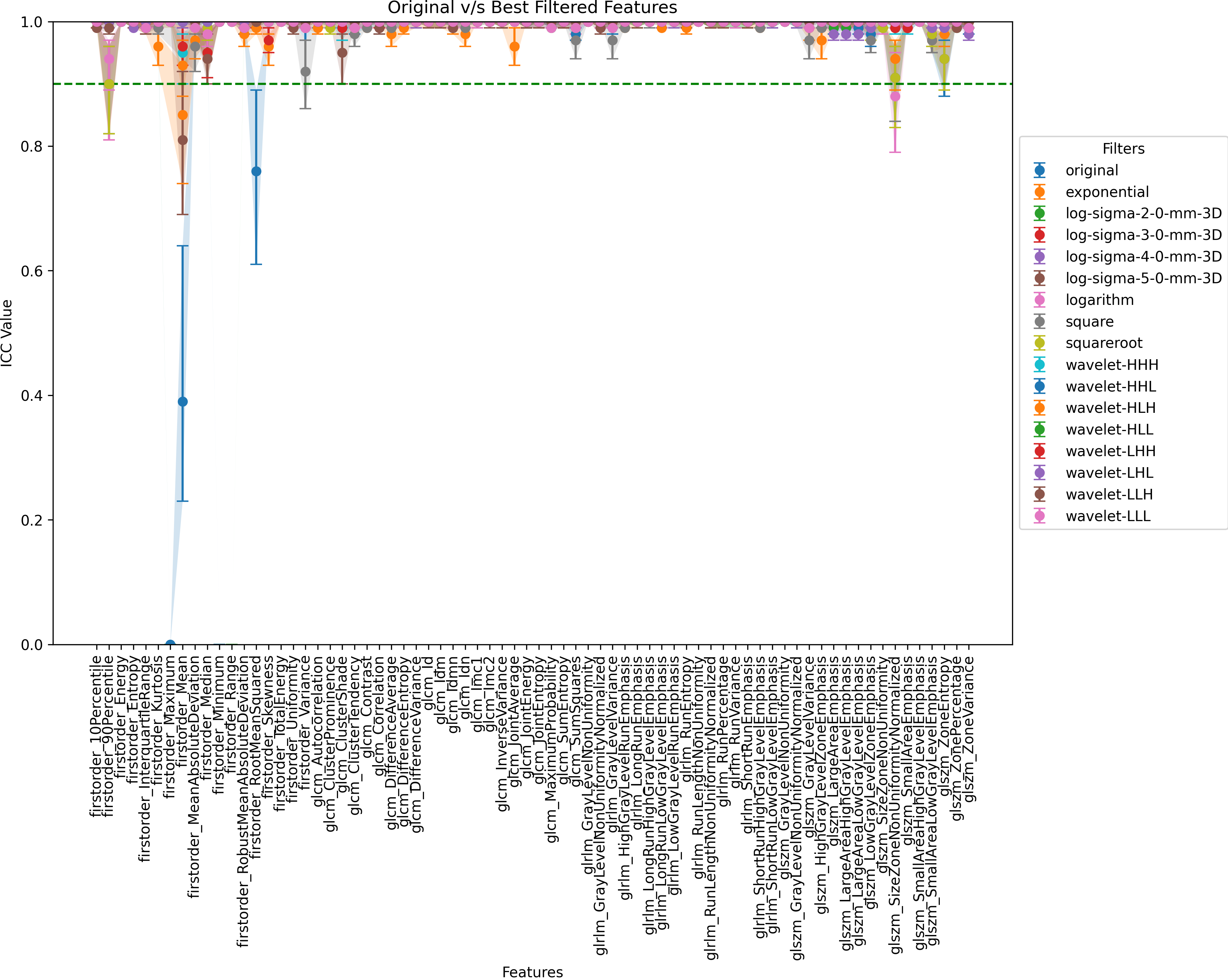

Supplement: Supplementary file 1 [file jpm-13-01172-s001.zip › plots/sub_win/out_plane_external.png]

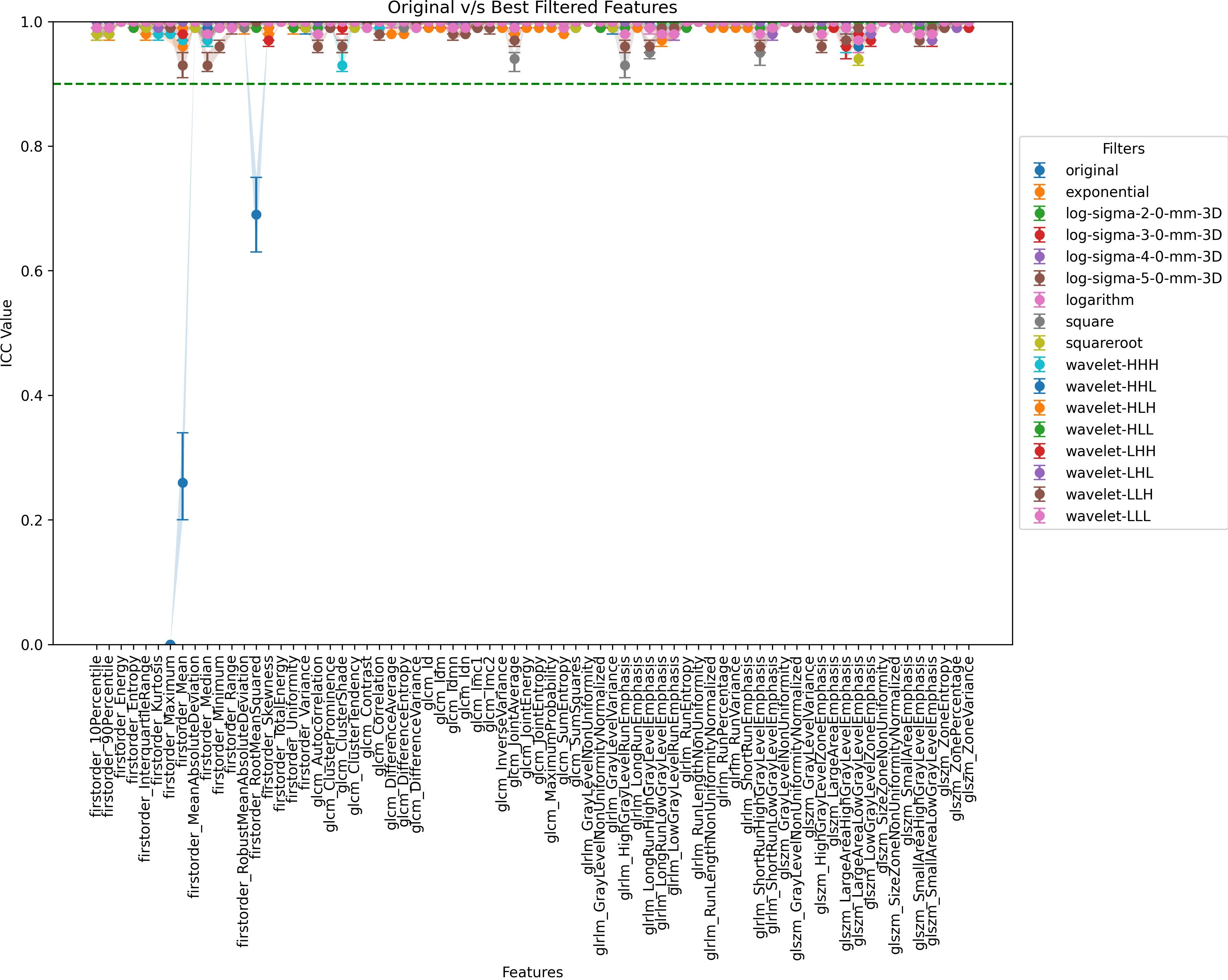

Supplement: Supplementary file 1 [file jpm-13-01172-s001.zip › plots/sub_win/out_plane_internal.png]

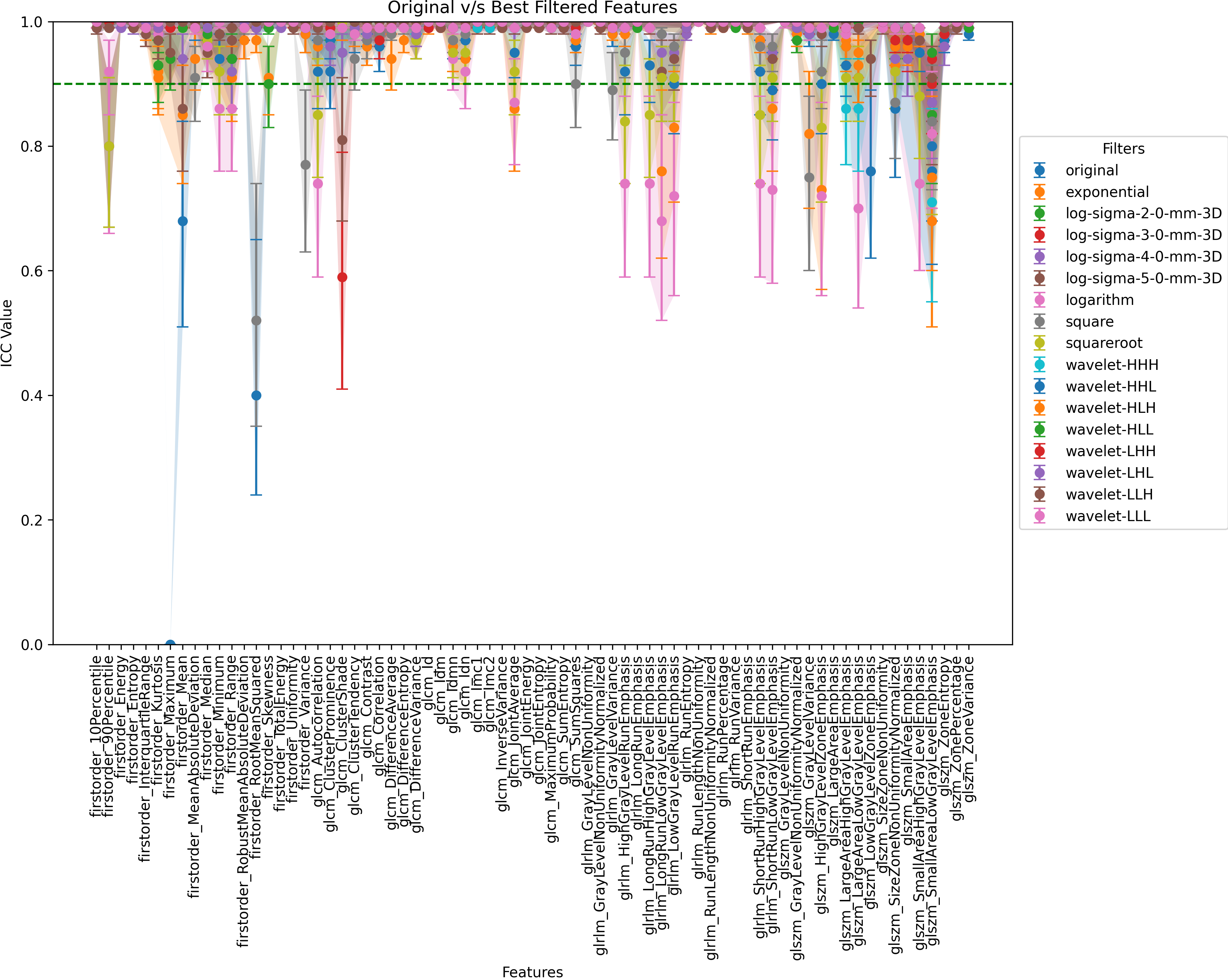

Supplement: Supplementary file 1 [file jpm-13-01172-s001.zip › plots/sub_wout/in_plane_random_external.png]

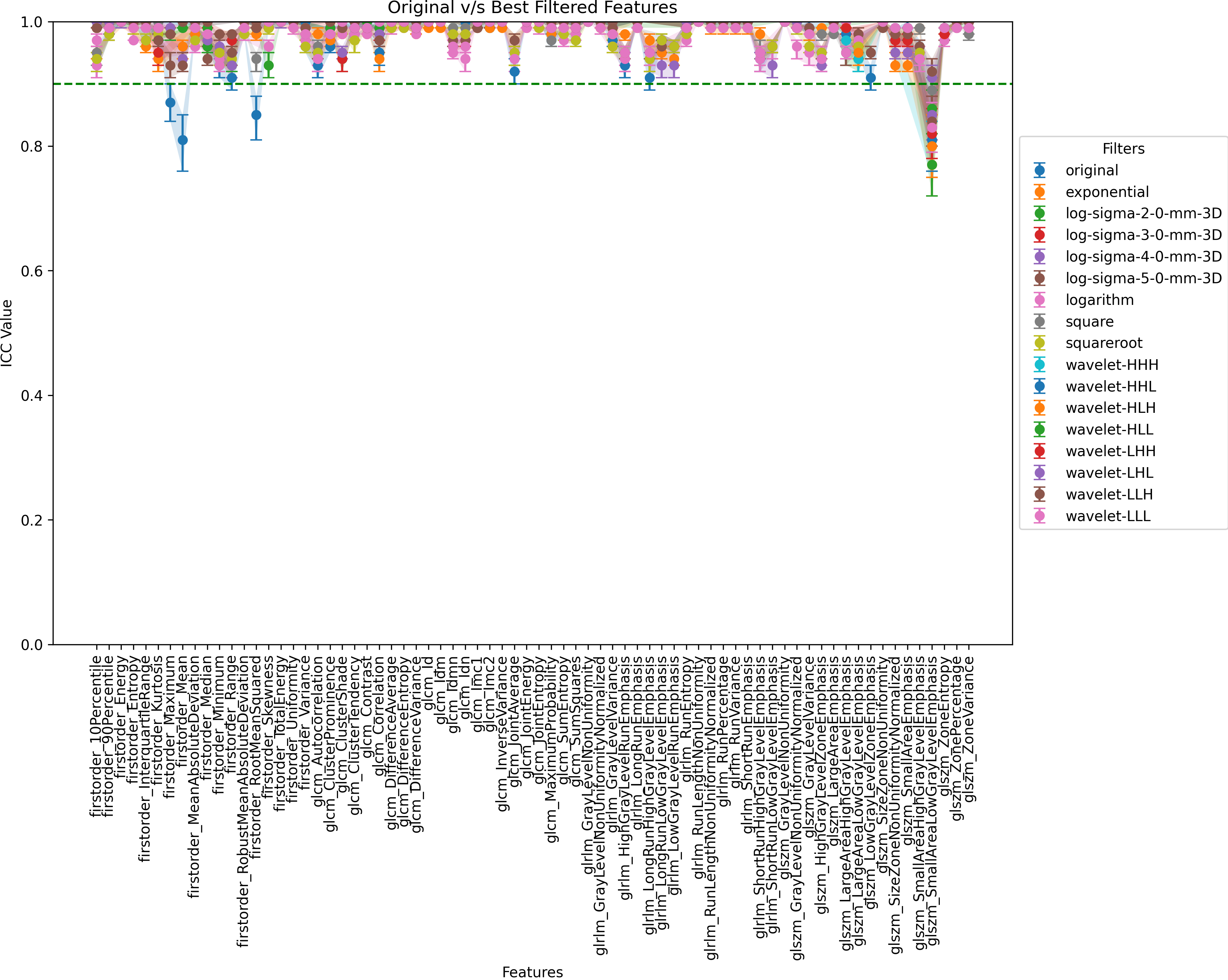

Supplement: Supplementary file 1 [file jpm-13-01172-s001.zip › plots/sub_wout/in_plane_random_internal.png]

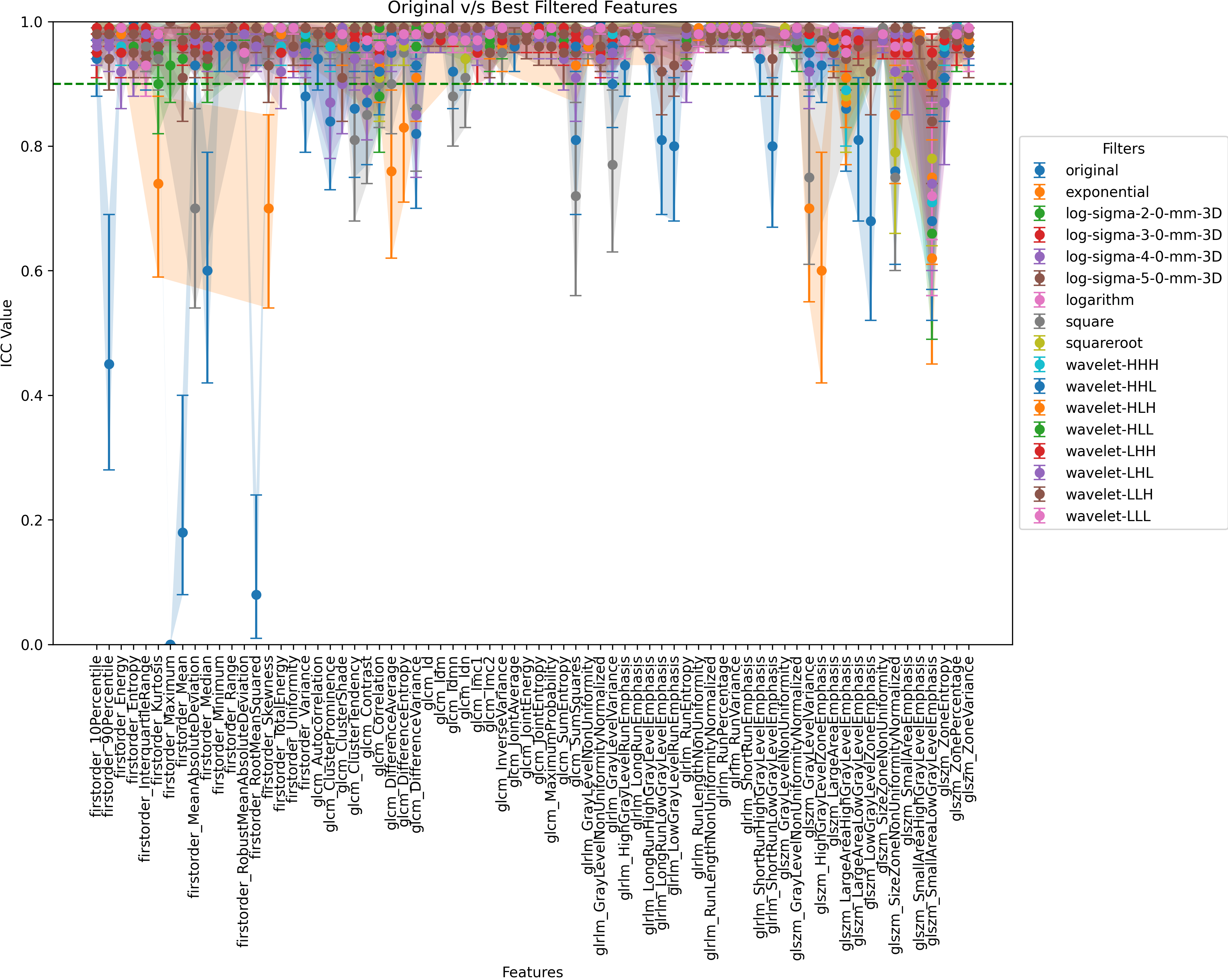

Supplement: Supplementary file 1 [file jpm-13-01172-s001.zip › plots/sub_wout/in_plane_systematic_external.png]

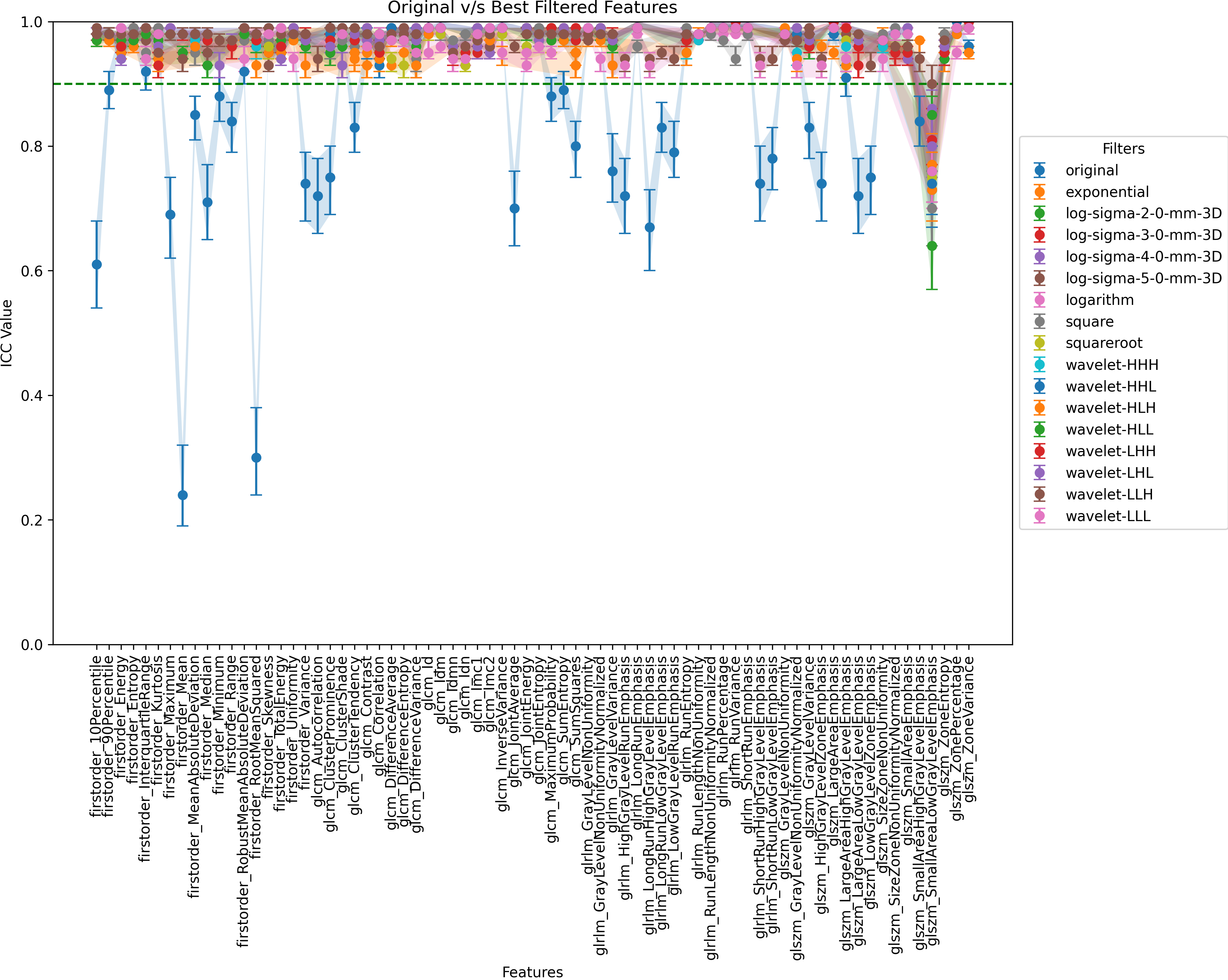

Supplement: Supplementary file 1 [file jpm-13-01172-s001.zip › plots/sub_wout/in_plane_systematic_internal.png]

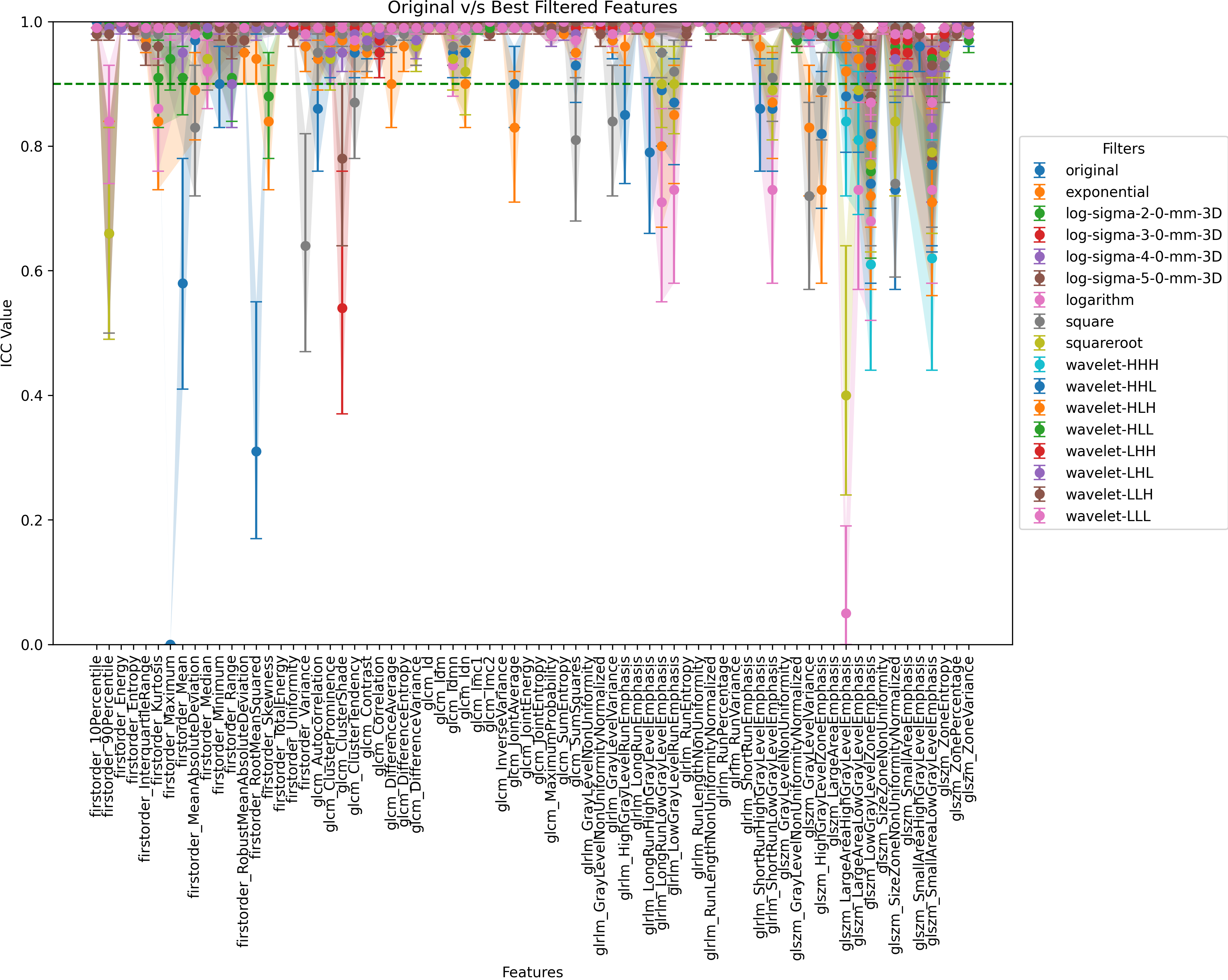

Supplement: Supplementary file 1 [file jpm-13-01172-s001.zip › plots/sub_wout/inout_plane_random_external.png]

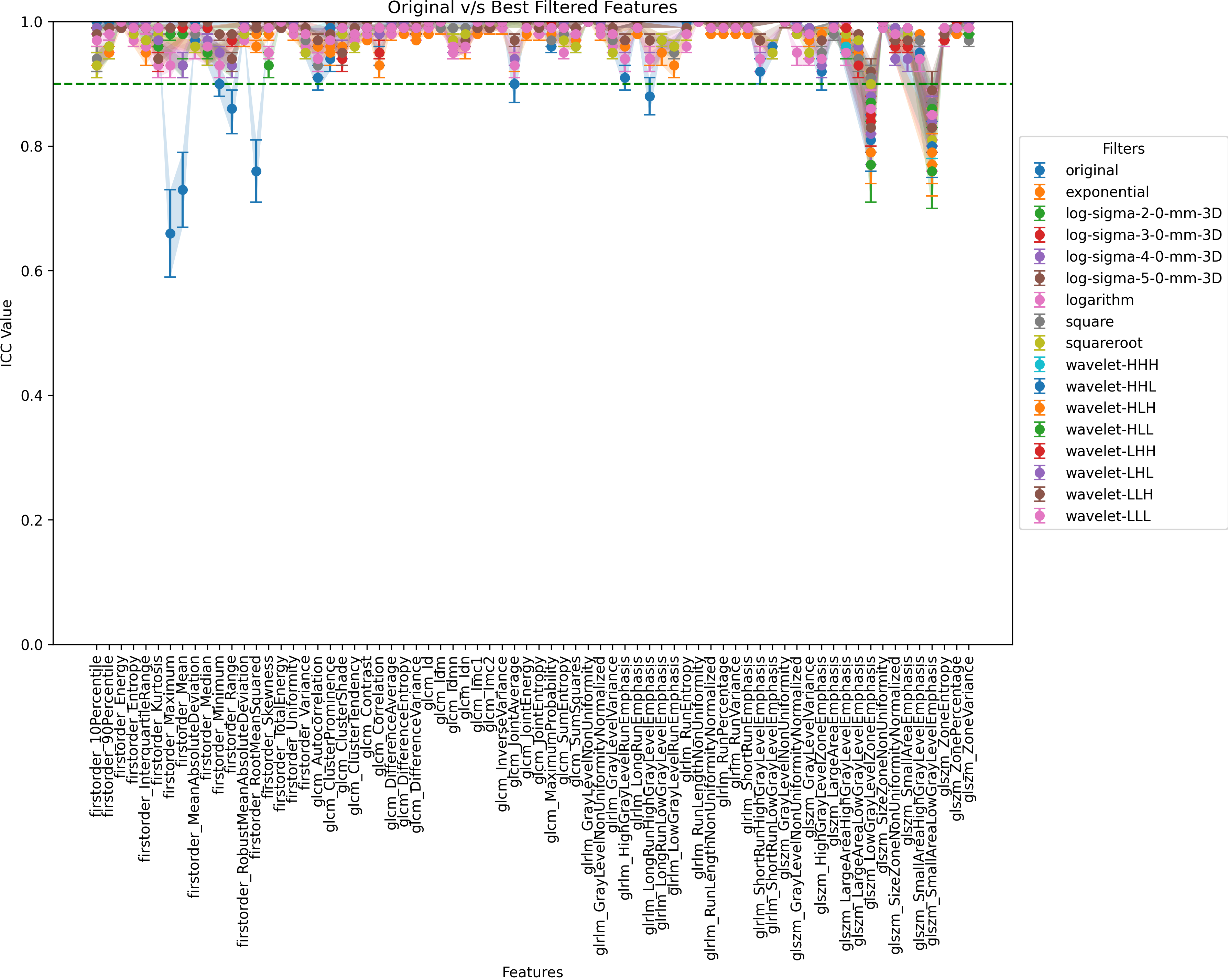

Supplement: Supplementary file 1 [file jpm-13-01172-s001.zip › plots/sub_wout/inout_plane_random_internal.png]

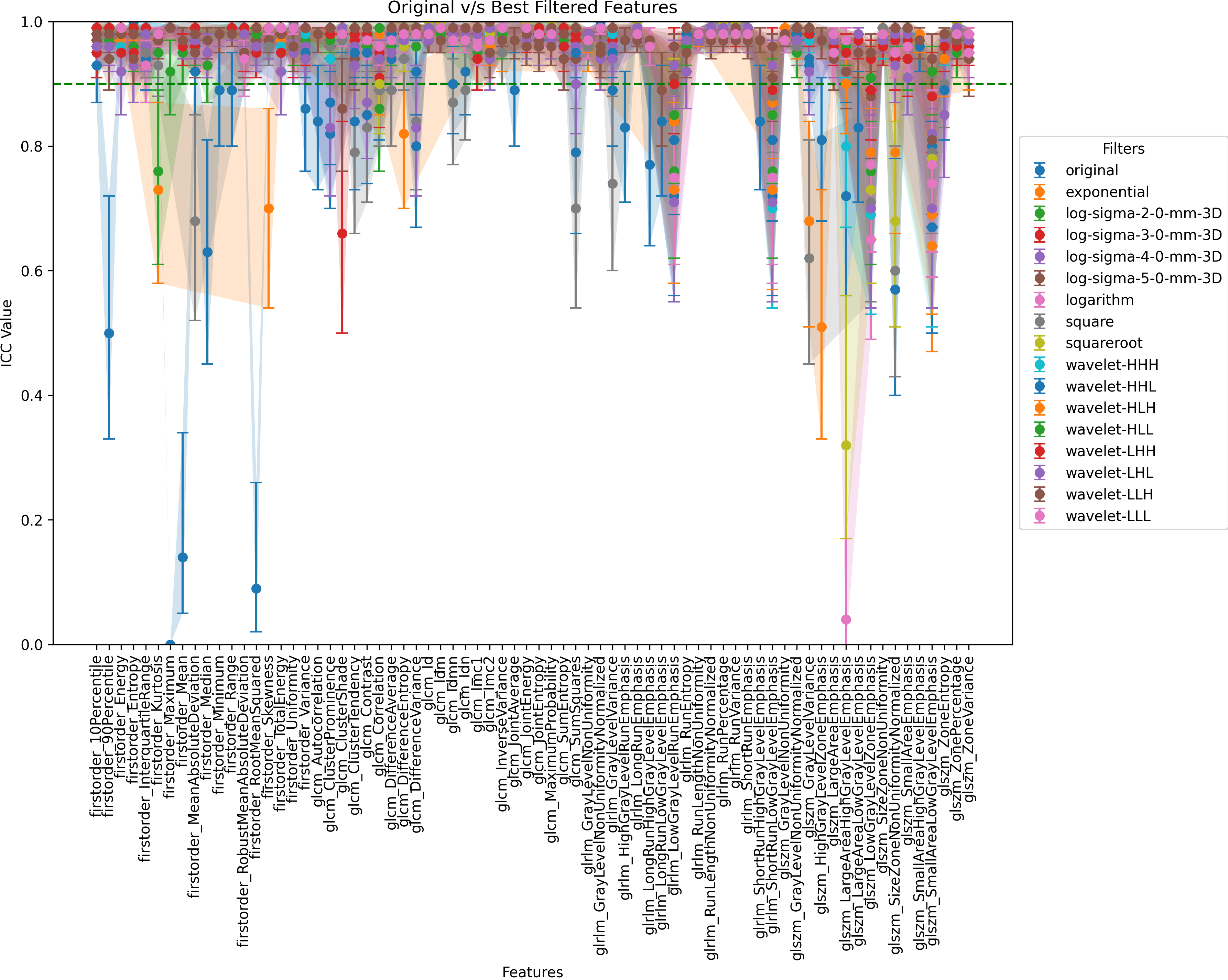

Supplement: Supplementary file 1 [file jpm-13-01172-s001.zip › plots/sub_wout/inout_plane_systematic_external.png]

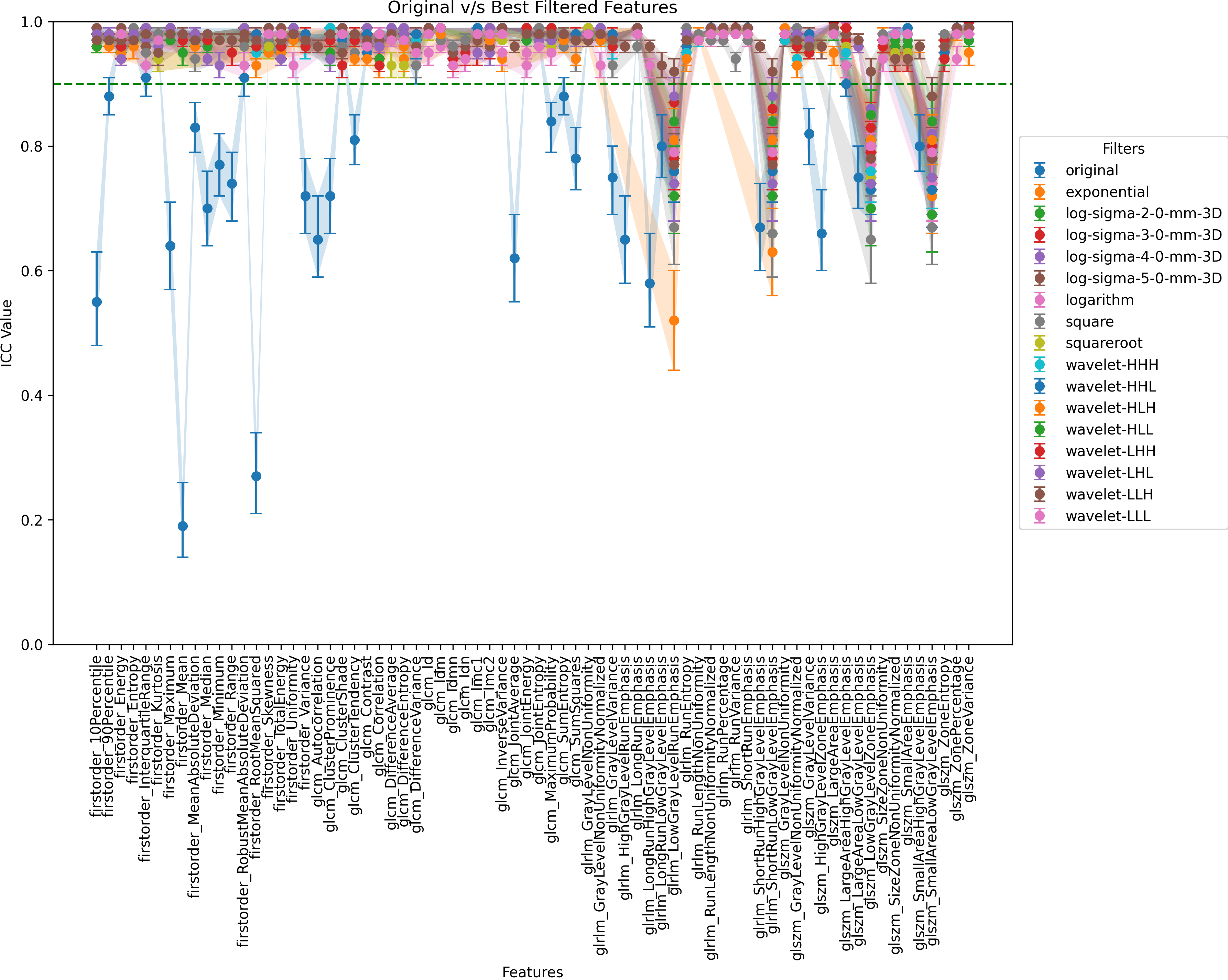

Supplement: Supplementary file 1 [file jpm-13-01172-s001.zip › plots/sub_wout/inout_plane_systematic_internal.png]

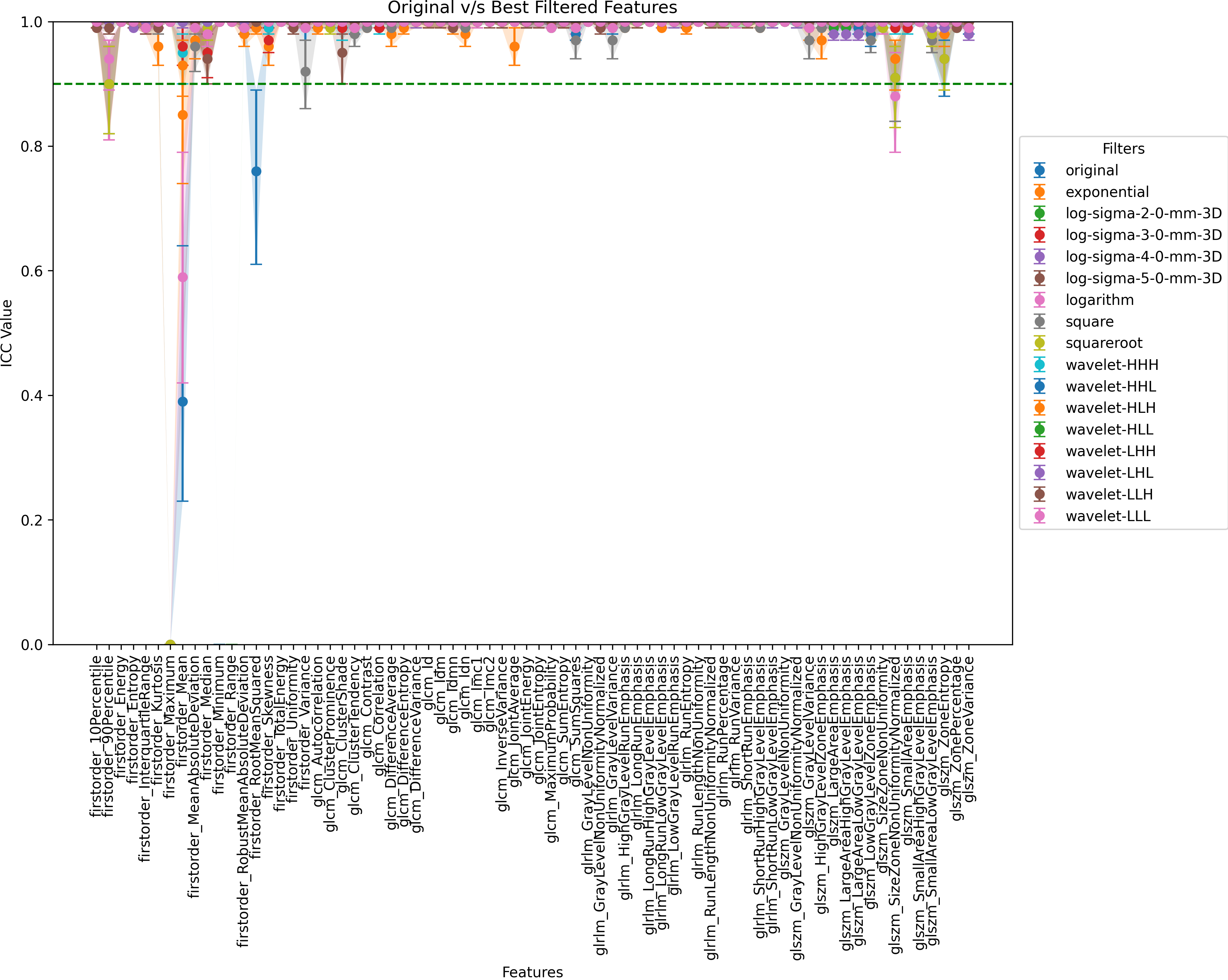

Supplement: Supplementary file 1 [file jpm-13-01172-s001.zip › plots/sub_wout/out_plane_external.png]

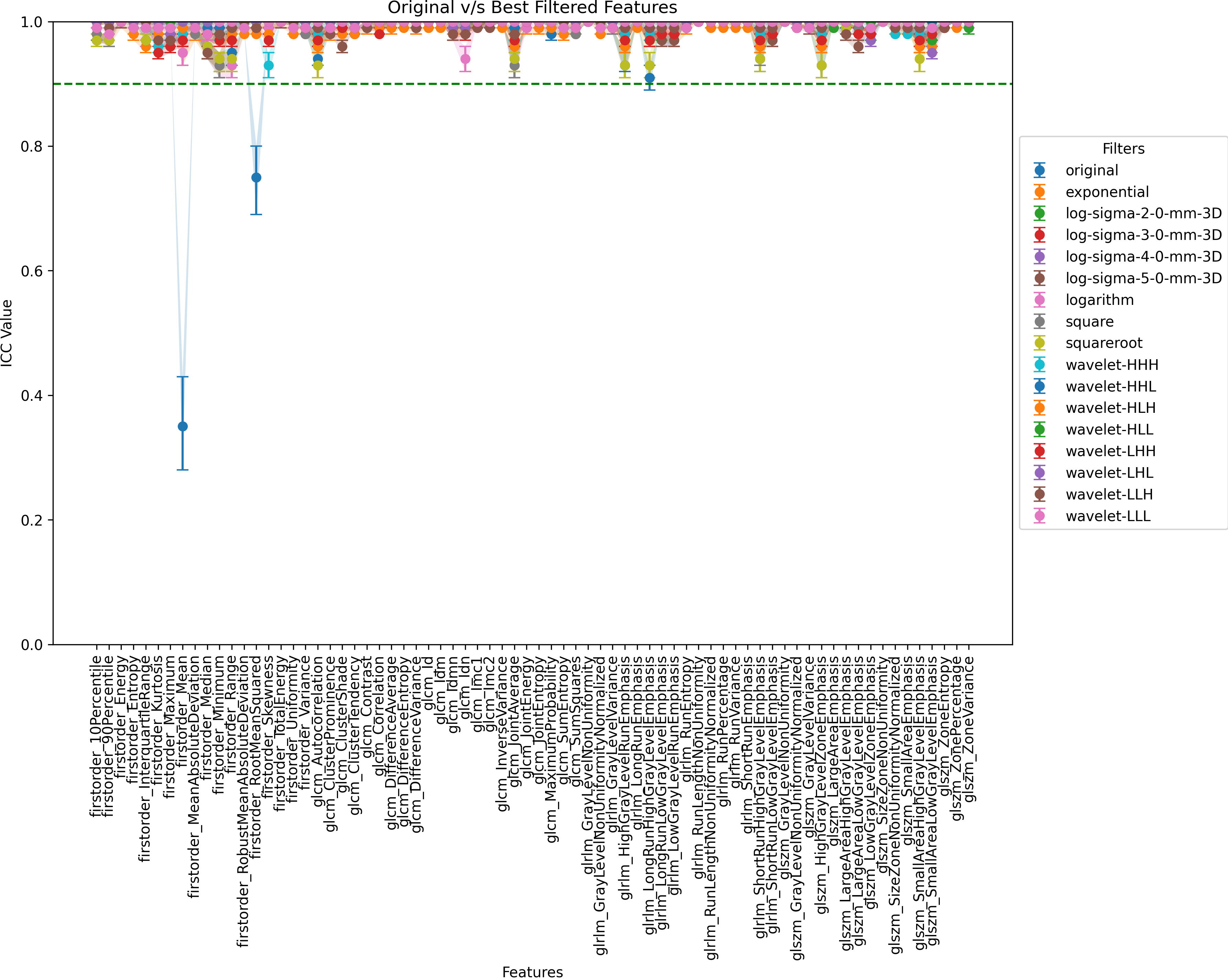

Supplement: Supplementary file 1 [file jpm-13-01172-s001.zip › plots/sub_wout/out_plane_internal.png]

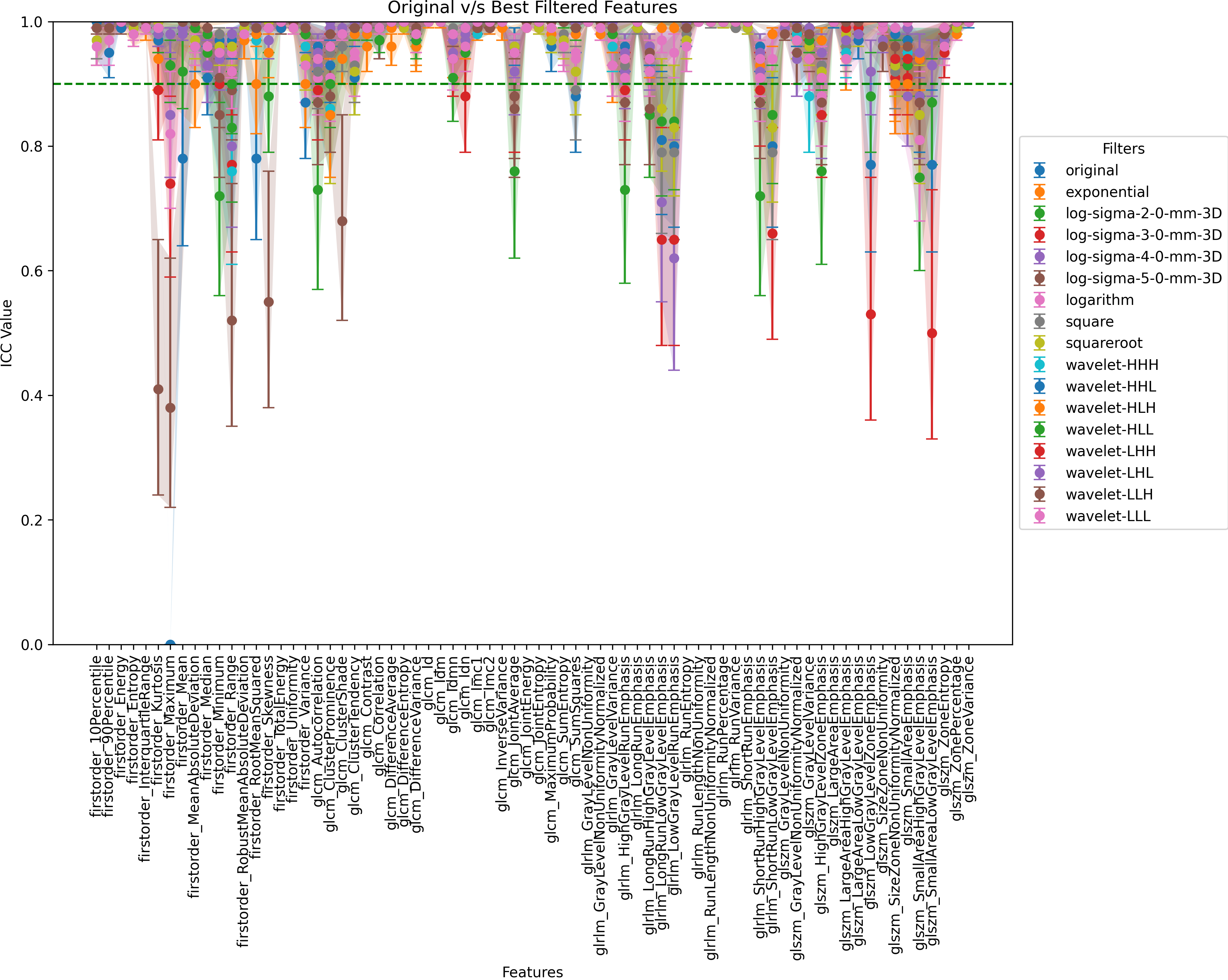

Supplement: Supplementary file 1 [file jpm-13-01172-s001.zip › plots/t2w/in_plane_random_external.png]

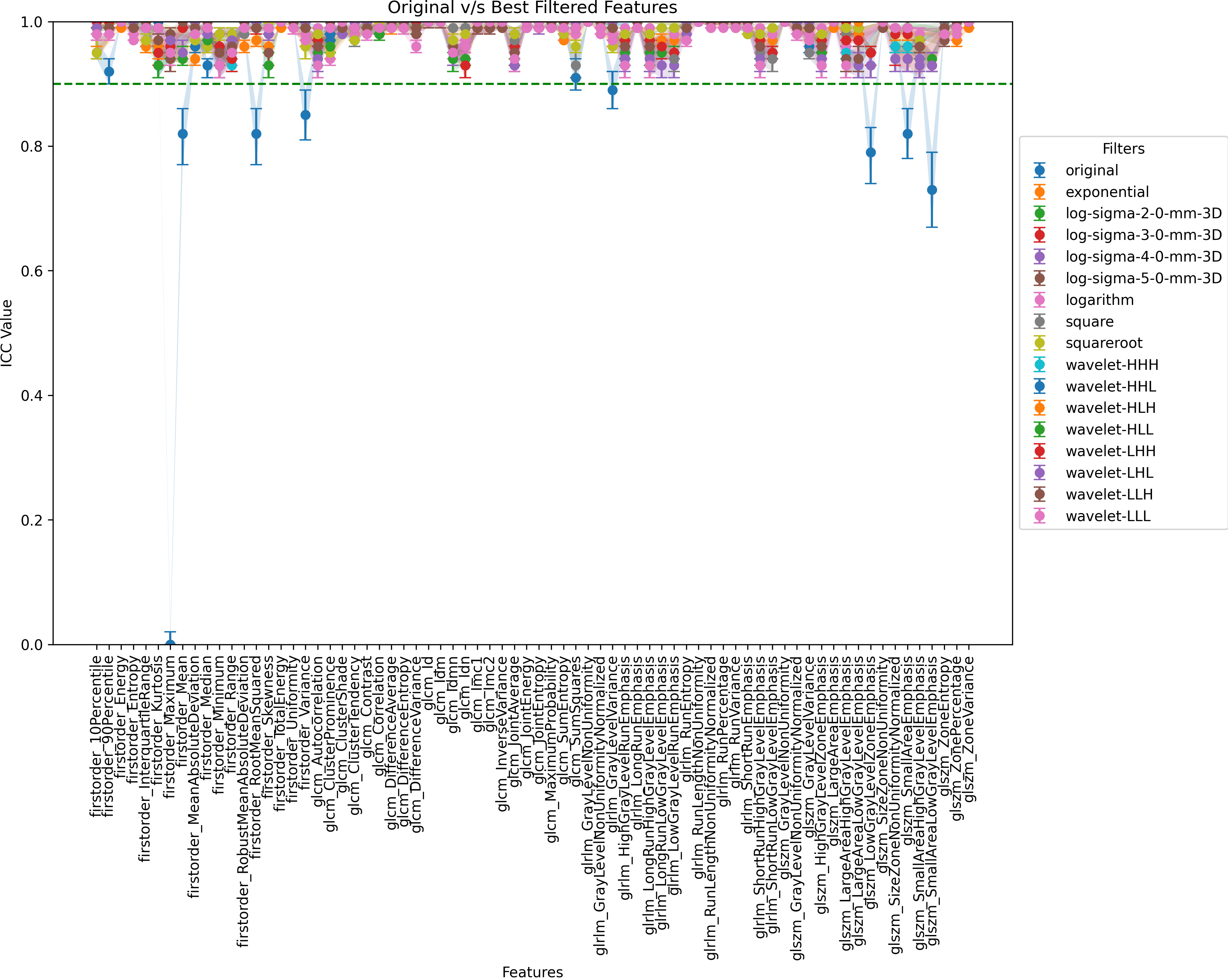

Supplement: Supplementary file 1 [file jpm-13-01172-s001.zip › plots/t2w/in_plane_random_internal.png]

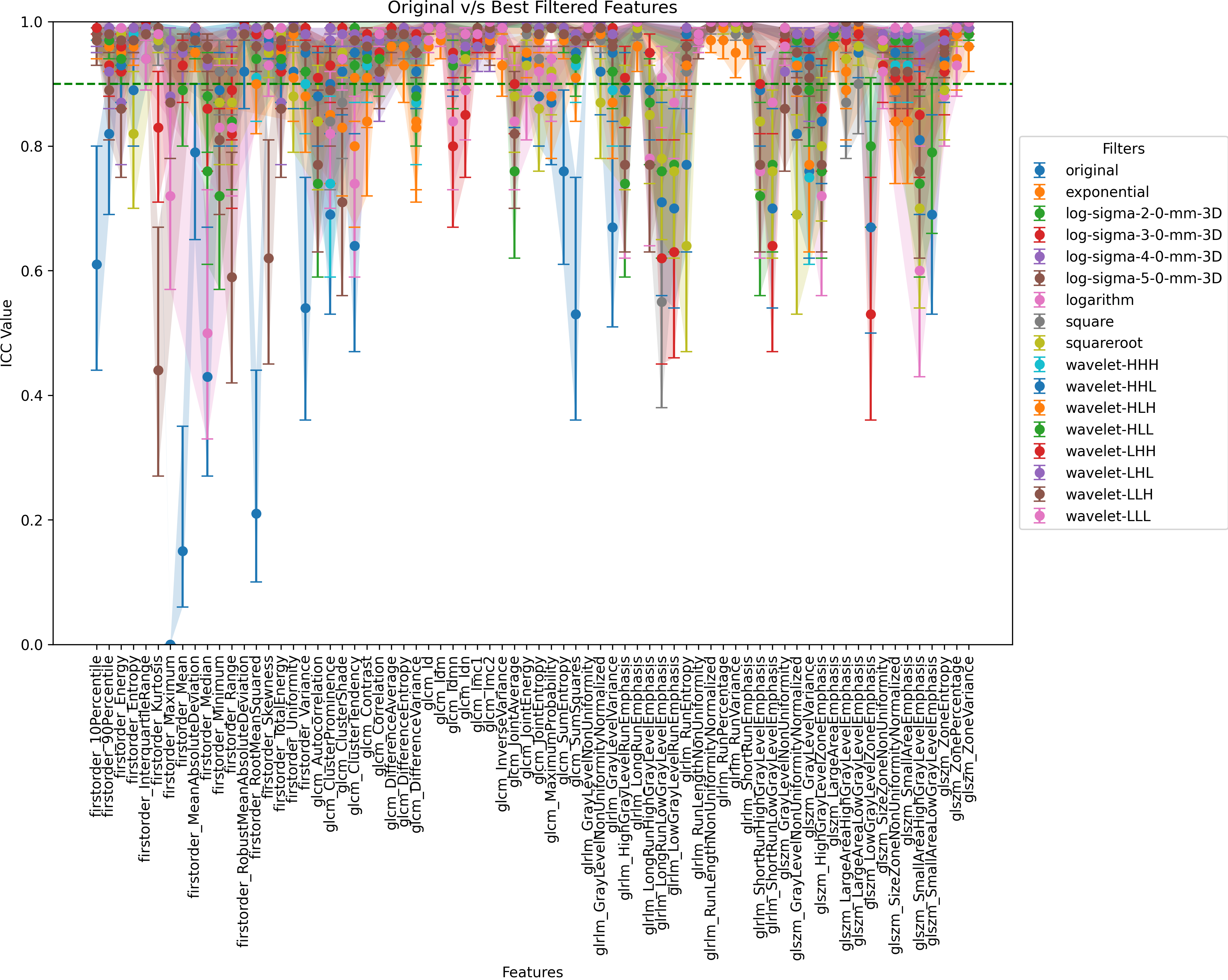

Supplement: Supplementary file 1 [file jpm-13-01172-s001.zip › plots/t2w/in_plane_systematic_external.png]

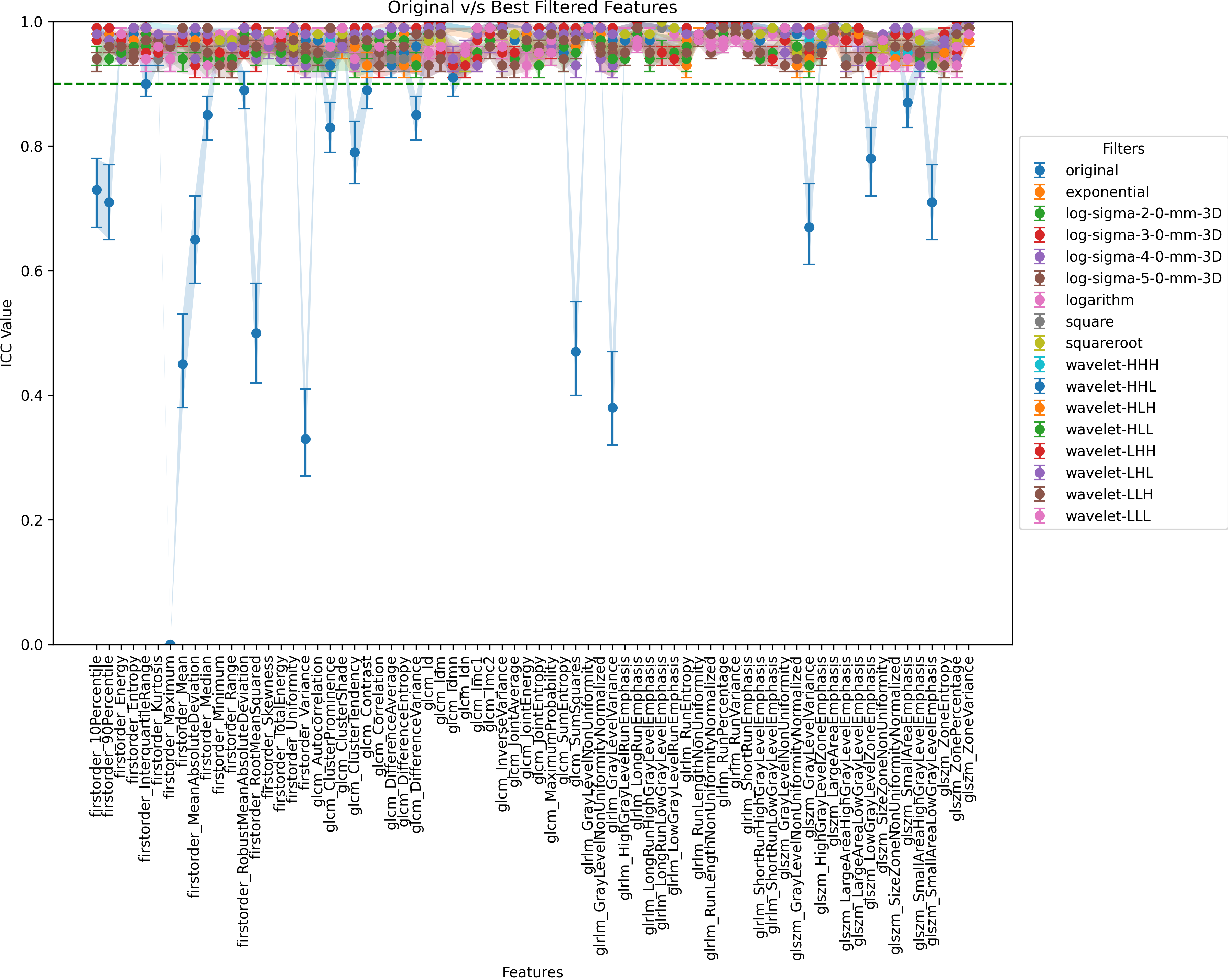

Supplement: Supplementary file 1 [file jpm-13-01172-s001.zip › plots/t2w/in_plane_systematic_internal.png]

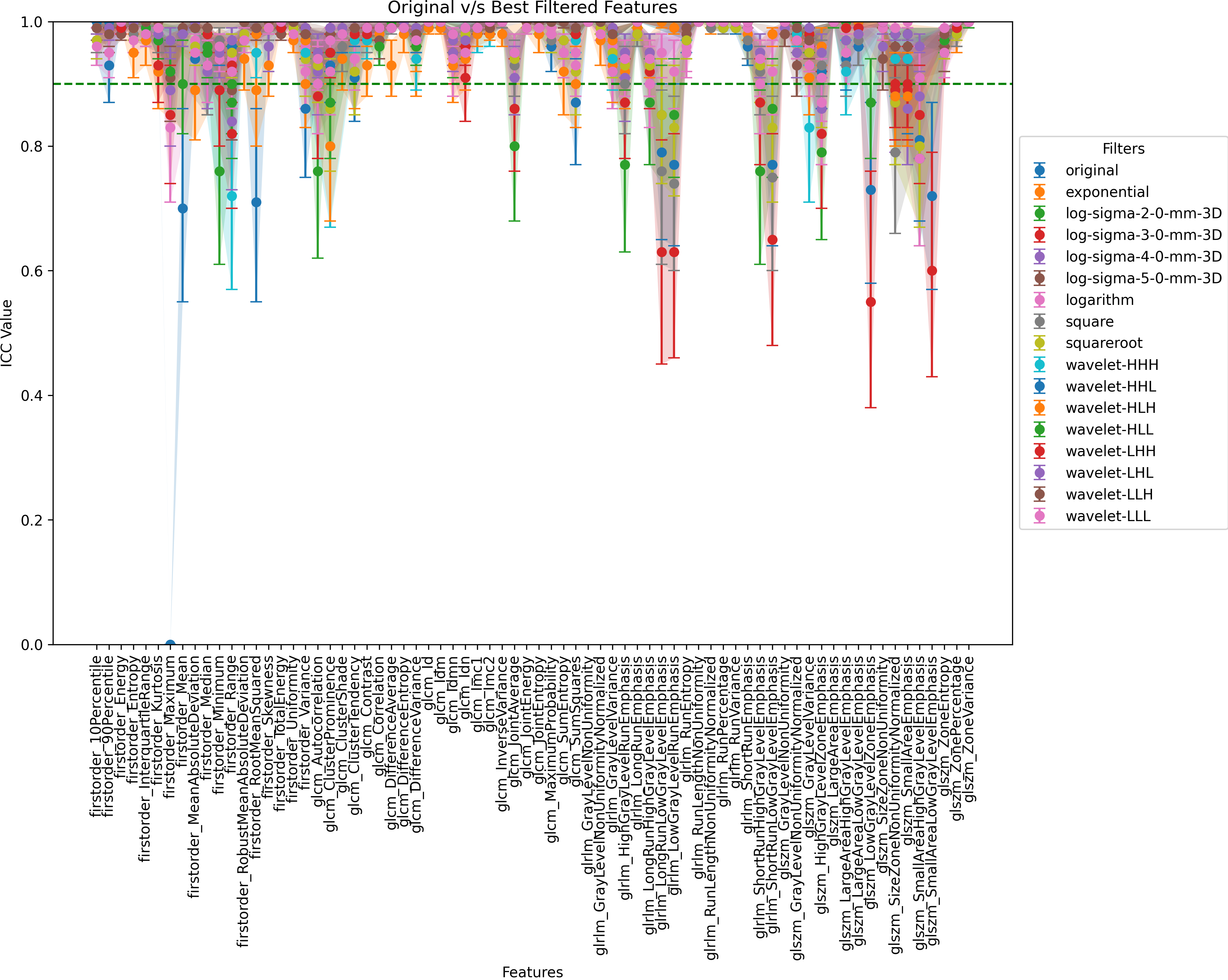

Supplement: Supplementary file 1 [file jpm-13-01172-s001.zip › plots/t2w/inout_plane_random_external.png]

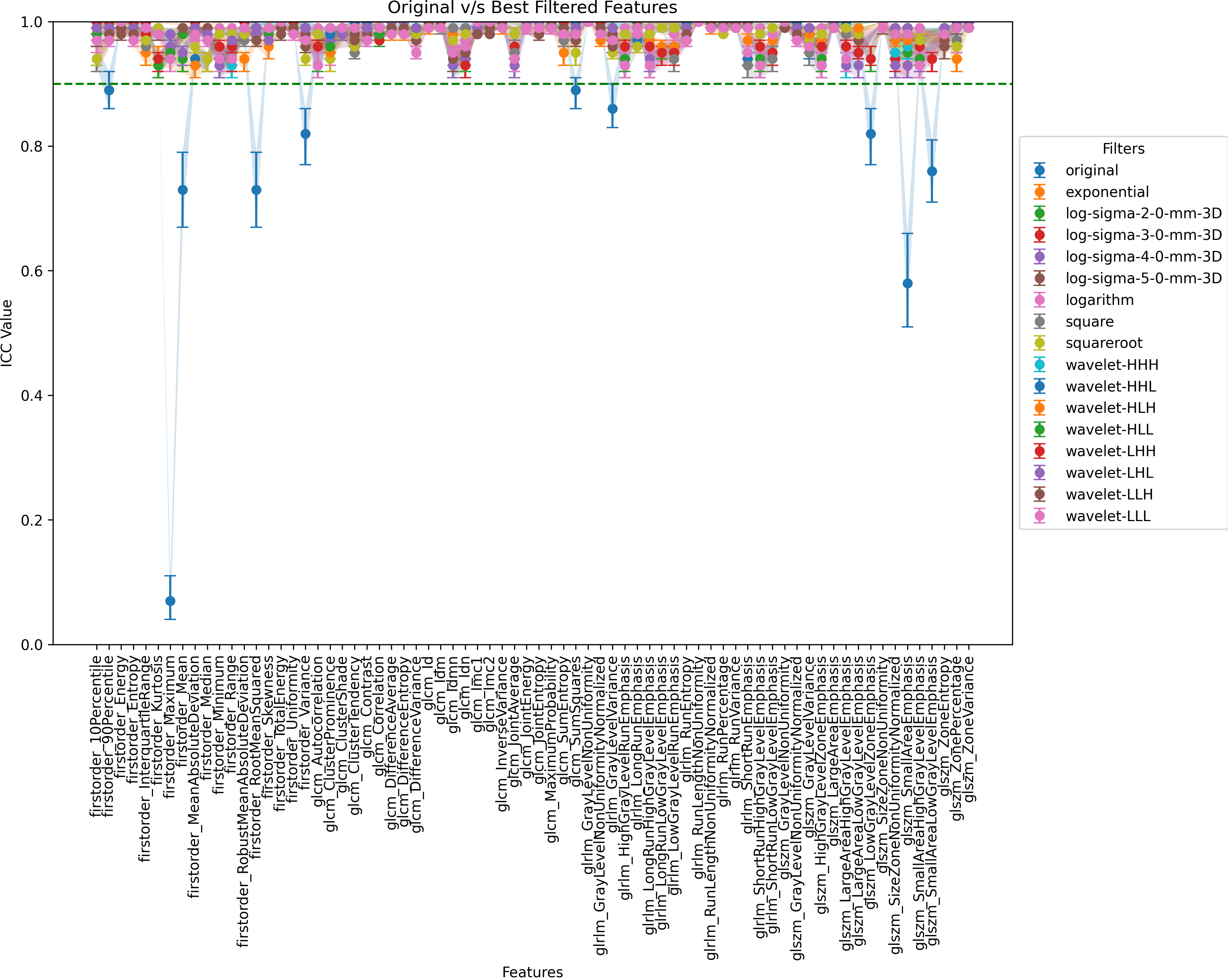

Supplement: Supplementary file 1 [file jpm-13-01172-s001.zip › plots/t2w/inout_plane_random_internal.png]

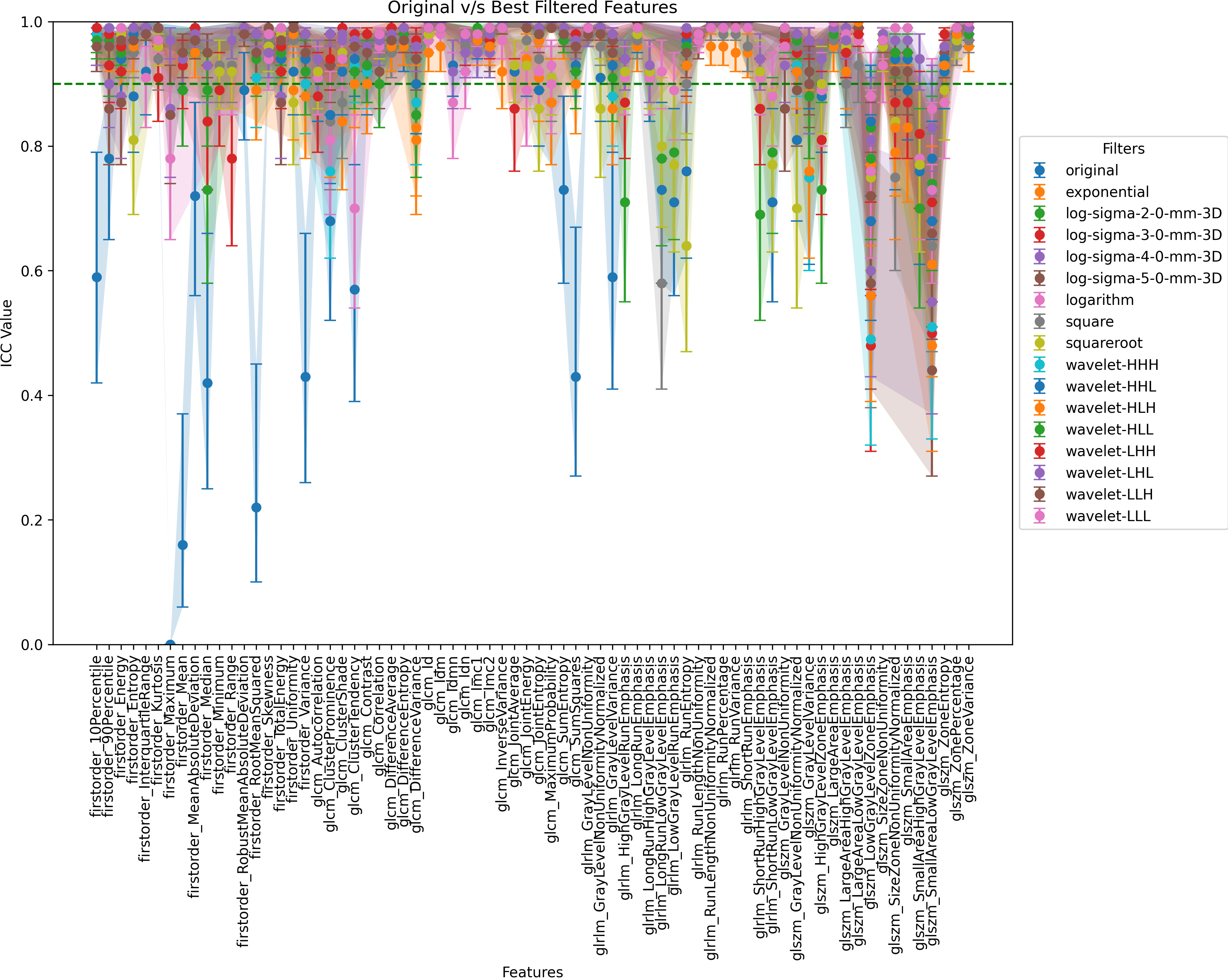

Supplement: Supplementary file 1 [file jpm-13-01172-s001.zip › plots/t2w/inout_plane_systematic_external.png]

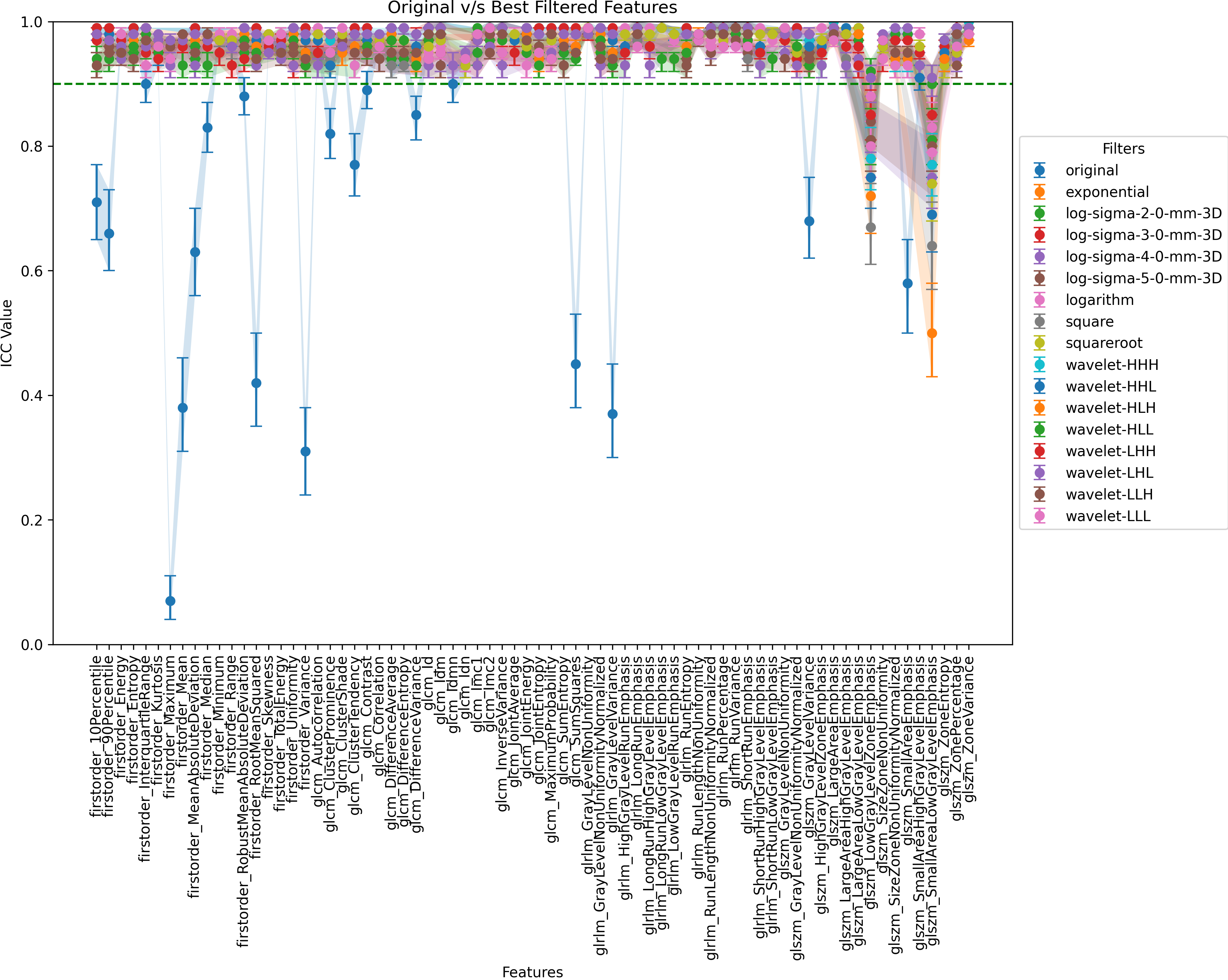

Supplement: Supplementary file 1 [file jpm-13-01172-s001.zip › plots/t2w/inout_plane_systematic_internal.png]

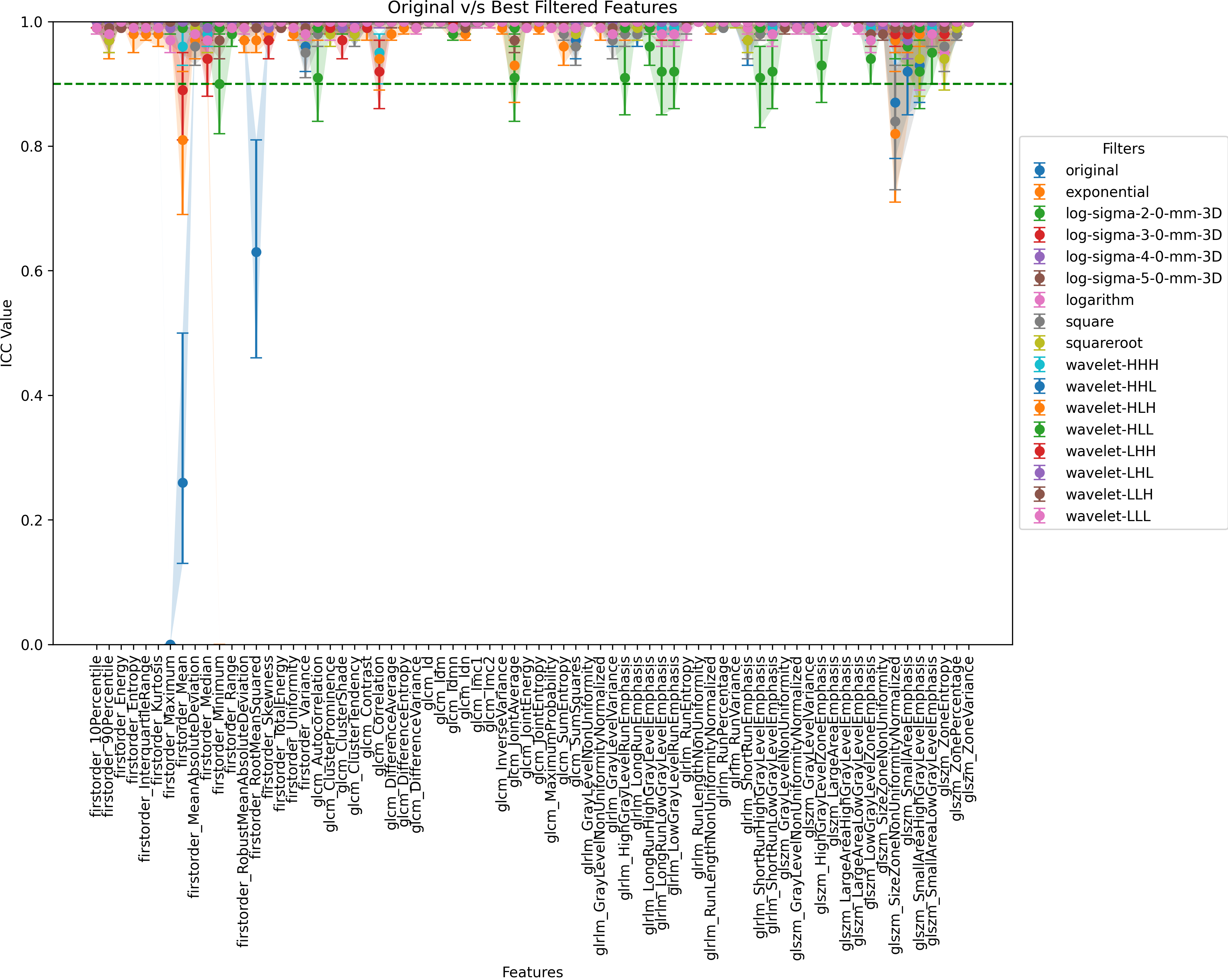

Supplement: Supplementary file 1 [file jpm-13-01172-s001.zip › plots/t2w/out_plane_external.png]

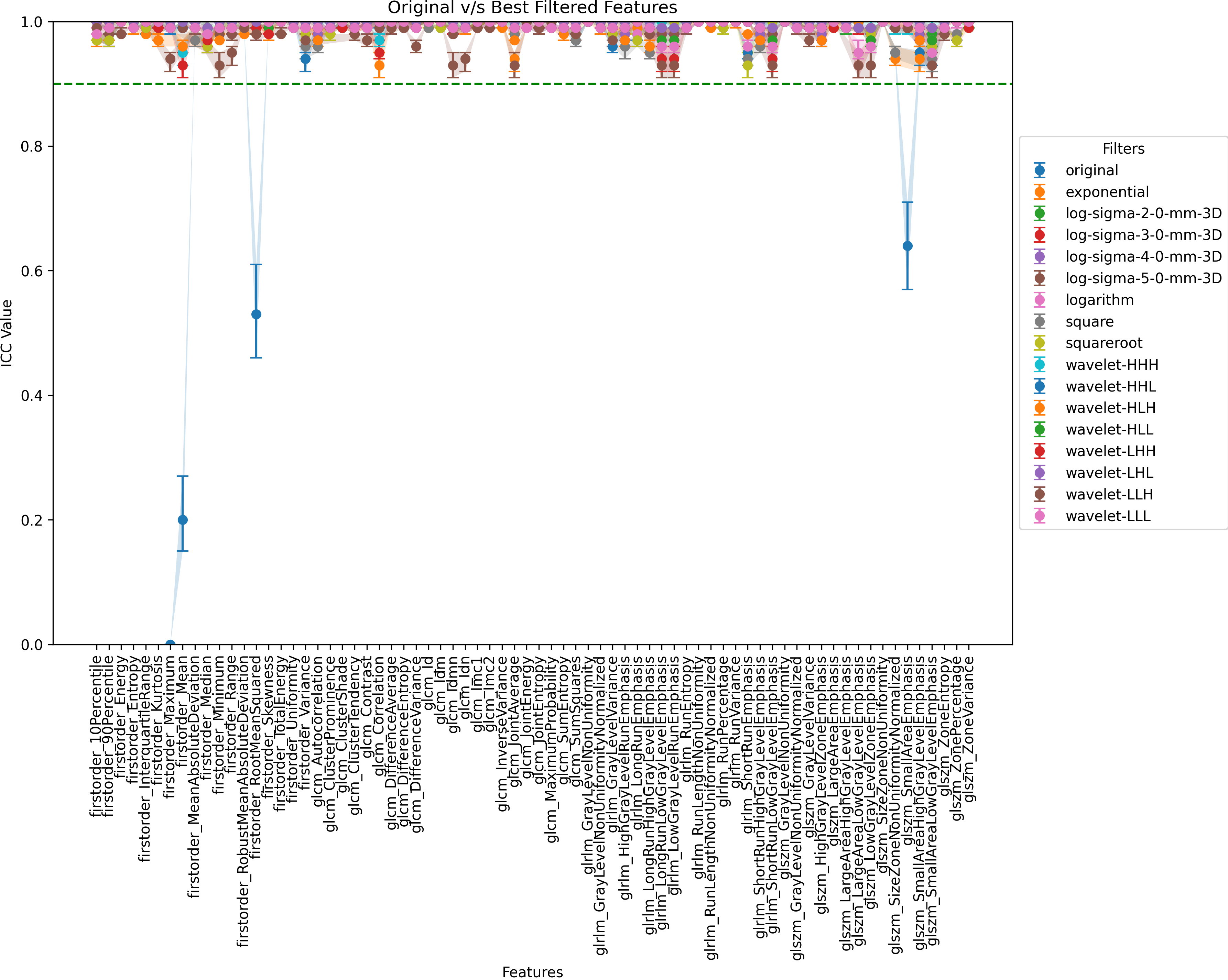

Supplement: Supplementary file 1 [file jpm-13-01172-s001.zip › plots/t2w/out_plane_internal.png]
